# Antibody signatures in patients with histopathologically defined multiple sclerosis patterns

**DOI:** 10.1007/s00401-019-02120-x

**Published:** 2020-01-16

**Authors:** Lidia Stork, David Ellenberger, Klemens Ruprecht, Markus Reindl, Tim Beißbarth, Tim Friede, Tania Kümpfel, Lisa A. Gerdes, Mareike Gloth, Thomas Liman, Friedemann Paul, Wolfgang Brück, Imke Metz

**Affiliations:** 1 Institute of Neuropathology, University Medical Center Göttingen, Robert-Koch-Strasse 40, 37075 Göttingen, Germany; 2 Department of Medical Statistics, University Medical Center Göttingen, Göttingen, Germany; 3 Department of Neurology, Charité – Universitätsmedizin Berlin, Corporate Member of Freie Universität Berlin, Humboldt-Universität Zu Berlin, and Berlin Institute of Health, Berlin, Germany; 4 Clinical Department of Neurology, Medical University of Innsbruck, Innsbruck, Austria; 5 Institute of Medical Bioinformatics, University Medical Center Göttingen, Göttingen, Germany; 6 Institute of Clinical Neuroimmunology, University Hospital and Biomedical Center, Ludwig-Maximilians University Munich, Munich, Germany; 7 Experimental and Clinical Research Center, Max Delbrück Center for Molecular Medicine and Charité Universitätsmedizin Berlin, Berlin, Germany; 8 NeuroCure Clinical Research Center, Charité Universitätsmedizin Berlin, Berlin, Germany

**Keywords:** Multiple sclerosis, Pathological patterns, Baló’s concentric sclerosis, Peptide microarray

## Abstract

Early active multiple sclerosis (MS) lesions can be classified histologically into three main immunopathological patterns of demyelination (patterns I–III), which suggest pathogenic heterogeneity and may predict therapy response. Patterns I and II show signs of immune-mediated demyelination, but only pattern II is associated with antibody/complement deposition. In pattern III lesions, which include Baló’s concentric sclerosis, primary oligodendrocyte damage was proposed. Serum antibody reactivities could reflect disease pathogenesis and thus distinguish histopathologically defined MS patterns. We established a customized microarray with more than 700 peptides that represent human and viral antigens potentially relevant for inflammatory demyelinating CNS diseases, and tested sera from 66 patients (pattern I *n* = 12; II *n* = 29; III *n* = 25, including 8 with Baló’s), healthy controls, patients with Sjögren’s syndrome and stroke patients. Cell-based assays were performed for aquaporin 1 (AQP1) and AQP4 antibody detection. No single peptide showed differential binding among study cohorts. Because antibodies can react with different peptides from one protein, we also analyzed groups of peptides. Patients with pattern II showed significantly higher reactivities to Nogo-A peptides as compared to patterns I (*p* = 0.02) and III (*p* = 0.02). Pattern III patients showed higher reactivities to AQP1 (compared to pattern I *p* = 0.002, pattern II *p* = 0.001) and varicella zoster virus (VZV, compared to pattern II *p* = 0.05). In patients with Baló’s, AQP1 reactivity was also significantly higher compared to patients without Baló’s (*p* = 0.04), and the former revealed distinct antibody signatures. Histologically, Baló’s patients showed loss of AQP1 and AQP4 in demyelinating lesions, but no antibodies binding conformational AQP1 or AQP4 were detected. In summary, higher reactivities to Nogo-A peptides in pattern II patients could be relevant for enhanced axonal repair and remyelination. Higher reactivities to AQP1 peptides in pattern III patients and its subgroup of Baló’s patients possibly reflect astrocytic damage. Finally, latent VZV infection may cause peripheral immune activation.

**Electronic supplementary material:**

The online version of this article (10.1007/s00401-019-02120-x) contains supplementary material, which is available to authorized users.

## Introduction

Multiple sclerosis (MS) is an inflammatory demyelinating CNS disease with heterogeneous clinical, radiological and pathological features that suggest different mechanisms of disease development. An accurate diagnosis is important from disease onset, as the correct diagnosis has a prognostic value and helps inform treatment strategy [9, 18, 25]. However, the individual disease course, disease progression or response to therapies in MS are not yet predictable. Multiple drugs are available for MS treatment [56, 57], but we still lack biomarkers for stratification of particular subgroups of MS patients and specific pathogenic pathways.

**Fig. 1:**
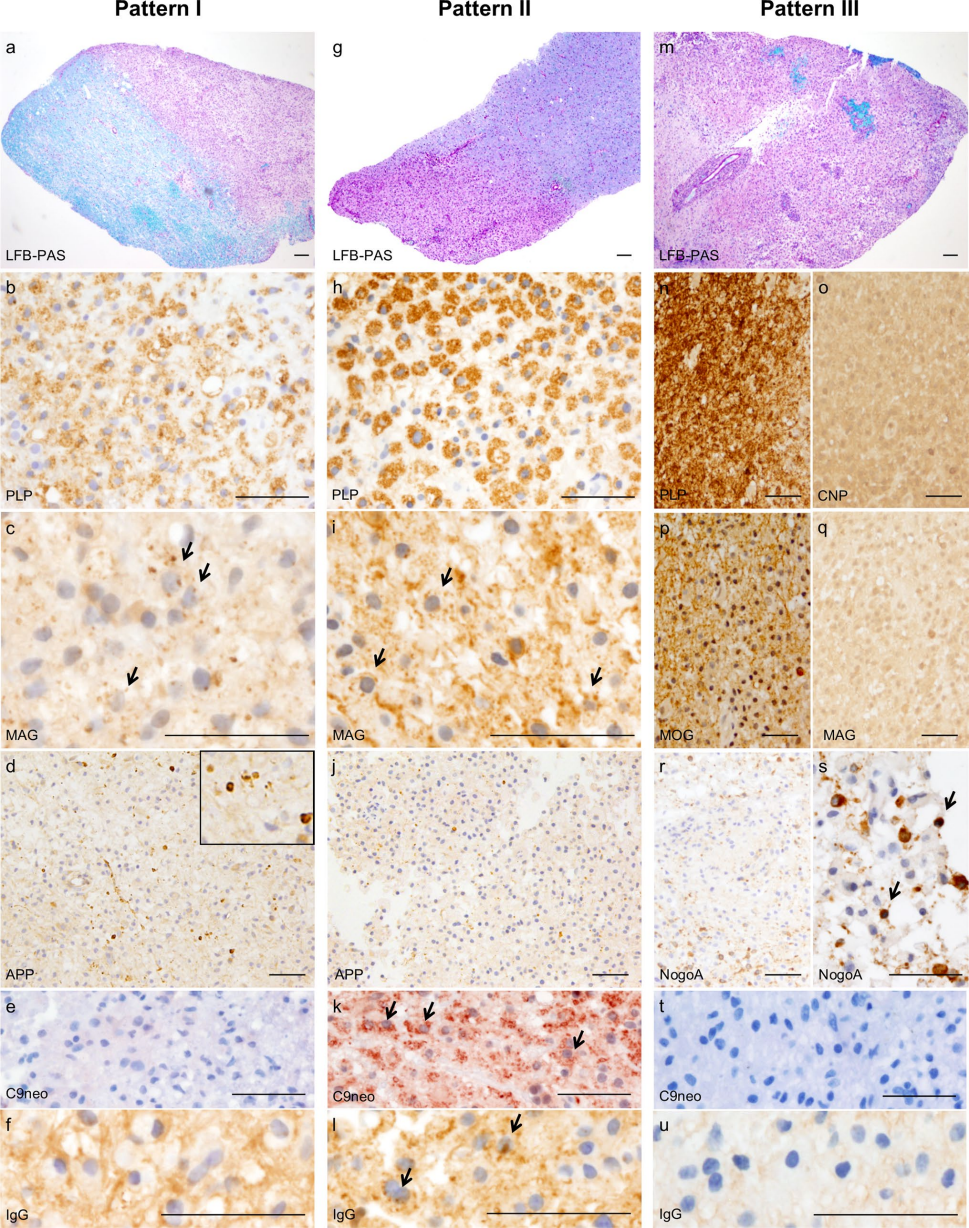
Histopathology of the three immunopathological patterns of early active inflammatory demyelinating lesions. Histopathological characteristics of pattern I lesions (**a**–**f**): **a** demyelinating pattern I lesion with a sharp lesion border, as shown with the myelin staining luxol fast blue/periodic acid shift (LFB/PAS, myelin shown in blue); **b** the active demyelinating lesion contains numerous of macrophages with myelin degradation products incorporated within the cytoplasm (PLP staining, major myelin protein, macrophages are filled with brown myelin degradation products); **c** active demyelinating lesion also shows numerous MAG-positive macrophages (MAG staining, minor myelin protein, macrophages with incorporated myelin degradation products indicated with arrows); **d** numerous acutely damaged axons are present within the lesion (APP staining, small brown dots show axonal spheroids); **e** absence of complement products within macrophages in pattern I lesions (negative C9neo staining); **f** absence of IgG deposits within macrophages in pattern I lesions (negative IgG staining). Histopathological characteristics of pattern II lesions (**g**–**l**): **g** demyelinating pattern II lesion with a sharp lesion border, as indicated with the LFB/PAS staining; **h** active demyelinating lesion with numerous macrophages with PLP-positive degradation products (PLP staining) and **i** MAG-positive macrophages (MAG staining, positive macrophages indicated with arrows); **j** some acutely damaged axons are seen in the lesion (APP staining); **k** the hallmark of pattern II lesions are activated complement products within macrophages (C9neo staining, positive macrophages are indicated with arrows) as well as **l** IgG deposits within macrophages (IgG staining, positive macrophages are indicated with arrows), suggesting that the humoral immune response plays an important role in lesion development in pattern II lesions. Histopathological characteristics of pattern III lesions (**m**–**u**): **m** demyelinating pattern III lesion, as indicated with LFB/PAS staining; **n**–**o** areas of preserved PLP staining show loss of CNP expression in the same lesions areas (CNP-loss); **p**, **q** areas of preserved MOG expression show absence of the MAG expression in pattern III lesions (MAG-loss); **r**, **s** a reduction and apoptosis of oligodendrocytes are further hallmarks of pattern III lesions (NogoA staining, apoptotic oligodendrocytes are indicated with arrows); **t** Absence of activated complement products (C9neo staining) and u) IgG deposits within macrophages in pattern III lesions. Scale bars: **a**, **g** and **m**: 100 µm; **b**–**f**, **h**–**l**, **n**–**u**: 50 µm. *PLP* proteolipid protein, *MAG* myelin-associated glycoprotein, *APP* amyloid precursor protein, *C9neo* complement 9neo, *IgG* immunoglobulin G, *CNP* 2′,3′-cyclic nucleotide 3′-phosphodiesterase, *MOG* myelin oligodendrocyte glycoprotein

Lucchinetti et al. describe three main subgroups of MS patients that show different histopathological patterns of early active inflammatory demyelinating lesions (patterns I–III, Fig. 1) and suggest diverse pathophysiological mechanisms of lesion development [42]. These patterns are stable within the individual patient [35, 52] and imply a specific and sustained pathogenic pathway for newly developing lesions during the entire disease course of that patient. Pattern I and pattern II lesions show sharply demarcated demyelinated areas with inflammation consisting of T cells, B cells and macrophages/microglial cells. Yet only in pattern II lesions does the humoral immune system seem to be involved in lesion development, as these lesions reveal immunoglobulins and complement along myelin sheaths and inside macrophages (Fig. 1a–l). In pattern III lesions, oligodendroglial pathology with apoptotic oligodendrocytes and subsequent demyelination on an inflammatory background is present, suggesting a primary degenerative character of lesions [42] (Fig. 1 m–u).

The clinical relevance of these immunopathological patterns has been shown previously: Apheresis is a second-line therapy for MS relapses. Whereas pattern III patients do not respond to apheresis therapy, > 50% of pattern II patients benefit from this treatment [32, 75]. Thus far, patterns I–III can only be determined by histopathological analysis of brain biopsies. It is apparent that another biomarker would be preferable to distinguish these patterns, as well as to better understand the immunopathogenesis with the ultimate goal of optimizing the treatment of patients.

It is important to note that the immunopathological patterns—and thus the heterogeneity of demyelinating lesions—are found in early disease stages typically characterized by a relapsing remitting disease course. They can only be detected in the earliest lesion stages (early active demyelinating lesions) [42, 52]. In contrast, in long established MS which is typically characterized by a progressive disease course, chronic active lesions prevail. These lesions are usually immunopathologically uniform [6, 21]. Antibody- and complement-mediated myelin phagocytosis could play a role in demyelination in late disease stages [6].

Furthermore, antibody reactivities were shown to differ depending on the disease stage. Distinct antibody patterns, based on reactivity to CNS antigens and heat shock proteins, were observed in relapsing remitting MS, secondary progressive MS and primary progressive MS [60]. Antibodies directed against α-galactocerebrosides, the major glycolipid of CNS myelin, were predominant in relapsing remitting MS [50]. In contrast, an increase in circulating anti-ganglioside antibodies in primary and secondary progressive MS compared to relapsing–remitting MS has been reported [68]. Gangliosides are mainly found in axons. The authors suggested that the transition from relapsing remitting MS to secondary progressive MS could cause a spread of the immune response from myelin to axonal antigens, with the damage of axons explaining the progressive disease course [68].

**Fig. 2:**
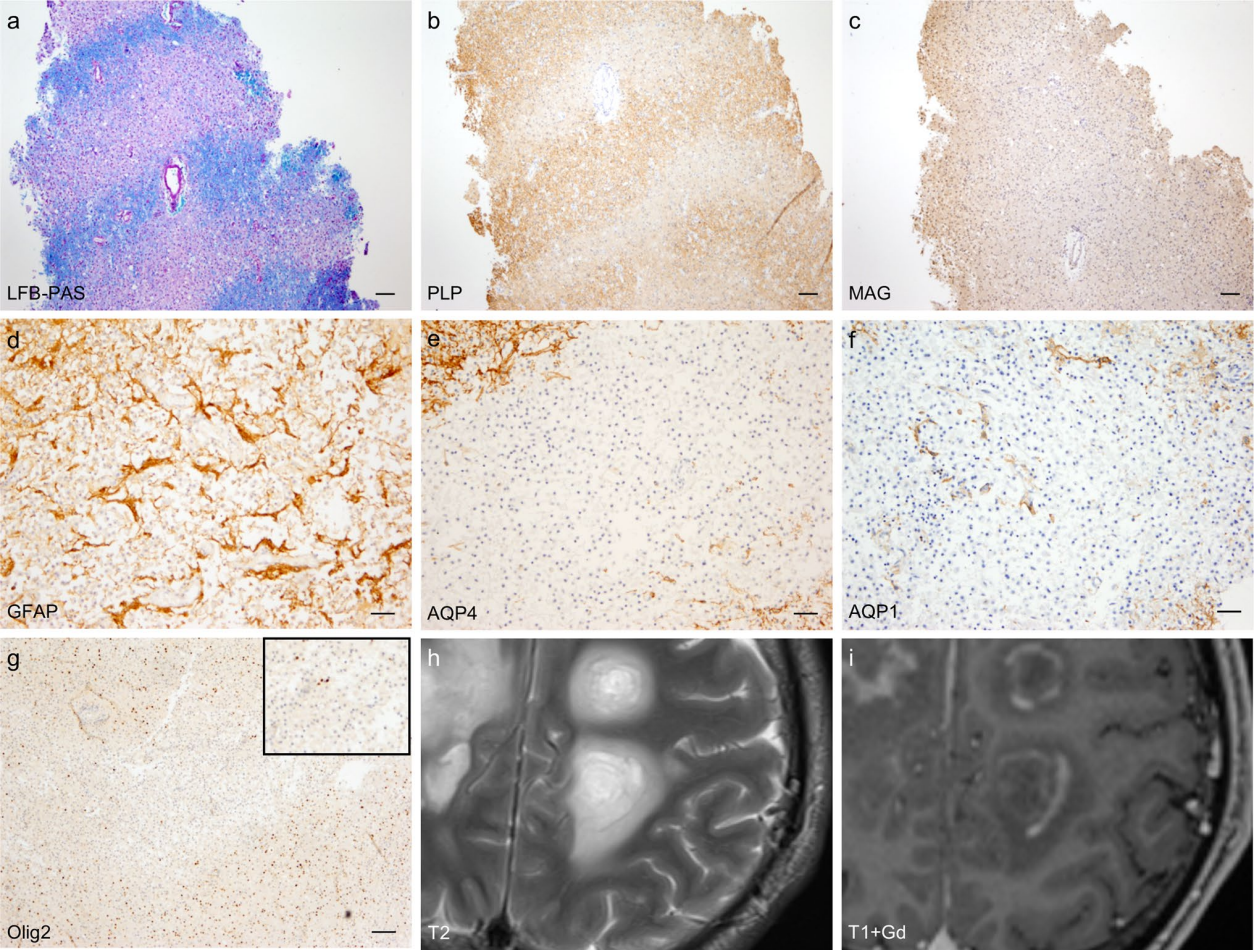
Typical histopathological and MRI findings in Baló’s concentric sclerosis. **a** Baló’s lesions are characterized by alternating areas of myelin preservation and myelin loss, as indicated with the myelin staining luxol fast blue/periodic acid shift (LFB/PAS, myelin shown in blue). **b** Correspondingly, areas of preserved PLP expression and areas of PLP loss (PLP staining) can be observed. **c** A complete loss of MAG expression (MAG-loss) in the same lesion areas is found and a characteristic feature for pattern III lesions (MAG staining). **d** A subset of Baló’s concentric sclerosis lesions show dystrophic astrocytes (GFAP staining), **e** loss of AQP4 expression (AQP4 staining) and **f** loss of AQP1 expression (AQP1 staining). **g** Notably, a reduction of oligodendrocytes in the Baló’s lesions can be observed (Olig2 staining, inset with oligodendrocyte loss in higher magnification). **h** MRI shows lesions with T2 hyperintensive alternating concentric rings (T2-weighted images) and **i** concentric rings of contrast enhancement (T1 weighted + Gd). Scale bars: **a**–**c**: 100 µm; **d**–**g**: 50 µm. *PLP* proteolipid protein, *MAG* myelin-associated glycoprotein, *GFAP* glial fibrillary acidic protein, *AQP1(4)* aquaporin 1(4), *Gd* gadolinium

Baló’s concentric sclerosis is a rare MS variant characterized by alternating rings of demyelination and areas of myelin preservation [27, 73]. Baló lesions show pattern III characteristics featuring MAG loss and apoptotic oligodendrocytes (Fig. 2a–c, g). However, astrocytic changes with a reduction of aquaporin 4 (AQP4) staining have also been described [47]. Radiologically, this type of MS can be identified by white matter lesions with hyperintense and isointense concentric lamellae seen on T2-weighted (T2W) and sometimes on T1-weighted gadolinium-enhanced (T1 + Gd) images [2, 14, 80] (Fig. 2h, i).

Neuromyelitis optica spectrum disorders (NMOSD) were the first inflammatory demyelinating diseases characterized by specific antibodies [37]. Antibodies directed against the water channel AQP4 are used as a diagnostic biomarker and play a pathogenic role in lesion development [4, 38, 82]. Several lines of evidence suggest that antibodies are also involved in lesion development in MS [32, 42, 48, 75]. Indeed, antibodies binding the myelin oligodendrocyte glycoprotein (MOG) were identified in a small subgroup of MS patients [22, 63, 72]. However, no specific antibodies were otherwise found. It is conceivable that not a single antibody, but a specific combination of autoimmune responses directed against CNS antigens could characterize subgroups of MS.

We designed a specific peptide antigen microarray that included human and viral antigens of potential relevance for inflammatory demyelinating CNS diseases. Peptides were preselected from a total of 8,708 peptides containing antigens chosen by an extensive literature search as well as mimotopes, random epitopes that were used for an unbiased approach (for details see Metz et al. [51]). These preselected peptides, peptides of special interest (e.g. MOG peptides) as well as control peptides resulted in a total of 702 peptides that were used for our customized peptide microarray. A full list of included peptides is given in Supplementary Table 1, online resource. With this array, differential peptide reactivities distinguished between NMOSD and relapsing–remitting MS (RRMS) in 80% of patients, and also identified higher reactivities to myelin and Epstein-Barr virus peptides in relapsing–remitting MS (RRMS) compared to NMOSD [51]. In the present study, we used this customized peptide microarray to analyze specific antibody profiles in different histopathologically characterized patterns of MS. These antibody profiles may serve as biomarkers and identify potential pathogenic mechanisms.

**Table 1:** Demographical and clinical data of the study cohort

	Pattern I (*n* = 12)	Pattern II (*n* = 29)	Pattern III (*n* = 25)	*p* value All patterns	Baló’s concentric sclerosis (subgroup of pattern III) (*n* = 8)^a^	Non-Baló’s patients (*n* = 53)^a^	*p* value Baló’s vs non-Baló’s
Age in years, mean (SD)	47.4 (17.5)	42.3 (16.6)	39.8 (17.2)	0.43	38.0 (20.2)	43.9 (16.4)	0.33
Proportion of females, (%)	6/12 (50)	20/29 (69)	11/25 (75)	0.2	5/8 (62.5)	31/5 (58.5)	0.27
Disease course: single clinical episode (%)	8/12 (66.7)	18/29 (62.1)	20/25 (80)	0.51	7/8 (87.5)	34/53 (64.2)	0.7
Disease course: RR (%)	4/12 (33.3)	9/29 (31)	4/25 (16)		1/8 (12.5)	16/53 (30.2)	
Disease course: SP (%)	0/12 (0)	2/29 (6.9)	1/25 (4)		0/8 (0)	3/53 (5.6)	
Disease duration in months: median (range)	8.9 (− 11.2^b^; 42.8)	9.57 (2.83; 231)	6.87 (2.93; 401)	0.74	7.13 (3.8; 34.3)	9.53 (− 11.2^b^; 401)	0.7
Time interval from biopsy to first blood sampling in months median (range)	4.98 (0.00; 15.7)	7.07 (2.00; 32.3)	4.77 (0.30; 26.3)	0.28	4.75 (0.30; 11.5)	6.30 (0.00; 32.3)	0.4
Relapse therapy within 1 month prior to blood sampling and MS-specific therapy at the time of blood sampling, n	PLEX/IA: 3; HDCS: 1; RTX: 1; CPM: 1; AZA: 1	RTX: 1; GA: 2; MTX: 1; IFN: 2; FTY: 1; DMF: 2; ALZ: 1	RTX: 1; CPM: 4; IFN: 1; TER: 1; ALZ: 1, NTZ: 1; LDCS: 2		LDCS: 2	PLEX/IA: 3; HDCS: 1; RTX:3; CPM: 4; AZA: 1 GA: 2; MTX: 1; INF: 1; FTY: 1; DMF: 2; ALZ: 2, TER: 1	

*SD* standard deviation, *RR* relapsing–remitting multiple sclerosis, *SP* secondary progressive multiple sclerosis, *PLEX* therapeutic plasma exchange, *IA* immunoadsorption, *HDCS* high dose of corticosteroids, *LDCS* low dose of corticosteroids, *RTX* rituximab, *CPM* cyclophosphamide, *INF* interferon, *AZA* azathioprine, *FTY* fingolimod, *MTX* mitoxantrone, *ALZ* alemtuzumab, *NTZ* natalizumab, *DMF* dimethylfumarate, *TER* teriflunomide

^a^Five patients were excluded from the analyses as they could not be classified with certainty as having Baló’s concentric sclerosis

^b^One patient with melanoma in the anamnesis was biopsied for diagnostic purposes, but had no clinical symptoms

## Materials and methods

### Study cohort and serum sampling

This study was approved by the ethics committee of the University Medical Center Göttingen (#19/09/10). Patients were recruited at the Institute of Neuropathology, University Medical Center Göttingen. Serum samples were all collected in the standard manner by the Institute of Neuropathology during baseline study examination, except four samples provided by the Institute of Clinical Neuroimmunology Munich. Serum control samples were provided by the Department of Neurology, Charité – Universitätsmedizin Berlin. All patients gave written informed consent. The study cohort included patients with a histopathological diagnosis of inflammatory demyelinating lesions consistent with MS (referred to as MS patients throughout this manuscript). Only patients with biopsies showing early active demyelinating lesions, representing the earliest lesions stages and classified into one of the main immunopathological patterns I–III, were included in the present study [42]. Brain biopsies were performed for clinical differential diagnostics and sent to the Institute of Neuropathology for a second opinion. No subjects underwent surgery for research purposes. Patients with acute disseminated encephalomyelitis (ADEM) [83] and NMOSD [10] were excluded from the study.

We analyzed serum from 66 biopsied MS patients (pattern I *n* = 12; pattern II *n* = 29; pattern III *n* = 25), with eight of them showing histological and/or MRI characteristics of Baló’s concentric sclerosis. Blood sampling was performed after a median of 5.7 months (range 0.0–32.3) after the brain biopsy. A second blood sample, taken approximately 1 year after the first one (median 12.0 months, range 4.8–20.7), were available for 30 MS patients (pattern I *n* = 9, II *n* = 10, III *n* = 11). Control serum samples were collected from 15 healthy controls, 15 patients with Sjögren’s syndrome (representing a peripheral autoimmune disorder) and 15 patients with a stroke (representing a non-inflammatory central nervous system disease). Stroke patients were diagnosed according to the WHO criteria. Patients showed a median stroke severity at onset (NIHSS – National Institutes of Health Stroke Scale) of 5 (IQR 2–8). Blood was taken after a mean of 4.4 days after onset of stroke symptoms. Demographic data for control subjects are provided in Supplementary Table 2, online resource.

### Histological and radiological classification of brain lesions

Histopathological classification of brain lesions was performed by two board-certified neuropathologists (WB, IM) according to published criteria [42]. Formalin-fixed, paraffin-embedded tissue was characterized by histological and immunohistochemical stainings presented in Supplementary Table 3, online resource. Only early active demyelinating lesions, characterized by the presence of macrophages with both minor [2′3′-cyclic nucleotide 3′ phosphodiesterase (CNP), myelin-associated glycoprotein (MAG), MOG] and major [proteolipid protein (PLP), myelin basic protein (MBP)] myelin proteins incorporated within the cytoplasm represent the earliest lesion stages and can be used for classification into one of the immunopathological patterns I–III [11, 42]. In addition, we classified patients as having Baló’s concentric sclerosis when they showed evidence of concentric demyelination—either histologically with repeated areas of concentric myelin preservation and loss and/or radiologically with alternating hyperintense and isointense concentric lamellae seen on T2-weighted (T2W) and/or on T1-weighted gadolinium-enhanced (T1 + Gd) sequences [14, 73, 80]. Five patients could not certainly be allocated as having Baló’s concentric sclerosis or not and were thus excluded from the analyses comparing Baló subgroups. Finally, AQP4 (*n* = 60), aquaporin 1 (AQP1) (*n* = 27) and varicella zoster virus (VZV, *n* = 3) immunohistochemistry was performed in a subset of patients.

### Clinical follow-up

Clinical data were collected in face-to-face examinations in the Institute of Neuropathology and from medical records. Diagnosis at the time of blood sampling was based on the 2017 McDonald criteria for MS [76]. Clinical course was classified as single clinical episode (clinically isolated syndrome, CIS), relapsing–remitting, secondary progressive or primary progressive MS [41]. Expanded disability status score (EDSS) at the time of blood sampling was obtained during the face-to-face examination (*n* = 50) or extracted from the patients’ clinical records (*n* = 16).

To analyze possible effects of MS treatments on antibody signatures, therapy at the time of blood sampling was recorded. Therapies were classified according to their assumed effect on immunoglobulin G (IgG) levels in serum as (1) reduction of antibody levels probable/shown in prior studies, (2) possible/mild reduction of IgG levels and (3) no reduction of antibody levels expected. Treatment with high dose corticosteroids (HDCS) and plasma exchange (PLEX) and/or immunoadsorption (IA) within 1 month before blood sampling were considered to likely reduce antibody levels [12, 35, 49, 59]. Therapy with rituximab, cyclophosphamide, azathioprine, mitoxantrone and teriflunomide was considered to have a possible effect [40], and treatment with interferons, dimethyl fumarate, fingolimod, glatiramer acetate, natalizumab and alemtuzumab was assumed to have no effect on antibody levels in serum [36, 67].

Additional information about VZV IgG and immunoglobulin M (IgM) antibody titers in serum and cerebral spinal fluid (CSF), VZV PCR diagnostics in CSF, as well as clinical evidence for a herpes zoster infection at the time of blood sampling was extracted from the clinical records of 14 patients with pattern I and III histology.

### Peptide microarray

We used a customized peptide microarray setup in prior studies that includes 702 peptide antigens representing human and viral antigens potentially relevant for inflammatory demyelinating diseases, as well as random peptides (mimotopes) and controls (for more information see [51] and Supplementary Table 1, online resource). Microarrays were produced with a peptide laser printer and amino acid particles for a combinatorial synthesis of peptides (PEPperPRINT, Heidelberg, Germany) [5].

### Microarray staining and reading

In short, arrays were incubated with a DyLight 549 conjugated goat anti-human immunoglobulin G (IgG) antibody (diluted 1:1000; anti-human IgG [H&L] goat antibody, Rockland, Gilbertsville, PA) for 30 min and scanned to rule out relevant background interactions. Arrays were then incubated overnight with serum samples diluted 1:1000, followed by staining with the secondary antibody (DyLight 549 conjugated goat anti-human IgG). C-myc control epitopes were printed on the microarray in a square surrounding the other peptides and stained with corresponding antibodies (chimeric human IgG1 anti-myc (Chi9E10) antibody, provided by PEPperPRINT, Heidelberg, Germany). The array was finally read with a Fujifilm Life Science (Stamford, CT) FLA-imaging system using a second harmonic generation 532 nm (green) diode laser and LPG filter. Quantification of spot intensities and peptide annotation were done with PepSlide Analyzer (PEPperPRINT). Results are expressed as fluorescence intensity units, which reflect the extent of the antibody binding to the selected peptide. For more detailed information see [51].

### Cell-based AQP1 and AQP4 assay

Cell-based assays (CBA) for AQP1 and AQP4 antibodies were performed for selected patients (*n* = 29), including patients with the highest reactivities to AQP1 and AQP4 peptides as well as all patients with Baló’s concentric sclerosis. Patients with Baló’s were included, because a high binding of AQP1 peptides was observed in our analyses (see results) and because an AQP4-loss in histological sections in Baló’s concentric sclerosis patients had previously been described [47]. Anti-AQP4 antibodies as identified in cell-based assays are used for diagnostics in NMOSD.

Analysis of AQP1 antibodies and AQP4 antibodies was performed using a live CBA described previously [31, 44]. Briefly, HEK293A cells were transiently transfected using the pcDNA6.2C-EmGFP-GW/TOPO plasmid (Invitrogen, Carlsbad, CA), expressing AQP4 (isoform M23) or AQP1 (isoform 1) fused C-terminally to emerald green fluorescence protein. Transfected cells were blocked with goat IgG in phosphate-buffered saline (PBS)/10% fetal calf serum (FCS) (Sigma-Aldrich, St. Louis, MO) followed by serum diluted 1:20 and 1:40 in PBS/FCS for 1 h at 4 °C. Serum preabsorption with liver powder was not performed. Bound antibodies were detected using Cy3Tm-conjugated goat anti-human IgG antibody (Jackson ImmunoResearch Laboratories, West Grove, PA) for 30 min at room temperature. Bound antibodies were determined using a fluorescence microscope (Leica DMI 4000B). All samples were evaluated by two independent, clinically blinded investigators who agreed on all samples.

### Sample size/power calculation

The sample size for this research project was chosen based on results from a first validation by Quintana et al. [50]. Sample size planning required recruitment of *n* = 75 patients for this purpose, taking possible dropouts also into consideration. A sample size of 25 patients per group provides a power of at least 80% for a two-sided t-test at a significance level of 5%, as long as the standardized difference between groups is at least 0.8. Fisher’s exact test at a two-sided significance level of 5% has a power of 94% if the response rates in the groups are 25% and 75%, which is a conservative estimate.

### Statistical analysis

Demographics and clinical characteristics were described by summary statistics appropriate for their scales. Group differences in clinical and expression data were tested using Welch’s *t* test or Wilcoxon test, as appropriate. To test for pairwise differences of single peptides, an empirical Bayes method to analyze microarray data using linear models to assess differential expression (limma method) was used. MA plots (i.e., Bland–Altman plots) were applied to visualize differential peptide reactivities between pairs of groups. Interactions between peptides of a certain subset and patient subgroups were assessed with global tests. Global tests were performed according to Jung et al. [29] and Goeman et al. [24]. Furthermore, to identify groups of peptides showing significantly higher or lower antibody reactivities, gene-set-enrichment analyses were performed to compare histopathological patterns. For the gene set enrichment analysis, the p-value from the limma method for each peptide was used and ranked over all peptides. To compare a specific peptide set against the other peptides, a Wilcoxon test was applied. Peptide groups which showed statistically significant differences in both tests were considered robust results and are presented here.

### Processing of data

Reactivity of duplicate measurements showed a strong correlation of median intensities (*r* = 0.84), indicating good reliability of results. However, due to inhomogeneous base levels of peptide/background reactivities, a normalization of data using control peptides spotted on the arrays had to be performed. In a next step, 662 informative peptides, for which at least 5% of the participants had normalized intensity values greater than log2 (1000 arbitrary intensity units), were selected for further analyses. When testing within the (high-dimensional) peptide set was done, Benjamini and Hochberg adjustment (fdr) for multiple testing was performed. Due to the exploratory character of the study, we did not adjust for multiple (patient) group comparisons, and the significance threshold was always set at 5%.

## Results

### Demographic and clinical characteristics of MS cohorts

Demographics and basic clinical data of 66 biopsied patients, stratified by their immunopathological patterns and by their Baló’s concentric sclerosis status, are summarized in Table 1. The groups showed no differences for the parameters listed. We focused on early disease stages with a median disease duration of less than 1 year. At the time of first blood sampling, 29 of 66 patients had clinically definitive MS according to the revised McDonald 2017 criteria [76]. More than one-third of the patients (*n* = 24) were being treated with MS-specific therapies at this time point (Table 1). Three patients had an acute relapse therapy within 1 month prior to the blood sampling (HDCS or PLEX/IA). One patient was treated with cyclosporine due to liver transplantation and another patient received ipilimumab, an antibody targeting cytotoxic T-lymphocyte-associated protein 4 (CTLA-4), to treat melanoma.

### Antibody reactivities are more frequent in Sjögren’s syndrome (as an example of a peripheral autoimmune disease) compared to MS subgroups

First, we aimed to compare antibody reactivities of MS patterns I–III with patients with (1) Sjögren’s syndrome as an example of a peripheral autoimmune disease with known multiple antibody reactivities [17], (2) with stroke as an example of a non-autoimmune CNS disease, and (3) healthy controls. Antibody reactivities were measured by fluorescence intensities which reflect antibody binding to the selected peptides.

For this purpose, we compared antibody reactivities to single peptides (*n* = 662). Patients with Sjögren’s syndrome showed numerous differentially bound peptides when compared to MS patterns I–III (pattern I *n* = 225; pattern II *n* = 301; pattern III *n* = 278 differentially bound peptides, for complete list see Supplementary Table 4, online resource). Most of the reactivities were higher in Sjögren’s syndrome, indicating higher antibody reactivities in this peripheral autoimmune disease (compared to pattern I *n* = 220/225, pattern II *n* = 251/301 and pattern III *n* = 217/278 peptides with higher reactivities in Sjögren’s syndrome). Differentially bound peptides belonged to different proteins, suggesting a broad upregulation of IgG reactivities. In contrast, no differentially bound peptides were found when comparing MS patterns I–III and stroke patients or healthy controls.

### No differences in single peptide reactivities are present among MS patterns

Next, we compared the three MS study groups, divided according to their immunopathological pattern for differences in single peptide reactivities; we did not find any differentially bound peptides after adjustment for multiple testing.

### Higher reactivities to AQP1 and VZV peptides are found in pattern III patients

We assumed that instead of analyzing reactivities to single peptides, reactivities to a group of peptides belonging to one protein (for example MOG) or a protein group (for example, myelin proteins) could be more informative and potentially point to proteins involved in disease pathogenesis. For this purpose we compared the IgG reactivities to the following peptide groups: Epstein–Barr-Virus (EBV), VZV, cytomegalovirus (CMV), AQP1, AQP4, Nogo-A, all myelin proteins, neurofascin, neurofilament, Kir4.1, amyloid beta, contactin-2, AN-2, hypoxia inducible factor 1 alpha (HIF-1α), heat shock protein 60 (HSP60), HSP70, and peptides published as differentially regulated between patterns I and II by Quintana et al. in 2008 [HSP60, MOG, oligodendrocyte specific protein (OSP), PLP] [60].

**Fig. 3:**
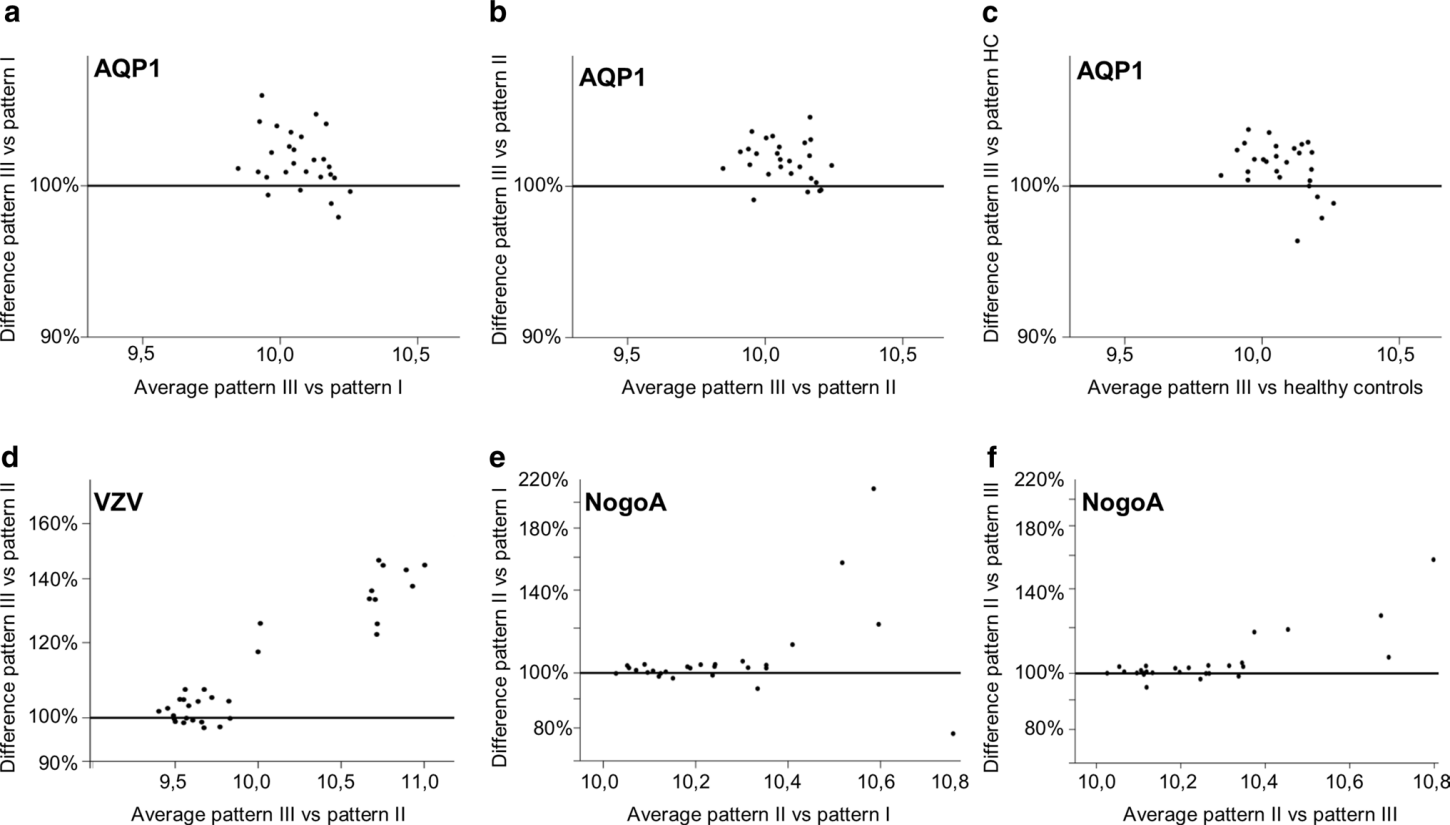
Binding of selected peptide groups comparing immunopathological MS patterns and shown with Bland–Altman plots. **a**–**c** AQP1 peptide reactivities in pattern III vs pattern I, II and healthy controls; **d** VZV peptide reactivities in pattern III vs pattern II; **e**, **f** NogoA peptide reactivities in pattern II vs pattern I and pattern III. Each dot represents one peptide. The x-axis displays the average normalized signal to show the general level of peptide-binding reactivity. The y-axis indicates the differences between the patient groups given as a percentage. Greater reactivities in the first-mentioned group are found above the 100% horizontal line, and lower reactivities below this line. Patients with pattern III lesions showed higher reactivities to AQP1 peptides as compared to pattern I and II patients as well as healthy controls (**a**–**c**). These patients also revealed higher binding reactivities to VZV peptides as compared to pattern II patients (**d**). Patients with pattern II lesions showed higher reactivities to NogoA peptides as compared to pattern I and pattern III patients (**e**, **f**). *AQP1* aquaporin 1, *VZV* varicella zoster virus

Higher reactivities directed against the astrocytic water channel AQP1 were detected in pattern III patients compared to pattern I patients (*p* values are given for the global tests, *p* < 0.01, Fig. 3a), pattern II patients (*p* < 0.01, Fig. 3b) and healthy controls (*p* = 0.03, Fig. 3c).

In accordance with these findings, in some pattern III lesions (lesions from Baló’s patients) an AQP1 loss was found in demyelinating lesions (see results below and Supplementary Fig. 1, online resource).

To further test AQP1 antibody reactivities, sera with the highest binding reactivities found for AQP1 with our peptide microarray taken from 19 pattern III lesions (including from patients with Baló’s concentric sclerosis) and ten pattern II patients without any histological evidence of astrocytopathy were tested for antibodies with a cell-based assay recognizing conformational AQP1 as well as AQP4. No anti-AQP1- or anti-AQP4-antibodies were found with this assay.

Also, higher reactivities against VZV peptides were found in pattern III patients than in pattern II patients (*p* = 0.05, Fig. 3d). No statistically significant difference was found compared to pattern I patients.

No evidence of acute VZV infection was found in pattern III patients. We performed immunohistochemical stainings for VZV in three pattern III brain biopsies with the highest VZV reactivity in our peptide microarray, all with negative results. Furthermore, out of 14 pattern III patients with elevated VZV reactivities in our peptide microarray, clinical records for evaluation for VZV infection were available from 11 patients. All patients did not reveal serological and/or CSF evidence for acute VZV infection.

### Pattern II patients show higher reactivities against Nogo-A peptides

Nogo-A is expressed mostly in oligodendrocytes, but also neurons. Pattern II patients showed higher reactivities against Nogo-A peptides compared to pattern I patients (*p* = 0.02, Fig. 3e) and pattern III patients (*p* = 0.02, Fig. 3f).

As Nogo-A antibodies may promote tissue repair, we also compared the clinical disability, as measured with the EDSS, in pattern II versus pattern I and pattern III patients. At the time of blood sampling, a median EDSS of 3.0 was found in pattern II patients (with the highest Nogo-A reactivities) and of 8.5 in pattern I and pattern III patients (with the lowest reactivities to Nogo-A peptides), but differences were not significant (*p* = 0.09). Also, no significant differences in the median disease duration were seen among these groups.

**Fig. 4:**
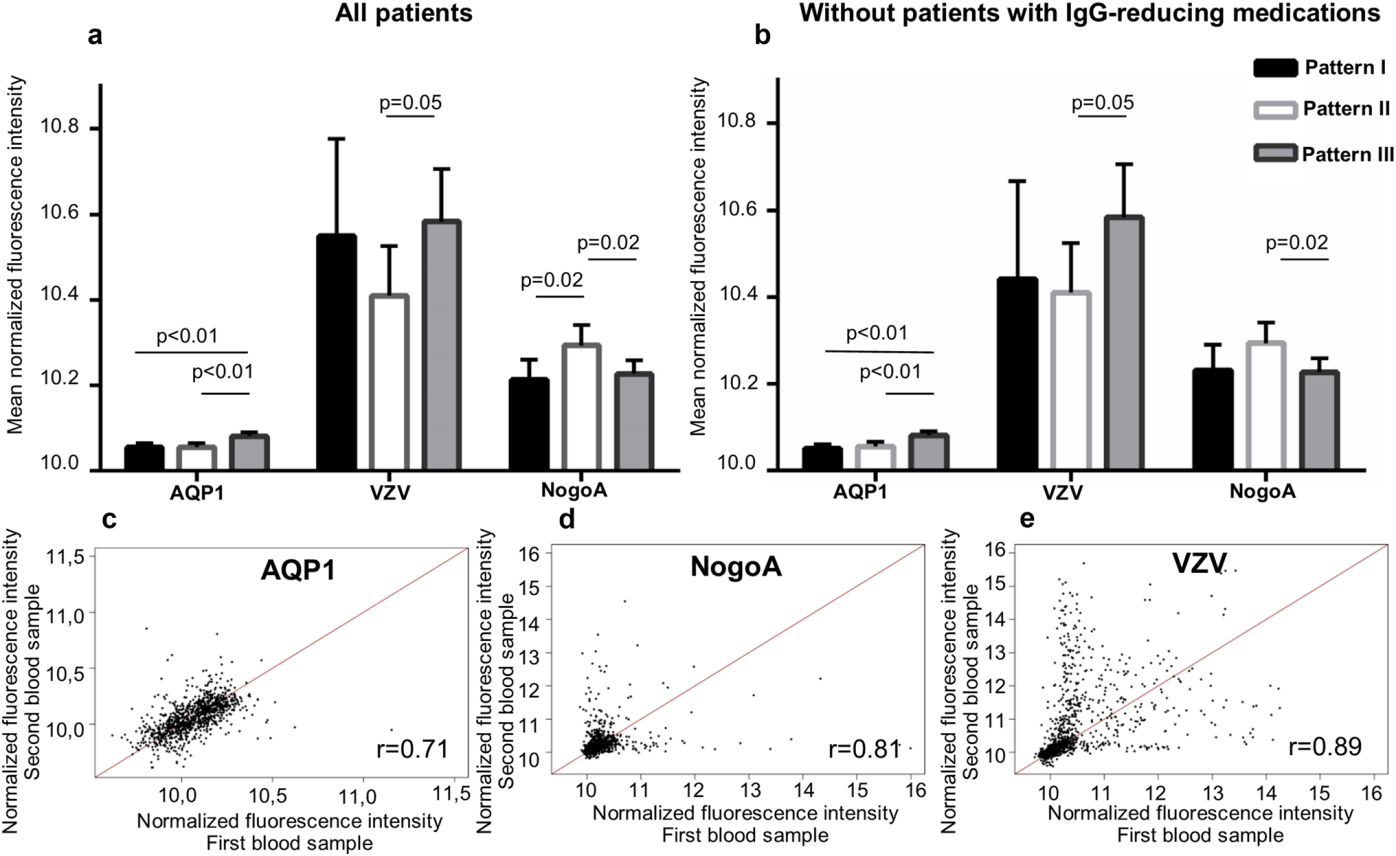
Antibody binding reactivities in three histopathological patterns for AQP1, VZV and Nogo-A peptides, and correlation of these antibody reactivities among first and second blood sampling. **a** Antibody reactivities shown as mean normalized fluorescence intensity units for AQP1, VZV and Nogo-A peptide groups without correction for therapies given before blood sampling (*p* values of Global test are given); **b** antibody reactivities shown in mean normalized fluorescence intensity units for selected peptide groups after correction for therapies given before blood sampling: three patients with PLEX/AI and/or HDCS therapies that likely reduce IgG levels in serum were excluded from analyses (*p* values of Global test are given); **c**–**e** pairwise comparison of peptide reactivities between first and second blood sample for AQP1, Nogo-A and VZV peptide groups indicated by the average correlation index. Reactivities are shown as mean normalized fluorescence intensity units. *AQP1* aquaporin 1, *VZV* varicella zoster virus, *PLEX* therapeutic plasma exchange, *IA* immunoadsorption, *HDCS* high dose of corticosteroids

The differential antibody reactivities to AQP1, VZV and Nogo-A peptides comparing immunopathological patterns are visualized in Fig. 4a.

### Antibody reactivities are not substantially influenced by previous medications

As a next step, we investigated possible medication effects on IgG reactivities. We excluded three patients with therapies that probably reduce IgG levels in serum (see method section, PLEX/IA and HDCS). Our analyses still showed no single differentially bound peptides among patterns I-III. Analysis of groups of peptides further supported higher reactivities of anti-AQP1 and anti-VZV antibodies in pattern III and anti-Nogo-A antibodies in pattern II patients (Fig. 4b). We then also excluded nine patients with medications possibly reducing IgG levels in serum (rituximab, cyclophosphamide, azathioprine, mitoxantrone and teriflunomide), with no effect on single peptide reactivities. Differences in the antibody binding to AQP1 peptides did not change. The binding reactivity to VZV and Nogo-A peptide groups was still higher in patterns III and II, but it no longer reached statistical significance.

### Antibody reactivities in follow-up blood samples

For a subset of 30 patients, 1-year follow-up serum samples were available for analysis (pattern I *n* = 9, pattern II *n* = 10; pattern III *n* = 11), with a median time interval between the first and second sample of 12.0 months (range 4.8–20.0 months). About half of the patients (*n* = 14) received MS-specific therapies at this time (rituximab *n* = 3, natalizumab *n* = 3, interferons *n* = 3, copaxone *n* = 2, cyclophosphamide *n* = 2, fingolimod *n* = 1). The disease course changed from CIS to RR MS in one pattern I patient. The average correlation index of antibody reactivities between first and second MS blood samples was *r* = 0.8, suggesting that antibody reactivities are fairly stable over a time period of 1 year. AQP1, VZV and NogoA peptide groups showed a high average correlation between the first and second blood samples (Fig. 4c–e). The comparison of peptide groups in follow-up blood samples was hampered by low sample numbers: The reactivity against AQP1 peptides was no longer higher in pattern III than in pattern II patients (*p* = 0.07), also not compared to pattern I patients (*p* = 0.86). The most stable antibody reactivities were found for VZV peptides (*r* = 0.89, Fig. 4c), which still showed highest reactivities in pattern III patients, however without statistical significant differences compared to pattern II patients (*p* = 0.76). For Nogo-A peptides the antibody binding reactivities in pattern II patients were no longer statistically significant higher than in pattern III patients (*p* = 0.07), also not compared to pattern I patients (*p* = 0.30).

### Baló’s concentric sclerosis patients show a distinct antibody signature

In a final step, we took another stratification approach and compared patients with histological and/or MRI evidence of concentric demyelination, indicative of Baló’s concentric sclerosis (*n* = 8) and compared these with MS patients lacking such evidence (*n* = 53; demographic and basic clinical data of cohorts shown in Table 1). Eight pattern III patients and none of the patterns I and II patients fulfilled criteria for Baló’s concentric sclerosis. Thus, Baló’s concentric sclerosis patients comprise a subgroup of pattern III patients.

**Fig. 5:**
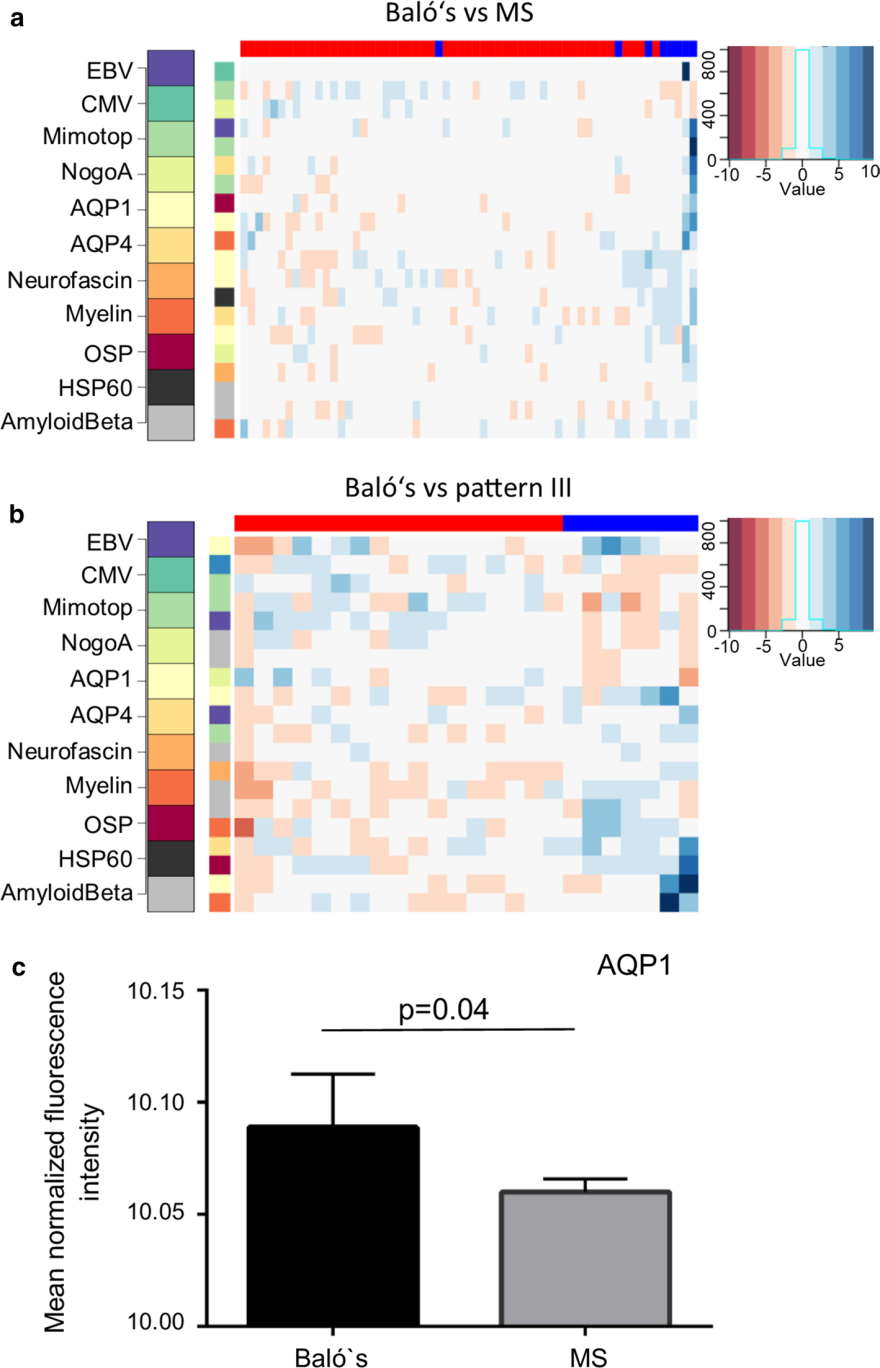
Patients with Baló’s concentric sclerosis show a distinct antibody signature and higher antibody reactivities to AQP1 peptides compared to non-Baló’s patients. **a** A heat map comparing Baló’s concentric sclerosis patients [blue] vs all other multiple sclerosis patients [red] is shown. The top 20 peptides from pairwise subgroup comparisons are shown as rows (see key for peptides). Patients from the two subgroups are shown as columns [red for Baló’s patients, blue for non-Baló’s patients]. The color (see key for z score) indicates the intensity of the peptide reactivities standardized to healthy controls, with blue indicating an upregulation and red a downregulation of peptide reactivities, given in standard deviations. The heat map shows a clustering of Baló’s patients, suggesting a different antibody signature in this patient subgroup. **b** A heat map comparing Baló’s concentric sclerosis patients [blue] vs multiple sclerosis patients of pattern III [red] is shown. The top 20 peptides from pairwise subgroup comparisons are shown as rows (see key for peptides). Patients from the two subgroups are shown as columns [red for Baló’s patients, blue for non-Baló’s patients]. The color (see key for z score) indicates the intensity of the peptide reactivities standardized to healthy controls, with blue indicating an upregulation and red a downregulation of peptide reactivities, given in standard deviations. The heat map shows a clustering of Baló’s patients, suggesting a different antibody signature in this patient subgroup. **c** Antibody reactivities shown as mean normalized fluorescence intensity units for AQP1 peptides show a higher reactivity in patients with Baló’s concentric sclerosis compared to non-Baló´s patients (*p* value of Global test is given). *EBV* Epstein–Barr virus, *VZV* varicella-zoster virus, *CMV* cytomegalovirus, *AQP1* aquaporin 1, *AQP4* aquaporin 4, *MOG* myelin oligodendrocyte glycoprotein

Analyzing differences in single peptide reactivities, none of the peptides showed significantly different binding in Baló’s patients after adjustment for multiple testing. Next, instead of analyzing single peptides, twenty peptides with the most prominent differences between Baló and non-Baló patients that included HSP60, AQP1, AQP4, CMV, myelin and oligodendrocyte peptides, as well as mimotopes, were selected for a heat map, although the single peptides did not show significantly different binding after adjustment for multiple testing. This heat map showed a clustering of Baló’s patients, suggesting a different antibody signature in this patient subgroup compared to non-Baló patients (Fig. 5a). Also, comparison of heat maps of the twenty peptides with the most prominent differences between Baló patients and non-Baló pattern III patients showed a clustering of patient subgroups (Fig. 5b).

Comparing binding reactivities to different peptide groups, Baló’s concentric sclerosis patients showed higher binding for AQP1 peptides (*p* = 0.04 Fig. 5c), as was shown beforehand for pattern III versus patterns I and II patients.

Accordingly, we then analyzed a potential AQP1 loss in brain sections. Immunohistochemical stainings revealed areas of dystrophic astrocytes and simultaneous loss of both AQP1 and AQP4 in three out of eight patients with Baló’s concentric sclerosis (see Fig. 2d–f and Supplementary Fig. 1, online resource). These lesions did not feature any other NMO-typical characteristics [10, 65]. None of the non-Baló’s patients showed a loss of AQP1 or AQP4.

## Discussion

We analyzed antibody reactivities to CNS and viral antigens potentially associated with autoimmune inflammatory demyelinating diseases, as well as mimotopes in different subgroups of MS patients grouped according to their histopathologically determined pattern of demyelination (patterns I–III). Furthermore, we compared patients with Baló’s concentric sclerosis to patients showing inflammatory demyelinating lesions, but with no evidence of concentric demyelination. Our goal was to identify peptide antibody reactivities that distinguish MS subgroups and enhance our understanding of disease mechanisms.

Although it is currently unproven whether the different histopathological patterns I–III reflect specific etiological differences as opposed to different mechanisms of tissue injury, evidence from our own work shows that these patterns can be clearly discerned on neuropathological grounds, are intraindividually stable, and may thus be associated with clinical outcome and therapy response, as has already been shown for apheresis treatment [75].

Here we report the largest study of a systematic antibody screening in patients with histologically verified immunopathological patterns of MS, as well as in patients with Baló’s disease.

When investigating potential biomarkers, it is important to take into account the stability of antibody reactivities over time. Thus, we analyzed a second serum sample from a representative subgroup of our patients (including patterns I, II and III patients) after 1 year. Analysis showed a good stability of antibody reactivities with a high average correlation index of antibody reactivities between first and second MS blood samples of *r* = 0.8.

In our study, patients with pattern III lesions (including those with Baló’s concentric sclerosis) showed a higher binding to peptides of the water channel AQP1. AQP1 is widely expressed in diverse tissues, and its expression was first described on erythrocytes [3]. Within the CNS it is expressed on astrocytes and choroid plexus cells [69]. AQP1 immunoreactivity is predominantly localized in the white matter and less in the gray matter, subpial, and subependymal regions as compared to AQP4 [54]. A distinct lesion location of pattern III lesions could not be observed (data not shown). Also, the subgroup of pattern III patients with Baló’s concentric sclerosis showed higher AQP1 reactivities. Antibodies directed against AQP1 (with or without additional AQP4-antibodies) have been found before in patients with NMOSD and MS [40, 78, 79], but results could not be confirmed when using a cell-based assay [31]. Also in our patients, no AQP1 antibodies were detected with a cell-based assay. Thus, it seems unlikely that the AQP1 antibodies recognize conformational, extracellular epitopes and are pathogenic antibodies that cause disease, as shown for AQP4 antibodies and NMOSD [4, 38]. However, antibodies may occur by epitope spreading due to the liberation of astrocytic antigens [15, 23, 77]. A sensitization due to recognition of peripheral AQP1 antigen also cannot be excluded. The hallmark of pattern III lesions is inflammatory demyelination associated with oligodendroglial apoptosis and a selective loss of the myelin proteins MAG and CNP [42]. In Baló’s concentric sclerosis a loss of AQP4 expression on hypertrophic astrocytes has been described [47]. Loss of AQP1 expression was detected in some demyelinated lesions from NMO patients [53]. In our study, four out of seven patients with the highest reactivities to AQP1 were classified as Baló patients. In a subset of Baló patients, focal areas with an AQP1 and AQP4 loss were detected histologically, indicating astrocytic damage that may have led to epitope spreading with antibodies that recognize AQP1 peptides.

How could this astrocytic damage associated with AQP1 and AQP4 loss be related to oligodendrocyte apoptosis and loss of MAG and CNP as is observed in pattern III patients? Animal models of NMOSD showed that oligodendroglial death rapidly followed astrocytic demise, astrocytic death led to secondary demyelination, and oligodendrocyte death preceded the infiltration of immune cells [82]. Factors triggered by dying astrocytes such as an increase in extracellular glutamate or alterations in the tissue microenvironment due to astrocyte loss may cause oligodendroglial death. Thus, astrocytic damage in pattern III patients may cause oligodendrocyte metabolic changes which in turn result in MAG and CNP loss as well as oligodendrocyte apoptosis, all of which are also features of NMOSD lesions [10].

There is an important difference between NMOSD and pattern III lesions: in NMOSD, IgG and complement deposits are found, but these humoral factors are absent in pattern III lesions. Astrocyte dysfunction may also be caused by other mechanisms such as lipopolysaccharide (LPS) injection, which also results in oligodendroglial cell death [70]. Interestingly, astrocytic changes were described beforehand in MS pattern III lesions and showed similarities to those in NMOSD and LPS-induced lesions [70]. The authors reported that in NMO lesions, astrocytes were widely destroyed, leading to pronounced loss of astrocytes. However, in MS, astrocyte pathology mainly affected the cell processes; damage to astrocytes was minor [70]. In summary, these observations point to the importance of astrocyte functionality for oligodendroglial survival. As in NMOSD, it is possible that astrocytic changes are involved in the pathogenesis of myelin and oligodendrocyte damage in pattern III lesions [70, 82].

In our cohort, a higher VZV binding reactivity was also evident in pattern III compared to pattern II patients. VZV infection has repeatedly been suggested as a trigger of MS [71]. Reappearance of VZV DNA in peripheral blood mononuclear cells with an acute MS relapse has been described [71]. Moreover, the MRZ reaction (CSF antibodies against VZV, measles and rubella) is positive in the majority of MS patients [62], and has a prognostic value for conversion from CIS to a RR disease course [7]. The MRZ reaction was investigated in 267 patients with definite multiple sclerosis, and antibodies to VZV were found in 55% [62]. The pathophysiological significance of this finding is still not clear. Also, the question of whether an increased risk of MS is associated with varicella and zoster infections remains under discussion [45, 46, 66]. Pattern III lesions are characterized by an oligodendrocyte apoptosis [42], which together with neurons are target cells for VZV infection within the CNS [33, 55]. However, we did not find clinical or histological evidence of VZV infection in pattern III lesions. Alternatively, it is possible that instead of causing CNS infection, latent infection with VZV could trigger peripheral immune system activation and thus foster CNS demyelination in a subset of MS patients.

Antibodies to CNS antigens may also be beneficial by promoting tissue repair. This has been shown for anti-Nogo-A antibodies. Nogo-A is a known inhibitor of neurite outgrowth, expressed in the CNS on oligodendrocytes, myelin and certain neurons. Studying spinal cord injury and stroke in animal models, it was shown that the neutralization of Nogo-A with antibodies facilitates axonal growth and functional recovery [8, 81]. Using the MS mouse model EAE, blocking Nogo-A receptors ameliorated the disease course, boosted functional recovery, and increased axonal sprouting and remyelination [28, 30]. Antibodies are thought to neutralize inhibitory effects of Nogo-A and thus to activate axonal and myelin regeneration. Moreover, Nogo-A antibodies could modulate autoimmune inflammation by switching T cells to an anti-inflammatory Th2 phenotype [19, 30], although it is debated whether this effect is due to targeting Nogo-B, which is widely expressed on immune cells, rather than Nogo-A [74].

In our study patients with pattern II MS lesions were characterized by higher antibody reactivities to Nogo-A peptides. Prior studies investigated autoantibody responses to the large N-terminal domain of Nogo-A and showed that Nogo-A IgM antibodies were significantly increased in patients with MS and other neurological diseases, but not in systemic autoimmune diseases and healthy controls [64]. An intrathecal production of Nogo-A IgG antibodies has also been shown in MS, and antibodies were higher in younger patients and in relapsing–remitting MS as compared to chronic progressive MS [64]. No significant differences regarding the age at the time of blood sampling or the disease course were found when comparing patterns I–III, so that these factors do not seem to influence anti-Nogo-A antibody levels in our cohorts. Another study showed that a substantial proportion of MS patients and also patients with other neurological diseases as well as healthy controls exhibited serum IgG autoantibodies against the common N66 region of Nogo-A [58]. To our knowledge, no studies exist that investigated in MS patients whether anti-Nogo-A antibodies are indeed neutralizing antibodies, although Reindl et al. showed in 2002 that at least the antibodies recognized Nogo-A in brain extracts, oligodendrocytes and cells expressing human Nogo-A [64]. Thus, the biological significance of anti-Nogo-A antibodies remains elusive. In previous investigations we found less acute axonal damage in pattern II than in pattern I and III lesions, which fits well with potential axon protective functions of anti-Nogo-A antibodies [26]. However, these observations are preliminary and whether anti-Nogo-A antibodies are indeed associated with less axonal damage and better clinical outcome has to be verified in a larger cohort.

In a second approach, we compared patients with Baló´s concentric sclerosis with all other biopsied MS patients. The reason for the peculiar demyelination pattern in Baló lesions is unclear. It has been suggested that the concentric patterning is caused by demyelination through histotoxic hypoxia alternating with areas protected by tissue preconditioning through upregulation of hypoxia inducible factor 1α (HIF1α) and heat shock protein 70 (HSP70) [73]. Heatmaps of the 20 peptides with the most pronounced differences from pairwise group comparisons showed differences between Baló’s concentric sclerosis patients and the non-Baló MS cohort. Peptides included HSP60 and AQP1 as well as AQP4. In addition, histology revealed astrocytic damage in a subset of Baló patients. These findings support the concept that Baló’s concentric sclerosis could represent a unique pathological entity, where pathological changes with a histotoxic hypoxia and an upregulation of heat shock proteins, as well as astrocytic affection, may play a crucial role in disease development.

Our results are based on a cohort of patients with biopsy-proven inflammatory demyelinating disease and thus carry potential biases. However, published evidence suggests that findings from biopsied patients can be extrapolated to prototypic MS. Despite atypical clinical presentations (i.e., tumefactive lesions on MRI; older age), a prior study of biopsy cases (*n* = 91) reported that 90% of patients developed clinically definite or probable MS during a median follow-up of 4.4 years. The clinical course and disability during follow-up in the biopsied cohort were indistinguishable from the non-biopsied prevalence cohort matched for disease duration, age, and sex (*n* > 200) [59]. In addition, our clinical-radiographic study of 168 patients with tumefactive biopsied inflammatory demyelinating lesions showed that 70% developed definite MS at last follow-up, with 83% of patients presenting with multiple lesions and 55% fulfilling Barkhof radiographic criteria for MS at the time of last MRI [43]. We thus conclude that patients who presented with atypical clinical symptoms that led to biopsy nevertheless comprise a representative and informative cohort of MS patients.

The short disease duration of patients may be regarded as limitation of our study. However, as mentioned above, most patients do develop typical MS during follow-up. We consider early disease stages to be optimal for investigating the initial mechanisms that lead to lesion formation, and particularly in these stages it is possible to observe inter-individual lesion heterogeneity. Antigen microarrays have been successfully used in numerous studies on demyelinating autoimmune diseases, including MS for analysis of disease-specific antibodies and distinction of patient subgroups [51, 60, 84]. Still, the linearity of peptides is clearly an important limitation of our study. Post-transcriptional conformational changes in the proteins are important for antibody recognition and binding. Autologous proteins may become immunogenic if they are structurally modified post-translationally by transglutamination, deamidation, glycosylation, oxidation, nitration or proteolytic cleavage [1]. We cannot exclude the possibility that we missed antibody reactivities that only recognize conformational and post-translationally modified epitopes. Also, we cannot exclude multispecificity of antibody reactivities. In addition, a relatively small number of participants in each group may be regarded as a limitation of the study. We could not increase patient numbers in the study due to the limited number of biopsied MS patients. Nevertheless, this study is the largest systemic antibody screening in patients with detailed histological characterization. We ruled out that prior immunosuppressive/immunomodulatory treatment influenced our results.

Although antibody signatures do not allow allocation of single patients to patterns I–III or Baló’s concentric sclerosis, our findings explain some of the heterogeneity of MS lesion pathology by showing characteristic antibody reactivities that may help understand the pathogenic mechanisms of lesion development. The present data provide further evidence that Baló’s concentric sclerosis may be a distinct disease entity. Better identification and characterization of MS patient subgroups, based on their assumed pathogenesis, will allow more personalized MS patient care.

## Electronic supplementary material

Below is the link to the electronic supplementary material.

Supplementary Figure 1. Astrocytic stainings in different immunopathological patterns and in Baló's concentric sclerosis. Pattern I lesions (a–c) and pattern II lesions (d–f) are characterized by a reactive gliosis (GFAP staining) with no AQP4 or AQP1 loss. Also, pattern III lesions without histological and/or MRI evidences of Baló’s concentric sclerosis show such a reactive astrogliosis with preserved AQP4 and AQP1 expression (g–i). In contrast, a subset of pattern III patients with histological and/or MRI features of Baló’s concentric sclerosis show dystrophic astrocytes with reduced numbers of astrocytes (j) as well as a loss of both AQP4 (k) and AQP1 (l) expression. (TIF 11082 kb)

Supplementary file2 (DOCX 32 kb)

Supplementary file3 (DOCX 12 kb)

Supplementary file4 (DOCX 13 kb)

Supplementary file5 (DOCX 43 kb)
