# Supplementary figures and images for: Antibody signatures in patients with histopathologically defined multiple sclerosis patterns
Source: Acta Neuropathol. 2020 Jan 16;139(3):547–64. doi: 10.1007/s00401-019-02120-x (PMC7035238; doi:10.1007/s00401-019-02120-x)

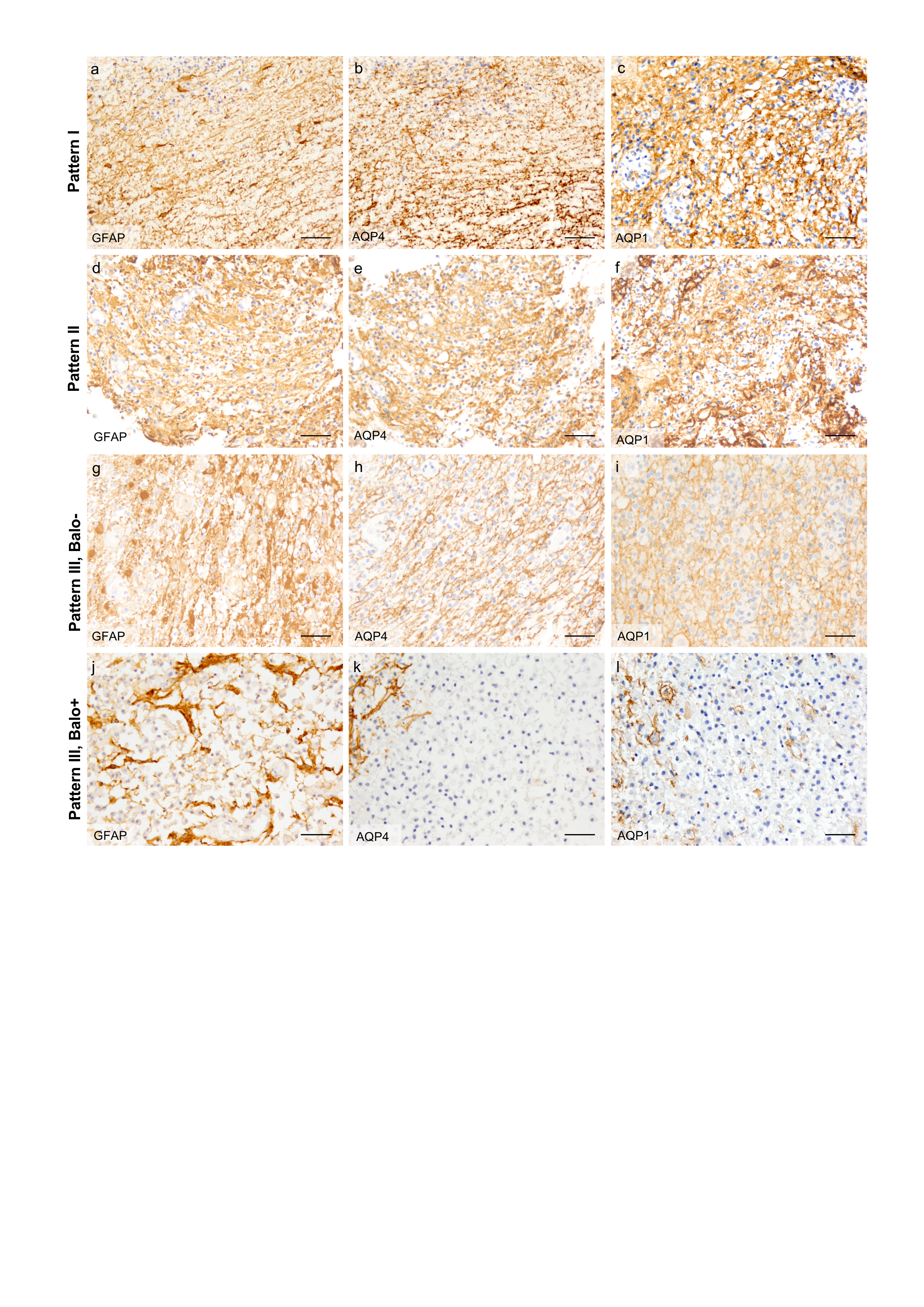

Supplement: Supplementary file 1 — Supplementary Figure 1. Astrocytic stainings in different immunopathological patterns and in Baló's concentric sclerosis. Pattern I lesions (a–c) and pattern II lesions (d–f) are characterized by a reactive gliosis (GFAP staining) with no AQP4 or AQP1 loss. Also, pattern III lesions without histological and/or MRI evidences of Baló’s concentric sclerosis show such a reactive astrogliosis with preserved AQP4 and AQP1 expression (g–i). In contrast, a subset of pattern III patients with histological and/or MRI features of Baló’s concentric sclerosis show dystrophic astrocytes with reduced numbers of astrocytes (j) as well as a loss of both AQP4 (k) and AQP1 (l) expression. (TIF 11082 kb) [file 401_2019_2120_MOESM1_ESM.tif]
