# Supplementary material for: Antibody signatures in patients with histopathologically defined multiple sclerosis patterns
Source: Acta Neuropathol. 2020 Jan 16;139(3):547–64. doi: 10.1007/s00401-019-02120-x (PMC7035238; doi:10.1007/s00401-019-02120-x)
Supplement: Supplementary file 2 — Supplementary file2 (DOCX 32 kb) [file 401_2019_2120_MOESM2_ESM.docx]

**Supplementary Table 1:**

**This table was published beforehand in Metz et al., Neurol Neuroimmunol Neuroinflamm 2016.**

Summary content of Selection Microarray

| **Peptide group** | **Number of peptides included in Selection Microarray** |
| --- | --- |
| Differentially bound / high reactivity >4096 arbitrary units | 439 |
| EBV-EBNA1 | 62 |
| Viral and >1024 arbitrary units | 23 |
| AQP4, aa 46-78, 129-163, 198-239 | 50 |
| AQP1, aa 29-56, 108-144, 176-192, 193-215 | 27 |
| MOG, aa 27-57, 88-134 | 26 |
| Kir4.1, aa 83-120 | 13 |
| Peptides published by Quintana et al. (Quintana *et al.,* 2008) | 23 |
| Control peptides | 39 |
| Sum | 702 |

**Complete list of peptides included in Selection Microarray**

| **Annotation Protein** | **Group** | **Peptide** |
| --- | --- | --- |
| aBCrystallin | Differentially bound | ERQDEHGFISREFHR |
| aBCrystallin | Differentially bound | PFHSPSRLFDQFFGE |
| AmyloidBeta | Differentially bound | EADDDEDDEDGDEVE |
| AmyloidBeta | Differentially bound | EDDEDGDEVEEEAEE |
| AmyloidBeta | Differentially bound | EEAEEPYEEATERTT |
| AmyloidBeta | Differentially bound | EEEVAEVEEEEADDD |
| AmyloidBeta | Differentially bound | ETGPCRAMISRWYFD |
| AmyloidBeta | Differentially bound | EVEEEEADDDEDDED |
| AmyloidBeta | Differentially bound | GDEVEEEAEEPYEEA |
| AmyloidBeta | Differentially bound | IHHGVVEVDAAVTPE |
| AmyloidBeta | Differentially bound | ISYGNDALMPSLTET |
| AmyloidBeta | Differentially bound | KHRERMSQVMREWEE |
| AmyloidBeta | Differentially bound | QITNVVEANQPVTIQ |
| AmyloidBeta | Differentially bound | SDVWWGGADTDYADG |
| AmyloidBeta | Differentially bound | VYPELQITNVVEANQ |
| AmyloidBeta | Differentially bound | YGGCGGNRNNFDTEE |
| AN-2 | Differentially bound | AGCRLEEEEYEDDAY |
| AN-2 | Differentially bound | AGNILYEHEMPPEPF |
| AN-2 | Differentially bound | AVPGQGPPPGGQPDP |
| AN-2 | Differentially bound | EEEEYEDDAYGHYEA |
| AN-2 | Differentially bound | EEPEAAYRLIQGPQY |
| AN-2 | Differentially bound | GPPPGGQPDPELLQF |
| AN-2 | Differentially bound | RVANGSSLVVPQGGQ |
| AN-2 | Differentially bound | SSGLPVERRDQPGEP |
| AN-2 | Differentially bound | STGLRLAQGSAMPIL |
| AN-2 | Differentially bound | VERRDQPGEPATEFS |
| AN-2 | Differentially bound | VVDPDSAPGEIEYEV |
| ApoE | Differentially bound | CQAKVEQAVETEPEP |
| ApolipoproteinE | Differentially bound | EQAVETEPEPELRQQ |
| ApolipoproteinE | Differentially bound | MEDVCGRLVQYRGEV |
| AQP4 | Differentially bound | AVLAGGLYEYVFCPD |
| AQP4 | Differentially bound | GPIIGAVLAGGLYEY |
| AQP4.129-163 | Differentially bound | VTPPSVVGGLGVTMV |
| AQP4.129-163 | Differentially bound | VVGGLGVTMVHGNLT |
| bax | Differentially bound | QDQGGWDGLLSYFGT |
| bcl-2 | Differentially bound | AFFEFGGVMCVESVN |
| bcl-2 | Differentially bound | GGVMCVESVNREMSP |
| bcl-2 | Differentially bound | HLHTWIQDNGGWDAF |
| bcl-2 | Differentially bound | QAGDDFSRRYRRDFA |
| BDNF | Differentially bound | EEYKNYLDAANMSMR |
| BDNF | Differentially bound | LLFLLEEYKNYLDAA |
| Beta-Synuclein | Differentially bound | DPPQEEYQEYEPEAG |
| Beta-Synuclein | Differentially bound | GESYEDPPQEEYQEY |
| Beta-Synuclein | Differentially bound | REEFPTDLKPEEVAQ |
| Beta-Synuclein | Differentially bound | TGLVKREEFPTDLKP |
| CHI3L1 | Differentially bound | EAGTLAYYEICDFLR |
| CHI3L1 | Differentially bound | HIDTWEWNDVTLYGM |
| CHI3L1 | Differentially bound | MVWALDLDDFQGSFC |
| CHI3L1 | Differentially bound | QLAGAMVWALDLDDF |
| Contactin-2 | Differentially bound | AETAQVLGLTPWMDY |
| Contactin-2 | Differentially bound | AETYALVGQQVTLEC |
| Contactin-2 | Differentially bound | DDFPIDFDKPGGHYR |
| Contactin-2 | Differentially bound | HLTGKNWIEIPVPED |
| Contactin-2 | Differentially bound | KAVVLWSKGTEILVN |
| Contactin-2 | Differentially bound | LGNYSCLATSHMDFS |
| Contactin-2 | Differentially bound | LVGQQVTLECFAFGN |
| Contactin-2 | Differentially bound | PGLSYRWLLNEFPNF |
| Contactin-2 | Differentially bound | QIPSVSFEDEGTYEC |
| Contactin-2 | Differentially bound | QQDMNGILLGYEIRY |
| Contactin-2 | Differentially bound | SALGSQTTFGPVFED |
| Contactin-2 | Differentially bound | SANATTMKPPPRRPP |
| Contactin-2 | Differentially bound | SFEDEGTYECEAENS |
| CYP27B1 | Differentially bound | CRQRACGLLTAEGEE |
| CYP27B1 | Differentially bound | HLVPGPWGRLCRDWD |
| CYP27B1 | Differentially bound | PWGRLCRDWDQMFAF |
| EAAT2 | Differentially bound | LNETVTEVPEETKMV |
| FerritinHeavyChain | Differentially bound | DPHLCDFIETHYLNE |
| GFAP | Differentially bound | DLERKIESLEEEIRF |
| GFAP | Differentially bound | EWYRSKFADLTDAAA |
| GFAP | Differentially bound | KFADLTDAAARNAEL |
| Glutaminsynthetase | Differentially bound | GYFEDRRPSANCDPF |
| Glutaminsynthetase | Differentially bound | SDMYLVPAAMFRDPF |
| HIF-1alpha | Differentially bound | CLVLICEPIPHPSNI |
| HIF-1alpha | Differentially bound | CTGHIHVYDTNSNQP |
| HIF-1alpha | Differentially bound | DAARSRRSKESEVFY |
| HIF-1alpha | Differentially bound | DSDMVNEFKLELVEK |
| HIF-1alpha | Differentially bound | EPNPESLELSFTMPQ |
| HIF-1alpha | Differentially bound | GSGSGSGMEGAGGAN |
| HIF-1alpha | Differentially bound | GYEPEELLGRSIYEY |
| HIF-1alpha | Differentially bound | IQDQTPSPSDGSTRQ |
| HIF-1alpha | Differentially bound | LEMLAPYIPMDDDFQ |
| HIF-1alpha | Differentially bound | PYIPMDDDFQLRSFD |
| HIF-1alpha | Differentially bound | QTQIQEPTANATTTT |
| HIF-1alpha | Differentially bound | SLELSFTMPQIQDQT |
| HIF-1alpha | Differentially bound | SRHSLDMKFSYCDER |
| HIF-1alpha | Differentially bound | SSPEPNSPSEYCFYV |
| HSP60 | Differentially bound | DVTTSEYEKEKLNER |
| HSP60 | Differentially bound | IDPTKVVRTALLDAA |
| HSP60 | Differentially bound | IEGMKFDRGYISPYF |
| HSP60 | Differentially bound | LEDVQPHDLGKVGEV |
| HSP60 | Differentially bound | PHDLGKVGEVIVTKD |
| HSP70 | Differentially bound | AAIAYGLDRTGKGER |
| HSP70 | Differentially bound | FELSGIPPAPRGVPQ |
| HSP70 | Differentially bound | GGSGSGPTIEEVDGS |
| HSP70 | Differentially bound | GLDRTGKGERNVLIF |
| HSP70 | Differentially bound | IAGLNVLRIINEPTA |
| HSP70 | Differentially bound | QRQATKDAGVIAGLN |
| HSP70 | Differentially bound | TFDVSILTIDDGIFE |
| IFNAR1 | Differentially bound | APKQSGNTPVIQDYP |
| IFNAR1 | Differentially bound | CFIIENISTIATVEE |
| IFNAR1 | Differentially bound | GNTPVIQDYPLIYEI |
| IFNAR1 | Differentially bound | NISTIATVEETNQTD |
| Kir4.1 | Differentially bound | QTTIGYGFRYISEEC |
| Kir4.1 | Differentially bound | SGTIGYGFRYISEEC |
| Mimotop1 | Differentially bound | LMRWSANEHDATLDQ |
| Mimotop10 | Differentially bound | RPGNQEPFVRWRDYW |
| Mimotop100 | Differentially bound | SPRDVIWFMADDRPP |
| Mimotop101 | Differentially bound | FFLKFELMHERGEPM |
| Mimotop102 | Differentially bound | AMGPMSDPSWIWSLN |
| Mimotop103 | Differentially bound | SPGGMVNQVHHGDFA |
| Mimotop104 | Differentially bound | HMQSSEHAQRRGDYP |
| Mimotop105 | Differentially bound | NAEHNHSEQSAPQMG |
| Mimotop106 | Differentially bound | TRVHNLTLQVHQSLN |
| Mimotop107 | Differentially bound | NVAINKTRRAAGANL |
| Mimotop108 | Differentially bound | RQSPWPFYTRRVDFP |
| Mimotop109 | Differentially bound | TDEMHWIHHELFPNA |
| Mimotop11 | Differentially bound | TSHIVARDWGTFEYY |
| Mimotop110 | Differentially bound | YIHVWGKPSPQASLN |
| Mimotop111 | Differentially bound | AGIKMHRGTSEHDYP |
| Mimotop112 | Differentially bound | DNDEDEAPDAADMVW |
| Mimotop113 | Differentially bound | WARLPSAPPTAFQER |
| Mimotop114 | Differentially bound | VVQRSDWAPQPQYNE |
| Mimotop115 | Differentially bound | DNEAQGQGEVYMEMD |
| Mimotop116 | Differentially bound | WKTAPSLWNNMPVQT |
| Mimotop117 | Differentially bound | RLGVRIAHYYEVEPF |
| Mimotop118 | Differentially bound | LIFGSRQYQEGYYYN |
| Mimotop119 | Differentially bound | QYQNENITWDYTPEY |
| Mimotop12 | Differentially bound | QVTFTVWQIYEIDYS |
| Mimotop120 | Differentially bound | NQQTFLNRAVSYQEF |
| Mimotop121 | Differentially bound | AGPQGWHMSEFLWYY |
| Mimotop122 | Differentially bound | EWTIFLYPNQEQPEW |
| Mimotop123 | Differentially bound | EKRYAQTVNMDATNV |
| Mimotop124 | Differentially bound | RVNVHEDAAEAKTIF |
| Mimotop125 | Differentially bound | YQARTALWEIVHHDW |
| Mimotop126 | Differentially bound | HYREDAALDQEQSMV |
| Mimotop127 | Differentially bound | QAHWENTRNEEQHWD |
| Mimotop128 | Differentially bound | IEYQPMVDAEEPDWQ |
| Mimotop129 | Differentially bound | NRQPRYLAIIAHPDN |
| Mimotop13 | Differentially bound | NTVIIPTGWWDVGYY |
| Mimotop130 | Differentially bound | VLFTSDEHQVDMEWF |
| Mimotop131 | Differentially bound | LASVQVDASQNWDEN |
| Mimotop132 | Differentially bound | YNPASPDFEEFDSAV |
| Mimotop133 | Differentially bound | QRFSDGYDNGVRHDF |
| Mimotop134 | Differentially bound | IIMIVISIDYSMNDF |
| Mimotop135 | Differentially bound | HNEPETENHLFYDFV |
| Mimotop136 | Differentially bound | RYNWIYIDNIPELTF |
| Mimotop137 | Differentially bound | DDVVQWMLSIERPNF |
| Mimotop138 | Differentially bound | RWVSQSVNIPTAYEV |
| Mimotop139 | Differentially bound | HSVRWRHDWLHSSNF |
| Mimotop14 | Differentially bound | QLQNNTLEGEQYTEY |
| Mimotop140 | Differentially bound | QDVNHQTFSFNVNNE |
| Mimotop141 | Differentially bound | WNGDHENHMRFNNVD |
| Mimotop142 | Differentially bound | TTPPRDYTVFTNNNA |
| Mimotop143 | Differentially bound | QSVLQVPAWANNTNQ |
| Mimotop144 | Differentially bound | AMVEDNQGPGNNTQN |
| Mimotop145 | Differentially bound | GQSALNPYNYRVNNY |
| Mimotop146 | Differentially bound | DHMAWEEQEGELDFQ |
| Mimotop147 | Differentially bound | IILDVRAIPIYIDEV |
| Mimotop148 | Differentially bound | TSYRDDEMSRPQWEW |
| Mimotop149 | Differentially bound | TDWSYGPMIWRDWFY |
| Mimotop15 | Differentially bound | PSIELRDVRYYLDEF |
| Mimotop150 | Differentially bound | WKRWGQDEDDWGYHD |
| Mimotop151 | Differentially bound | SLFARRSGNERIWYD |
| Mimotop152 | Differentially bound | AVTHGMNYGRMPFDF |
| Mimotop153 | Differentially bound | FQVMQAFPISYEQDY |
| Mimotop154 | Differentially bound | DGMYDIPSISEYYTY |
| Mimotop155 | Differentially bound | HPHRVMQSVFDREWF |
| Mimotop156 | Differentially bound | LDNLPNRWDASDYDD |
| Mimotop157 | Differentially bound | HILMRDFAIIPITET |
| Mimotop158 | Differentially bound | FVNPIIQHDPNYDII |
| Mimotop16 | Differentially bound | PLVRRIISEWFEEVY |
| Mimotop17 | Differentially bound | LNEVDPRQNPPPEED |
| Mimotop18 | Differentially bound | QLIRMPIEENAVTML |
| Mimotop19 | Differentially bound | ESPSEERTINLMVVM |
| Mimotop2 | Differentially bound | KDEFDHIGMEGMDAT |
| Mimotop20 | Differentially bound | NPNPMPQGPPLITAL |
| Mimotop21 | Differentially bound | ADIPTDAAEKWQYPE |
| Mimotop22 | Differentially bound | TPMSSIVYAPTHHQD |
| Mimotop23 | Differentially bound | MPNDFPGYSWMPLPI |
| Mimotop24 | Differentially bound | PPNMQRPEPLPQFEM |
| Mimotop25 | Differentially bound | ANAQHRHVWAHRSEV |
| Mimotop26 | Differentially bound | QAMTYLQVEYTHIVM |
| Mimotop27 | Differentially bound | HHLPPHQMPTPQSLD |
| Mimotop28 | Differentially bound | RHTVDEIMPEDEDIT |
| Mimotop29 | Differentially bound | IGNHQPSTMRTHHDN |
| Mimotop3 | Differentially bound | AQSFTESITNDATNG |
| Mimotop30 | Differentially bound | YVAEWGQLMPDTSMP |
| Mimotop31 | Differentially bound | TGQWNAYGLVMPTQY |
| Mimotop32 | Differentially bound | QMPYQDITAFVEHDF |
| Mimotop33 | Differentially bound | DSRNMPTPHDKQPDP |
| Mimotop34 | Differentially bound | SLVNTNAWVQRDMYF |
| Mimotop35 | Differentially bound | VEALPDAAGLNNSNS |
| Mimotop36 | Differentially bound | EPRFWRNPPRRWYER |
| Mimotop37 | Differentially bound | RQIRGWQVNAEQFFD |
| Mimotop38 | Differentially bound | RLWNTAYSGHEVEYR |
| Mimotop39 | Differentially bound | TFPIPSTVISWNDAA |
| Mimotop4 | Differentially bound | WMMPSVNDYDDNANY |
| Mimotop40 | Differentially bound | LESLEHDAMMEYEQF |
| Mimotop41 | Differentially bound | KVETMRYEDHDWTLY |
| Mimotop42 | Differentially bound | GSPNKHDWGDDRPDN |
| Mimotop43 | Differentially bound | ARHTNSWEWLKYEYD |
| Mimotop44 | Differentially bound | PELPYNNDLEVHYPY |
| Mimotop45 | Differentially bound | DRRRHIVEIVTWERY |
| Mimotop46 | Differentially bound | WEWYTYSKNEWEIFL |
| Mimotop47 | Differentially bound | IETYDGVQDFNYLTW |
| Mimotop48 | Differentially bound | RLEDEQVEQDYFAGW |
| Mimotop49 | Differentially bound | GSTMDIDHEWERVQF |
| Mimotop5 | Differentially bound | ALLDVWNLEFMEHIY |
| Mimotop50 | Differentially bound | YNNSTFVMNQFVDFW |
| Mimotop51 | Differentially bound | TSDTYPNGVIVWPEF |
| Mimotop52 | Differentially bound | HMQIERWYNFGEEDR |
| Mimotop53 | Differentially bound | VVENVNDENDFLSWF |
| Mimotop54 | Differentially bound | QVSGYVDWGLRWFEM |
| Mimotop55 | Differentially bound | DEAPGMFPENDAWYT |
| Mimotop56 | Differentially bound | TDDSGTSHYDVMDYE |
| Mimotop57 | Differentially bound | ARTGRPQQEPVWNYD |
| Mimotop58 | Differentially bound | WNTWPYYERFEHQFN |
| Mimotop59 | Differentially bound | TWYWMYYGASLSSEW |
| Mimotop6 | Differentially bound | VPPEFKPDHFRDHEY |
| Mimotop60 | Differentially bound | GVNNAYFNWNSSDQF |
| Mimotop61 | Differentially bound | GGKPNHPSEVDIYRE |
| Mimotop62 | Differentially bound | AAPANAPNFPEGEYH |
| Mimotop63 | Differentially bound | PYDKLNEIPLIWRPD |
| Mimotop64 | Differentially bound | SNITMIQHPVEVYTQ |
| Mimotop65 | Differentially bound | PDSWSPMDASDDIFI |
| Mimotop66 | Differentially bound | HPDYHLDHNYMLVER |
| Mimotop67 | Differentially bound | TYVSMVYYIQNPIYT |
| Mimotop68 | Differentially bound | SYMQTPRHDEYFAGF |
| Mimotop69 | Differentially bound | WPFGHSKSDMERWER |
| Mimotop7 | Differentially bound | NRNGSYFQDNNGLNY |
| Mimotop70 | Differentially bound | LPLTRMWPPEVKPYQ |
| Mimotop71 | Differentially bound | MREQTLYVMIREPYR |
| Mimotop72 | Differentially bound | LVDSKSQHMWPADVF |
| Mimotop73 | Differentially bound | GSNISVDAVVMRVEP |
| Mimotop74 | Differentially bound | DLAQESVDTVGDPID |
| Mimotop75 | Differentially bound | RVEDPYQWTSASDAF |
| Mimotop76 | Differentially bound | PAWEPHRYVARLFEL |
| Mimotop77 | Differentially bound | SDTDENLMREWPSNE |
| Mimotop78 | Differentially bound | ASTNTHGEHWRSFND |
| Mimotop79 | Differentially bound | SSVNQNSRAMSEDYS |
| Mimotop8 | Differentially bound | VWIWERDIYHPGDPR |
| Mimotop80 | Differentially bound | VEKSRAWISDGNNRF |
| Mimotop81 | Differentially bound | ARENNHPVWGIDVSL |
| Mimotop82 | Differentially bound | NFFGVRVGHNWRPPD |
| Mimotop83 | Differentially bound | VRDHVGSILQNNQSN |
| Mimotop84 | Differentially bound | SERYETYETSRATYN |
| Mimotop85 | Differentially bound | KTFMVYSAHSGNGKY |
| Mimotop86 | Differentially bound | IPVHHMMQELQSYLT |
| Mimotop87 | Differentially bound | KPLEHMNAQTNKQPR |
| Mimotop88 | Differentially bound | RKVHNQLQGKQPNVD |
| Mimotop89 | Differentially bound | AFFNVRKNNQDQKMQ |
| Mimotop9 | Differentially bound | QTQATHNMDGAPENR |
| Mimotop90 | Differentially bound | EPRWFDMEQLNRAEG |
| Mimotop91 | Differentially bound | RSPGWETFMNTGFIH |
| Mimotop92 | Differentially bound | IMLSGQKTMEVADVN |
| Mimotop93 | Differentially bound | ETTTWSNSSEVEFRT |
| Mimotop94 | Differentially bound | NPRGNVHQVAEINNR |
| Mimotop95 | Differentially bound | AAETQNNNQVADNVV |
| Mimotop96 | Differentially bound | QQTGHPDVTQVVHER |
| Mimotop97 | Differentially bound | KYWYEEYQGGHPNNA |
| Mimotop98 | Differentially bound | VDLWGPLMQFWANNT |
| Mimotop99 | Differentially bound | MQGDIISFTTTTNNV |
| Myelin.CNP | Differentially bound | IRILVLDDTNHERER |
| Myelin.CNP | Differentially bound | KLKPGLEKDFLPLYF |
| Myelin.CNP | Differentially bound | LDDTNHERERLEQLF |
| Myelin.CNP | Differentially bound | LEQLFEMADQYQYQV |
| Myelin.CNP | Differentially bound | YKITPGARGAFSEEY |
| Myelin.MAG | Differentially bound | EPSVAFELPSRNVTV |
| Myelin.MAG | Differentially bound | FELPSRNVTVNESER |
| Myelin.MAG | Differentially bound | FSEHSVLDIVNTPNI |
| Myelin.MAG | Differentially bound | PEDDGEYWCVAENQY |
| Myelin.MBP | Differentially bound | ELSRTTSEDNEVFGE |
| Myelin.MOG | Differentially bound | GKNATGMEVGWYRPP |
| Myelin.MOG | Differentially bound | RRLAGQFLEELRNPF |
| Myelin.OSP | Differentially bound | AGDAQAFGENRFYYT |
| Myelin.PLP | Differentially bound | GFYTTGAVRQIFGDY |
| Myelin.PLP | Differentially bound | KLIETYFSKNYQDYE |
| Myelin.PLP | Differentially bound | LLLAEGFYTTGAVRQ |
| Myelin.PLP | Differentially bound | YQDYEYLINVIHAFQ |
| Neurofascin186 | Differentially bound | ATGAVSSTDATAIAA |
| Neurofascin186 | Differentially bound | ERSVRLTWIPGDANN |
| Neurofascin186 | Differentially bound | GDANNSPITDYVVQF |
| Neurofascin186 | Differentially bound | GGRPEEYEGEYQCFA |
| Neurofascin186 | Differentially bound | IDFRSGGRPEEYEGE |
| Neurofascin186 | Differentially bound | LEVKDPTRIYRMPED |
| Neurofascin186 | Differentially bound | LRITNVSEEDSGEYF |
| Neurofascin186 | Differentially bound | NFNKALRITNVSEED |
| Neurofascin186 | Differentially bound | NRTRLDCPFFGSPIP |
| Neurofascin186 | Differentially bound | PEPESVIGYSGEDYP |
| Neurofascin186 | Differentially bound | PKEEDGSFDYSDEDN |
| Neurofascin186 | Differentially bound | RRFRVRQPNLETINL |
| Neurofascin186 | Differentially bound | RVILYNRTRLDCPFF |
| Neurofascin186 | Differentially bound | SDTVQGQLREYRAYY |
| Neurofascin186 | Differentially bound | SPITDYVVQFEEDQF |
| Neurofascin186 | Differentially bound | SSTDATAIAATTEAT |
| Neurofascin186 | Differentially bound | WKHNFGPGTDFVVEY |
| Neurofascin186 | Differentially bound | YVVQFEEDQFQPGVW |
| Neurofilament68kD | Differentially bound | DKEEAEEEEAAEEEE |
| Neurofilament68kD | Differentially bound | EAEEEEKKVEGAGEE |
| Neurofilament68kD | Differentially bound | EEEEAAEEEEAAKEE |
| Neurofilament68kD | Differentially bound | EEEEGGEGEEGEETK |
| Neurofilament68kD | Differentially bound | EEEEKDKEEAEEEEA |
| Neurofilament68kD | Differentially bound | EIRDLRLAAEDATNE |
| Neurofilament68kD | Differentially bound | EPYYSTSYKRRYVET |
| Neurofilament68kD | Differentially bound | GEGEEGEETKEAEEE |
| Neurofilament68kD | Differentially bound | LEETLRNLQARYEEE |
| Neurofilament68kD | Differentially bound | MDEISFLKKVHEEEI |
| Neurofilament68kD | Differentially bound | RNLQARYEEEVLSRE |
| Neurofilament68kD | Differentially bound | RYEEEVLSREDAEGR |
| Neurofilament68kD | Differentially bound | SEEAKEEEEGGEGEE |
| Neurofilament68kD | Differentially bound | SEGEAEEEEKDKEEA |
| Neurofilament68kD | Differentially bound | SGSGMSSFSYEPYYS |
| NOGO-A | Differentially bound | AFKYQFVREPEDEEE |
| NOGO-A | Differentially bound | ANPREEIIVKNKDEE |
| NOGO-A | Differentially bound | DYSEMAKVEQPVPDH |
| NOGO-A | Differentially bound | EDEEEEEEEEEEDED |
| NOGO-A | Differentially bound | EDFPSVLLETAASLP |
| NOGO-A | Differentially bound | EEDEDEDLEELEVLE |
| NOGO-A | Differentially bound | EEEEEEEDEDEDLEE |
| NOGO-A | Differentially bound | FVREPEDEEEEEEEE |
| NOGO-A | Differentially bound | ISAGQEDFPSVLLET |
| NOGO-A | Differentially bound | LFSDDSIPDVPQKQD |
| NOGO-A | Differentially bound | LGNLSTVLPTEGTLQ |
| NOGO-A | Differentially bound | LSAEPAPDFSDYSEM |
| NOGO-A | Differentially bound | PAAGAPLMDFGNDFV |
| NOGO-A | Differentially bound | PDIVMEAPLNSAVPS |
| NOGO-A | Differentially bound | PDLVQEACESELNEV |
| NOGO-A | Differentially bound | PVPDHSELVEDSSPD |
| NOGO-A | Differentially bound | QPGNTISAGQEDFPS |
| NOGO-A | Differentially bound | SAVIVANPREEIIVK |
| NOGO-A | Differentially bound | SDEGHPFRAYLESEV |
| NOGO-A | Differentially bound | SELVEDSSPDSEPVD |
| NOGO-A | Differentially bound | SLSPLSAASFKEHEY |
| NOGO-A | Differentially bound | SSAENMDLKEQPGNT |
| NOGO-A | Differentially bound | VVANMPEGLTPDLVQ |
| Olig1 | Differentially bound | SSSSSTSSTSSTSSS |
| Olig1 | Differentially bound | TSSTSSTSSSSTTAP |
| Olig2 | Differentially bound | AAAAAPLGGGGGGSG |
| S-100b | Differentially bound | TLDNDGDGECDFQEF |
| sTFR | Differentially bound | KLTHDVELNLDYERY |
| Type1interferon | Differentially bound | EEIKQLQQFQKEDAA |
| Type1interferon | Differentially bound | LQQFQKEDAALTIYE |
| Viral.CMV-EnvelopeGlycoproteinC | Differentially bound | AFLERADSVISWDIQ |
| Viral.CMV-EnvelopeGlycoproteinC | Differentially bound | NETIYNTTLKYGDVV |
| Viral.CMV-EnvelopeGlycoproteinC | Differentially bound | SLVELERLANRSSLN |
| Viral.CMV-pp65 | Differentially bound | ATVQGQNLKYQEFFW |
| Viral.CMV-pp65 | Differentially bound | DDDVWTSGSDSDEEL |
| Viral.CMV-pp65 | Differentially bound | DRHDEGAAQGDDDVW |
| Viral.CMV-pp65 | Differentially bound | GAAQGDDDVWTSGSD |
| Viral.CMV-pp65 | Differentially bound | KTPRVTGGGAMAGAS |
| Viral.CMV-pp65 | Differentially bound | LAWTRQQNQWKEPDV |
| Viral.CMV-pp65 | Differentially bound | LKAESTVAPEEDTDE |
| Viral.CMV-pp65 | Differentially bound | MTRGRLKAESTVAPE |
| Viral.CMV-pp65 | Differentially bound | QAIRETVELRQYDPV |
| Viral.CMV-pp65 | Differentially bound | SDEELVTTERKTPRV |
| Viral.CMV-pp65 | Differentially bound | TGGGAMAGASTSAGR |
| Viral.CMV-pp65 | Differentially bound | TSGSDSDEELVTTER |
| Viral.CMV-pp65 | Differentially bound | TVELRQYDPVAALFF |
| Viral.EBV-EBNA1 | Differentially bound | ADDPGEGPSTGPRGQ |
| Viral.EBV-EBNA1 | Differentially bound | AEGDDGDDGDEGGDG |
| Viral.EBV-EBNA1 | Differentially bound | AGAGGGAGAGGGAGG |
| Viral.EBV-EBNA1 | Differentially bound | AGAGGGAGGAGAGGG |
| Viral.EBV-EBNA1 | Differentially bound | AGAGGGAGGAGGAGA |
| Viral.EBV-EBNA1 | Differentially bound | AGAGGGAGGAGGAGG |
| Viral.EBV-EBNA1 | Differentially bound | AGGAGAGGAGGAGAG |
| Viral.EBV-EBNA1 | Differentially bound | AGGAGAGGGAGAGGG |
| Viral.EBV-EBNA1 | Differentially bound | AGGAGAGGGAGGAGG |
| Viral.EBV-EBNA1 | Differentially bound | AGGAGGAGAGGAGAG |
| Viral.EBV-EBNA1 | Differentially bound | AGGAGGAGAGGAGGA |
| Viral.EBV-EBNA1 | Differentially bound | AGGAGGAGGAGAGGA |
| Viral.EBV-EBNA1 | Differentially bound | AGGGAGAGGGAGGAG |
| Viral.EBV-EBNA1 | Differentially bound | AGGGAGGAGGAGAGG |
| Viral.EBV-EBNA1 | Differentially bound | ARGRGRGRGEKRPRS |
| Viral.EBV-EBNA1 | Differentially bound | DEGEEGQEGSGSGSG |
| Viral.EBV-EBNA1 | Differentially bound | DGEPDVPPGAIEQGP |
| Viral.EBV-EBNA1 | Differentially bound | DTSGPEGSGGSGPQR |
| Viral.EBV-EBNA1 | Differentially bound | EGGDGDEGEEGQEGS |
| Viral.EBV-EBNA1 | Differentially bound | GAGAGGAGAGGAGAG |
| Viral.EBV-EBNA1 | Differentially bound | GAGAGGAGAGGAGGA |
| Viral.EBV-EBNA1 | Differentially bound | GAGAGGAGGAGAGGA |
| Viral.EBV-EBNA1 | Differentially bound | GAGAGGGAGGAGAGG |
| Viral.EBV-EBNA1 | Differentially bound | GAGAGGGAGGAGGAG |
| Viral.EBV-EBNA1 | Differentially bound | GAGGAGAGGAGAGGG |
| Viral.EBV-EBNA1 | Differentially bound | GAGGAGAGGAGGAGA |
| Viral.EBV-EBNA1 | Differentially bound | GAGGAGAGGGAGAGG |
| Viral.EBV-EBNA1 | Differentially bound | GAGGAGAGGGAGGAG |
| Viral.EBV-EBNA1 | Differentially bound | GAGGAGGAGAGGAGG |
| Viral.EBV-EBNA1 | Differentially bound | GAGGAGGAGAGGGAG |
| Viral.EBV-EBNA1 | Differentially bound | GAGGAGGAGGAGAGG |
| Viral.EBV-EBNA1 | Differentially bound | GAGGGAGAGGGAGGA |
| Viral.EBV-EBNA1 | Differentially bound | GAGGGAGGAGAGGAG |
| Viral.EBV-EBNA1 | Differentially bound | GAGGGAGGAGAGGGA |
| Viral.EBV-EBNA1 | Differentially bound | GAGGGAGGAGGAGAG |
| Viral.EBV-EBNA1 | Differentially bound | GDDGDEGGDGDEGEE |
| Viral.EBV-EBNA1 | Differentially bound | GGAGAGGAGGAGAGG |
| Viral.EBV-EBNA1 | Differentially bound | GGAGAGGGAGAGGAG |
| Viral.EBV-EBNA1 | Differentially bound | GGAGGAGAGGGAGAG |
| Viral.EBV-EBNA1 | Differentially bound | GGAGGAGAGGGAGGA |
| Viral.EBV-EBNA1 | Differentially bound | GGAGGAGGAGAGGGA |
| Viral.EBV-EBNA1 | Differentially bound | GGGAGAGGAGAGGGG |
| Viral.EBV-EBNA1 | Differentially bound | GGGAGAGGAGGAGGA |
| Viral.EBV-EBNA1 | Differentially bound | GSGSGPRHRDGVRRP |
| Viral.EBV-EBNA1 | Differentially bound | IEQGPADDPGEGPST |
| Viral.EBV-EBNA1 | Differentially bound | PFFHPVGEADYFEYH |
| Viral.EBV-EBNA1 | Differentially bound | PPGRRPFFHPVGEAD |
| Viral.EBV-EBNA1 | Differentially bound | PPRRPPPGRRPFFHP |
| Viral.EBV-EBNA1 | Differentially bound | QEGGPDGEPDVPPGA |
| Viral.EBV-EBNA1 | Differentially bound | RGRGRERARGGSRER |
| Viral.EBV-EBNA1 | Differentially bound | TGAGAGAGGAGAGGA |
| Viral.EBV-EBNA1 | Differentially bound | VEGAAAEGDDGDDGD |
| Viral.EBV-EBNA1 | Differentially bound | VGEADYFEYHQEGGP |
| Viral.EBV-EBNA1 | Differentially bound | VPPGAIEQGPADDPG |
| Viral.EBV-EBNA1 | Differentially bound | YFEYHQEGGPDGEPD |
| Viral.HSV-GlycoproteinC | Differentially bound | AYYPRNPVEFVWFED |
| Viral.HSV-GlycoproteinC | Differentially bound | GSETASTGPTITAGA |
| Viral.HSV-GlycoproteinC | Differentially bound | HQPPPRDPTERQVIE |
| Viral.HSV-GlycoproteinC | Differentially bound | NPVEFVWFEDDHQVF |
| Viral.HSV-GlycoproteinC | Differentially bound | TCTAAAYYPRNPVEF |
| Viral.HSV-GlycoproteinC | Differentially bound | VTGPLPTQRLIIGEV |
| Viral.HSV-GlycoproteinC | Differentially bound | VTPTSTPNPNNVTQN |
| Viral.VZV-GlycoproteinC | Differentially bound | AATRKPDPAVAPTSA |
| Viral.VZV-GlycoproteinC | Differentially bound | APTSAASRKPDPAVA |
| Viral.VZV-GlycoproteinC | Differentially bound | ASRKPDPAVAPTSAA |
| Viral.VZV-GlycoproteinC | Differentially bound | ATRKPDPAVAPTSAA |
| Viral.VZV-GlycoproteinC | Differentially bound | FPPFSAVYTYDASTY |
| Viral.VZV-GlycoproteinC | Differentially bound | GGIQSIPYFHTFIMP |
| Viral.VZV-GlycoproteinC | Differentially bound | LYTSAATRKPDPAVA |
| Viral.VZV-GlycoproteinC | Differentially bound | PHRQLGNALIRMPDL |
| Viral.VZV-GlycoproteinC | Differentially bound | PTSAASRKPDPAVAP |
| Viral.VZV-GlycoproteinC | Differentially bound | PTSAATRKPDPAVAP |
| Viral.VZV-GlycoproteinC | Differentially bound | QLSTESQPTPVSITE |
| Viral.VZV-GlycoproteinC | Differentially bound | RKPDPAANTQHSQPP |
| Viral.VZV-GlycoproteinC | Differentially bound | RKPDPAVAPTSAASR |
| Viral.VZV-GlycoproteinC | Differentially bound | SAASRKPDPAVAPTS |
| Viral.VZV-GlycoproteinC | Differentially bound | SDFSVTILSMDATTE |
| Viral.VZV-GlycoproteinC | Differentially bound | SRKPDPAVAPTSAAS |
| Viral.VZV-GlycoproteinC | Differentially bound | TILSMDATTEGTYIW |
| Viral.VZV-GlycoproteinC | Differentially bound | TRKPDPAVAPTSAAS |
| Viral.VZV-GlycoproteinC | Differentially bound | TSAASRKPDPAVAPT |
| Viral.EBV-EBNA1 | EBV-EBNA1 | AGGAGAGGGGRGRGG |
| Viral.EBV-EBNA1 | EBV-EBNA1 | AGGGGRGRGGSGGRG |
| Viral.EBV-EBNA1 | EBV-EBNA1 | APGPGPQPGPLRESI |
| Viral.EBV-EBNA1 | EBV-EBNA1 | CIGCKGTHGGTGAGA |
| Viral.EBV-EBNA1 | EBV-EBNA1 | DEGTWVAGVFVYGGS |
| Viral.EBV-EBNA1 | EBV-EBNA1 | EGPGTGPGNGLGEKG |
| Viral.EBV-EBNA1 | EBV-EBNA1 | EGPSTGPRGQGDGGR |
| Viral.EBV-EBNA1 | EBV-EBNA1 | EGSGGSGPQRRGGDN |
| Viral.EBV-EBNA1 | EBV-EBNA1 | ENIAEGLRALLARSH |
| Viral.EBV-EBNA1 | EBV-EBNA1 | ERARGGSRERARGRG |
| Viral.EBV-EBNA1 | EBV-EBNA1 | GDGGRRKKGGWFGKH |
| Viral.EBV-EBNA1 | EBV-EBNA1 | GLRALLARSHVERTT |
| Viral.EBV-EBNA1 | EBV-EBNA1 | GPGNGLGEKGDTSGP |
| Viral.EBV-EBNA1 | EBV-EBNA1 | GPRGQGDGGRRKKGG |
| Viral.EBV-EBNA1 | EBV-EBNA1 | GRGRGGSGGRRGRGR |
| Viral.EBV-EBNA1 | EBV-EBNA1 | GRGRGRGGGRPGAPG |
| Viral.EBV-EBNA1 | EBV-EBNA1 | GSGGRRGRGRERARG |
| Viral.EBV-EBNA1 | EBV-EBNA1 | GSRERARGRGRGRGE |
| Viral.EBV-EBNA1 | EBV-EBNA1 | GTHGGTGAGAGAGGA |
| Viral.EBV-EBNA1 | EBV-EBNA1 | GVRRPQKRPSCIGCK |
| Viral.EBV-EBNA1 | EBV-EBNA1 | HGRGRGRGRGRGGGR |
| Viral.EBV-EBNA1 | EBV-EBNA1 | HIFAEVLKDAIKDLV |
| Viral.EBV-EBNA1 | EBV-EBNA1 | IKDLVMTKPAPTCNI |
| Viral.EBV-EBNA1 | EBV-EBNA1 | KRPRSPSSQSSSSGS |
| Viral.EBV-EBNA1 | EBV-EBNA1 | KTSLYNLRRGTALAI |
| Viral.EBV-EBNA1 | EBV-EBNA1 | LARSHVERTTDEGTW |
| Viral.EBV-EBNA1 | EBV-EBNA1 | LGEKGDTSGPEGSGG |
| Viral.EBV-EBNA1 | EBV-EBNA1 | LPFGMAPGPGPQPGP |
| Viral.EBV-EBNA1 | EBV-EBNA1 | LRESIVCYFMVFLQT |
| Viral.EBV-EBNA1 | EBV-EBNA1 | MTKPAPTCNIRVTVC |
| Viral.EBV-EBNA1 | EBV-EBNA1 | NLRRGTALAIPQCRL |
| Viral.EBV-EBNA1 | EBV-EBNA1 | PGAPGGSGSGPRHRD |
| Viral.EBV-EBNA1 | EBV-EBNA1 | PQCRLTPLSRLPFGM |
| Viral.EBV-EBNA1 | EBV-EBNA1 | PQPGPLRESIVCYFM |
| Viral.EBV-EBNA1 | EBV-EBNA1 | PRHRDGVRRPQKRPS |
| Viral.EBV-EBNA1 | EBV-EBNA1 | PSSQSSSSGSPPRRP |
| Viral.EBV-EBNA1 | EBV-EBNA1 | PTCNIRVTVCSFDDG |
| Viral.EBV-EBNA1 | EBV-EBNA1 | QKRPSCIGCKGTHGG |
| Viral.EBV-EBNA1 | EBV-EBNA1 | RGGDNHGRGRGRGRG |
| Viral.EBV-EBNA1 | EBV-EBNA1 | RGGGRPGAPGGSGSG |
| Viral.EBV-EBNA1 | EBV-EBNA1 | RGGSGGRGRGGSGGR |
| Viral.EBV-EBNA1 | EBV-EBNA1 | RGQGGSNPKFENIAE |
| Viral.EBV-EBNA1 | EBV-EBNA1 | RGRGEKRPRSPSSQS |
| Viral.EBV-EBNA1 | EBV-EBNA1 | RGRGGSGGRGRGGSG |
| Viral.EBV-EBNA1 | EBV-EBNA1 | RKKGGWFGKHRGQGG |
| Viral.EBV-EBNA1 | EBV-EBNA1 | RVTVCSFDDGVDLPP |
| Viral.EBV-EBNA1 | EBV-EBNA1 | SFDDGVDLPPWFPPM |
| Viral.EBV-EBNA1 | EBV-EBNA1 | SGGRGRGGSGGRGRG |
| Viral.EBV-EBNA1 | EBV-EBNA1 | SGPQRRGGDNHGRGR |
| Viral.EBV-EBNA1 | EBV-EBNA1 | SNPKFENIAEGLRAL |
| Viral.EBV-EBNA1 | EBV-EBNA1 | SSSGSPPRRPPPGRR |
| Viral.EBV-EBNA1 | EBV-EBNA1 | TALAIPQCRLTPLSR |
| Viral.EBV-EBNA1 | EBV-EBNA1 | TPLSRLPFGMAPGPG |
| Viral.EBV-EBNA1 | EBV-EBNA1 | VAGVFVYGGSKTSLY |
| Viral.EBV-EBNA1 | EBV-EBNA1 | VCYFMVFLQTHIFAE |
| Viral.EBV-EBNA1 | EBV-EBNA1 | VDLPPWFPPMVEGAA |
| Viral.EBV-EBNA1 | EBV-EBNA1 | VERTTDEGTWVAGVF |
| Viral.EBV-EBNA1 | EBV-EBNA1 | VFLQTHIFAEVLKDA |
| Viral.EBV-EBNA1 | EBV-EBNA1 | VLKDAIKDLVMTKPA |
| Viral.EBV-EBNA1 | EBV-EBNA1 | VYGGSKTSLYNLRRG |
| Viral.EBV-EBNA1 | EBV-EBNA1 | WFGKHRGQGGSNPKF |
| Viral.EBV-EBNA1 | EBV-EBNA1 | WFPPMVEGAAAEGDD |
| Viral.CMV-EnvelopeGlycoproteinC | Viral and >1000 | DNNTTHLSSMESVHN |
| Viral.CMV-EnvelopeGlycoproteinC | Viral and >1000 | ITHRTRRSTSDNNTT |
| Viral.CMV-EnvelopeGlycoproteinC | Viral and >1000 | KGPGPPSSDASTAAP |
| Viral.CMV-EnvelopeGlycoproteinC | Viral and >1000 | QNLFPYLVSADGTTV |
| Viral.CMV-EnvelopeGlycoproteinC | Viral and >1000 | QTRSVYSQHVTSSEA |
| Viral.CMV-EnvelopeGlycoproteinC | Viral and >1000 | RRSTSDNNTTHLSSM |
| Viral.CMV-EnvelopeGlycoproteinC | Viral and >1000 | VVYISPFYNGTNRNA |
| Viral.CMV-pp65 | Viral and >1000 | SQPSLILVSQYTPDS |
| Viral.CMV-pp66 | Viral and >1000 | QVQHTYFTGSEVENV |
| Viral.HSV-GlycoproteinC | Viral and >1000 | EHHGSHQPPPRDPTE |
| Viral.Peptid Adenovirus type 12, ORF | Viral and >1000 | DFEVVTFLKDVLPEF |
| Viral.VZV-GlycoproteinC | Viral and >1000 | AAFKQQQKTYEQYSL |
| Viral.VZV-GlycoproteinC | Viral and >1000 | CDQNKRFVNMQSSCP |
| Viral.VZV-GlycoproteinC | Viral and >1000 | DPVLTGQTYAAYCNV |
| Viral.VZV-GlycoproteinC | Viral and >1000 | GNALIRMPDLPVMLY |
| Viral.VZV-GlycoproteinC | Viral and >1000 | GTYIWRVVNTKTKNV |
| Viral.VZV-GlycoproteinC | Viral and >1000 | LFSYVSAVRIPQQKQ |
| Viral.VZV-GlycoproteinC | Viral and >1000 | NDSPINHENSEITGV |
| Viral.VZV-GlycoproteinC | Viral and >1000 | NYVIPDVKTTSDFSV |
| Viral.VZV-GlycoproteinC | Viral and >1000 | PDPAVAPTSAASRKP |
| Viral.VZV-GlycoproteinC | Viral and >1000 | PHSVRVRWTSRFGNI |
| Viral.VZV-GlycoproteinC | Viral and >1000 | TFIMPCYMRLTTGQQ |
| Viral.VZV-GlycoproteinC | Viral and >1000 | THAKENYVIPDVKTT |
| AQP4.129-163 | AQP4 | GAGILYLVTPPSVVG |
| AQP4.129-163 | AQP4 | GGLGVTMVHGNLTAG |
| AQP4.129-163 | AQP4 | GILYLVTPPSVVGGL |
| AQP4.129-163 | AQP4 | LGVTMVHGNLTAGHG |
| AQP4.129-163 | AQP4 | LVTPPSVVGGLGVTM |
| AQP4.129-163 | AQP4 | LYLVTPPSVVGGLGV |
| AQP4.129-163 | AQP4 | MVHGNLTAGHGLLVE |
| AQP4.129-163 | AQP4 | PSVVGGLGVTMVHGN |
| AQP4.129-163 | AQP4 | TPPSVVGGLGVTMVH |
| AQP4.129-163 | AQP4 | VTMVHGNLTAGHGLL |
| AQP4.198-239 | AQP4 | AIGFSVAIGHLFAIN |
| AQP4.198-239 | AQP4 | AIGHLFAINYTGASM |
| AQP4.198-239 | AQP4 | AINYTGASMNPARSF |
| AQP4.198-239 | AQP4 | ALAIGFSVAIGHLFA |
| AQP4.198-239 | AQP4 | ASCDSKRTDVTGSIA |
| AQP4.198-239 | AQP4 | ASMNPARSFGPAVIM |
| AQP4.198-239 | AQP4 | CDSKRTDVTGSIALA |
| AQP4.198-239 | AQP4 | DVTGSIALAIGFSVA |
| AQP4.198-239 | AQP4 | FGPAVIMGNWENHWI |
| AQP4.198-239 | AQP4 | FQLVFTIFASCDSKR |
| AQP4.198-239 | AQP4 | FTIFASCDSKRTDVT |
| AQP4.198-239 | AQP4 | GFSVAIGHLFAINYT |
| AQP4.198-239 | AQP4 | GHLFAINYTGASMNP |
| AQP4.198-239 | AQP4 | GNWENHWIYWVGPII |
| AQP4.198-239 | AQP4 | IFASCDSKRTDVTGS |
| AQP4.198-239 | AQP4 | LFAINYTGASMNPAR |
| AQP4.198-239 | AQP4 | LVFTIFASCDSKRTD |
| AQP4.198-239 | AQP4 | MGNWENHWIYWVGPI |
| AQP4.198-239 | AQP4 | MNPARSFGPAVIMGN |
| AQP4.198-239 | AQP4 | NYTGASMNPARSFGP |
| AQP4.198-239 | AQP4 | PARSFGPAVIMGNWE |
| AQP4.198-239 | AQP4 | PAVIMGNWENHWIYW |
| AQP4.198-239 | AQP4 | RSFGPAVIMGNWENH |
| AQP4.198-239 | AQP4 | RTDVTGSIALAIGFS |
| AQP4.198-239 | AQP4 | SIALAIGFSVAIGHL |
| AQP4.198-239 | AQP4 | SKRTDVTGSIALAIG |
| AQP4.198-239 | AQP4 | SVAIGHLFAINYTGA |
| AQP4.198-239 | AQP4 | TGASMNPARSFGPAV |
| AQP4.198-239 | AQP4 | TGSIALAIGFSVAIG |
| AQP4.198-239 | AQP4 | VIMGNWENHWIYWVG |
| AQP4.46-78 | AQP4 | FVLLSLGSTINWGGT |
| AQP4.46-78 | AQP4 | GGTEKPLPVDMVLIS |
| AQP4.46-78 | AQP4 | GSTINWGGTEKPLPV |
| AQP4.46-78 | AQP4 | KPLPVDMVLISLCFG |
| AQP4.46-78 | AQP4 | LIFVLLSLGSTINWG |
| AQP4.46-78 | AQP4 | LLSLGSTINWGGTEK |
| AQP4.46-78 | AQP4 | NWGGTEKPLPVDMVL |
| AQP4.46-78 | AQP4 | SLGSTINWGGTEKPL |
| AQP4.46-78 | AQP4 | TEKPLPVDMVLISLC |
| AQP4.46-78 | AQP4 | TINWGGTEKPLPVDM |
| AQP1.108-144 | AQP1 | ADGVNSGQGLGIEII |
| AQP1.108-144 | AQP1 | AILSGITSSLTGNSL |
| AQP1.108-144 | AQP1 | ATAILSGITSSLTGN |
| AQP1.108-144 | AQP1 | DLADGVNSGQGLGIE |
| AQP1.108-144 | AQP1 | GITSSLTGNSLGRND |
| AQP1.108-144 | AQP1 | LGRNDLADGVNSGQG |
| AQP1.108-144 | AQP1 | LSGITSSLTGNSLGR |
| AQP1.108-144 | AQP1 | NSLGRNDLADGVNSG |
| AQP1.108-144 | AQP1 | RNDLADGVNSGQGLG |
| AQP1.108-144 | AQP1 | SLTGNSLGRNDLADG |
| AQP1.108-144 | AQP1 | TGNSLGRNDLADGVN |
| AQP1.108-144 | AQP1 | TSSLTGNSLGRNDLA |
| AQP1.176-192 | AQP1 | LGHLLAIDYTGCGIN |
| AQP1.176-192 | AQP1 | VALGHLLAIDYTGCG |
| AQP1.193-215 | AQP1 | FGSAVITHNFSNHWI |
| AQP1.193-215 | AQP1 | PARSFGSAVITHNFS |
| AQP1.193-215 | AQP1 | RSFGSAVITHNFSNH |
| AQP1.193-215 | AQP1 | SAVITHNFSNHWIFW |
| AQP1.193-215 | AQP1 | VITHNFSNHWIFWVG |
| AQP1.29-56 | AQP1 | FKYPVGNNQTAVQDN |
| AQP1.29-56 | AQP1 | IGSALGFKYPVGNNQ |
| AQP1.29-56 | AQP1 | LGFKYPVGNNQTAVQ |
| AQP1.29-56 | AQP1 | NNQTAVQDNVKVSLA |
| AQP1.29-56 | AQP1 | NQTAVQDNVKVSLAF |
| AQP1.29-56 | AQP1 | SALGFKYPVGNNQTA |
| AQP1.29-56 | AQP1 | VGNNQTAVQDNVKVS |
| AQP1.29-56 | AQP1 | YPVGNNQTAVQDNVK |
| Myelin.MOG.27-57 | MOG | AGQFRVIGPRHPIRA |
| Myelin.MOG.27-57 | MOG | ALVGDEVELPCRISP |
| Myelin.MOG.27-57 | MOG | HPIRALVGDEVELPC |
| Myelin.MOG.27-57 | MOG | IGPRHPIRALVGDEV |
| Myelin.MOG.27-57 | MOG | IRALVGDEVELPCRI |
| Myelin.MOG.27-57 | MOG | PRHPIRALVGDEVEL |
| Myelin.MOG.27-57 | MOG | QFRVIGPRHPIRALV |
| Myelin.MOG.27-57 | MOG | RVIGPRHPIRALVGD |
| Myelin.MOG.27-57 | MOG | SYAGQFRVIGPRHPI |
| Myelin.MOG.88-134 | MOG | AIGEGKVTLRIRNVR |
| Myelin.MOG.88-134 | MOG | GDQAPEYRGRTELLK |
| Myelin.MOG.88-134 | MOG | GEGKVTLRIRNVRFS |
| Myelin.MOG.88-134 | MOG | GKVTLRIRNVRFSDE |
| Myelin.MOG.88-134 | MOG | GRTELLKDAIGEGKV |
| Myelin.MOG.88-134 | MOG | IRNVRFSDEGGFTCF |
| Myelin.MOG.88-134 | MOG | KDAIGEGKVTLRIRN |
| Myelin.MOG.88-134 | MOG | LLKDAIGEGKVTLRI |
| Myelin.MOG.88-134 | MOG | LRIRNVRFSDEGGFT |
| Myelin.MOG.88-134 | MOG | NVRFSDEGGFTCFFR |
| Myelin.MOG.88-134 | MOG | PEYRGRTELLKDAIG |
| Myelin.MOG.88-134 | MOG | QAPEYRGRTELLKDA |
| Myelin.MOG.88-134 | MOG | RFSDEGGFTCFFRDH |
| Myelin.MOG.88-134 | MOG | SDEGGFTCFFRDHSY |
| Myelin.MOG.88-134 | MOG | TELLKDAIGEGKVTL |
| Myelin.MOG.88-134 | MOG | VTLRIRNVRFSDEGG |
| Myelin.MOG.88-134 | MOG | YRGRTELLKDAIGEG |
| Kir4.1.83-120 | Kir4.1 | ANHTPCVVQVHTLTG |
| Kir4.1.83-120 | Kir4.1 | DLLELDPPANHTPCV |
| Kir4.1.83-120 | Kir4.1 | GVVWYLVAVAHGDLL |
| Kir4.1.83-120 | Kir4.1 | HGDLLELDPPANHTP |
| Kir4.1.83-120 | Kir4.1 | HTPCVVQVHTLTGAF |
| Kir4.1.83-120 | Kir4.1 | LDPPANHTPCVVQVH |
| Kir4.1.83-120 | Kir4.1 | LELDPPANHTPCVVQ |
| Kir4.1.83-120 | Kir4.1 | PPANHTPCVVQVHTL |
| Kir4.1.83-120 | Kir4.1 | TPCVVQVHTLTGAFL |
| Kir4.1.83-120 | Kir4.1 | VAHGDLLELDPPANH |
| Kir4.1.83-120 | Kir4.1 | VAVAHGDLLELDPPA |
| Kir4.1.83-120 | Kir4.1 | VWYLVAVAHGDLLEL |
| Kir4.1.83-120 | Kir4.1 | YLVAVAHGDLLELDP |
| Quinta.Pos.IgG.HSP60.240-259 | Quintana Peptide | AYVLLSEKKISSIQS |
| Quinta.Pos.IgG.HSP60.240-259 | Quintana Peptide | LLSEKKISSIQSIVP |
| Quinta.Pos.IgG.HSP60.240-259 | Quintana Peptide | QDAYVLLSEKKISSI |
| Quinta.Pos.IgG.HSP60.240-259 | Quintana Peptide | VLLSEKKISSIQSIV |
| Quinta.Pos.IgG.MOG.196-215 | Quintana Peptide | DPHFLRVPCWKITLF |
| Quinta.Pos.IgG.MOG.196-215 | Quintana Peptide | FLRVPCWKITLFVIV |
| Quinta.Pos.IgG.MOG.196-215 | Quintana Peptide | HFLRVPCWKITLFVI |
| Quinta.Pos.IgG.MOG.196-215 | Quintana Peptide | TFDPHFLRVPCWKIT |
| Quinta.Pos.IgG.OSP.1-20 | Quintana Peptide | ATCLQVVGFVTSFVG |
| Quinta.Pos.IgG.OSP.1-20 | Quintana Peptide | CLQVVGFVTSFVGWI |
| Quinta.Pos.IgG.OSP.1-20 | Quintana Peptide | LQVVGFVTSFVGWIG |
| Quinta.Pos.IgG.OSP.1-20 | Quintana Peptide | MVATCLQVVGFVTSF |
| Quinta.Pos.IgG.OSP.166-185 | Quintana Peptide | CLVGGCVILCCAGDA |
| Quinta.Pos.IgG.OSP.166-185 | Quintana Peptide | GGCVILCCAGDAQAF |
| Quinta.Pos.IgG.OSP.166-185 | Quintana Peptide | VGGCVILCCAGDAQA |
| Quinta.Pos.IgG.OSP.166-185 | Quintana Peptide | VLCLVGGCVILCCAG |
| Quinta.Pos.IgG.OSP.61-80 | Quintana Peptide | CKPLVDILILPGYVQ |
| Quinta.Pos.IgG.OSP.61-80 | Quintana Peptide | GLYHCKPLVDILILP |
| Quinta.Pos.IgG.OSP.61-80 | Quintana Peptide | KPLVDILILPGYVQA |
| Quinta.Pos.IgG.OSP.61-80 | Quintana Peptide | YHCKPLVDILILPGY |
| Quinta.Pos.IgG.PLP.215-232 | Quintana Peptide | FPGKVCGSNLLSICK |
| Quinta.Pos.IgG.PLP.215-232 | Quintana Peptide | GKVCGSNLLSICKTA |
| Quinta.Pos.IgG.PLP.215-232 | Quintana Peptide | KVCGSNLLSICKTAE |
| AN-2 | Control | HFRVLALARGVNASA |
| AQP-1 | Control | LGLLLSCQISIFRAL |
| bax | Control | LLGWIQDQGGWDGLL |
| bcl-2 | Control | CITLGAYLGHKGSGS |
| CYP27B1 | Control | ETFIRAVGSVFVSTL |
| EAAT2 | Control | ARQLGMYMVTVIIGL |
| EAAT2 | Control | KMLILPLIISSLITG |
| Endothelin-1 | Control | NNHKKGKDCSKLGKK |
| HIF-1alpha | Control | ESSSASPESASPQST |
| Kir4.1 | Control | FTPAISLSASGKYIA |
| Kir4.1 | Control | ILEIFITGTFLAKIA |
| Mimotop159 | Control | AQFWREQQVAEDPWH |
| Mimotop160 | Control | VTAGAMEYARGIVDQ |
| Mimotop161 | Control | NWPQVPEFVQEWHTN |
| Mimotop162 | Control | LGPKGHTSGKSRKKF |
| Mimotop163 | Control | GYQVFQPQYKENVET |
| Mimotop164 | Control | LDDPLSYMHHGAIIS |
| Mimotop165 | Control | DTWHLKFRAWLAQRS |
| Mimotop166 | Control | EGNGIYTYKNWIHTS |
| Mimotop167 | Control | ERSHLVLHFWMIRKA |
| Mimotop168 | Control | FDHPTYFPASFVHTS |
| Mimotop169 | Control | FIFHRFFPVLKVEIT |
| Mimotop170 | Control | FKKHVVHHLMVRAAM |
| Mimotop171 | Control | FRLKRPAKPTNFHLV |
| Mimotop172 | Control | GMRSPNQWLIRHVTA |
| NOGO-A | Control | AKIPGLKRKAEGSGS |
| NOGO-A | Control | DSLKFAVLMWVFTYV |
| NOGO-A | Control | ETFSDSSPIEIIDEF |
| NOGO-A | Control | TSENKTDEKKIEEKK |
| Olig2 | Control | AAAAAAAAAAAAVSS |
| Olig2 | Control | LSAELRGAMGSAGAH |
| Quinta.Prot.HSP70 | Control | KGKISEADKKKVLDK |
| S-100b | Control | GREGDKHKLKKSELK |
| Type1interferon | Control | GFLQRSSNFQCQKLL |
| Type1interferon | Control | GSGMTNKCLLQIALL |
| Type1interferon | Control | NKCLLQIALLLCFST |
| Viral.CMV-pp65 | Control | MISVLGPISGHVLKA |
| Viral.HSV-GlycoproteinC | Control | CVPEGVTFAWFLGDD |
| Viral.VZV-GlycoproteinC | Control | KTKNVISEHSITVTT |

Reference List:

Quintana FJ, Farez MF, Viglietta V, Iglesias AH, Merbl Y, Izquierdo G *et al*. Antigen microarrays identify unique serum autoantibody signatures in clinical and pathologic subtypes of multiple sclerosis. Proc Natl Acad Sci U S A 2008; 105: 18889-18894.
