# Supplementary material for: Antibody signatures in patients with histopathologically defined multiple sclerosis patterns
Source: Acta Neuropathol. 2020 Jan 16;139(3):547–64. doi: 10.1007/s00401-019-02120-x (PMC7035238; doi:10.1007/s00401-019-02120-x)
Supplement: Supplementary file 3 — Supplementary file3 (DOCX 12 kb) [file 401_2019_2120_MOESM3_ESM.docx]

**Supplementary table 2**: Demographic data of the control groups

| **Diagnosis** | **Number of patients** | **Females** | **Mean age** | **Mean disease duration (days)** |
| --- | --- | --- | --- | --- |
| Sjögren’s syndrome | 15 | 14/15 | 46.4 | unknown |
| Stroke patients | 15 | 5/15 | 64 | 4.4 |
| Healthy controls | 15 | 11/15 | 39.8 | not applicable |
