# Supplementary material for: Antibody signatures in patients with histopathologically defined multiple sclerosis patterns
Source: Acta Neuropathol. 2020 Jan 16;139(3):547–64. doi: 10.1007/s00401-019-02120-x (PMC7035238; doi:10.1007/s00401-019-02120-x)
Supplement: Supplementary file 4 — Supplementary file4 (DOCX 13 kb) [file 401_2019_2120_MOESM4_ESM.docx]

**Supplementary Table 3**: List of antibodies used for histological characterization of the brain lesions

| **Protein Target** | **Antibody type** | **Catalog #/clone** | **Source** | **Pretreatment/ dilution** |
| --- | --- | --- | --- | --- |
| Proteolipid protein (PLP) | Mouse mc | MCA839G/plpc1 | Bioyol/Serotec | Citrate+ Microwave/ 1:500 |
| Myelin basic protein (MBP) | Rabbit pc | A0623 | Dako | None/  1:2000 |
| Myelin oligodendrocyte glycoprotein (MOG) | Rabbit mc | Ab109746/EP4281 | Abcam | Citrate+ Microwave/ 1:1000 |
| 2’3’-cyclic nucleotide 3’phosphodiesterase (CNPase) | Mouse mc | SMI19R/CNP | Stenberger Monoclonals/Covance | Citrate+ Microwave/ 1:200 |
| Myelin associated glycoprotein (MAG) | Mouse mc | Ab89780 | Abcam | Citrate+ Microwave/ 1:1000 |
| Complement C9neo (C9neo) | Mouse mc | Clone B7 | Prof. Morgan Cardiff, UK | Citrate+ Microwave/ 1:50 |
| Terminal complement complex (TCC) and C9 | Rabbit anti-human; pc | - | Prof. Morgan Cardiff, UK | Citrate+ Steamer/  1:500 |
| Terminal complement complex (TCC) and C9 | Rabbit anti-rat; pc | - | Prof. Morgan Cardiff, UK | Citrate+ Microwave/ 1:6000 |
| Aquaporin-4 (AQP-4) | Rabbit pc | A5971 | Sigma-Aldrich | Citrate+ Microwave/ 1:200 |
| Aquaporin-1 (AQP-1) | Rabbit pc | Sc-20810 | Santa Cruz Biotechnology | Citrate+ Microwave/ 1:200 |
| Varicella-Zoster-Virus (VZV) | Mouse mc | Clone mix | Dianova | Citrate+ Microwave/  1:200 |
