# Supplementary material for: Antibody signatures in patients with histopathologically defined multiple sclerosis patterns
Source: Acta Neuropathol. 2020 Jan 16;139(3):547–64. doi: 10.1007/s00401-019-02120-x (PMC7035238; doi:10.1007/s00401-019-02120-x)
Supplement: Supplementary file 5 — Supplementary file5 (DOCX 43 kb) [file 401_2019_2120_MOESM5_ESM.docx]

**Supplementary table 4.** List of differentially bound peptides comparing patients with Sjögren´s syndrome and MS patterns I-III.

| **Peptide** | **Annotation Protein** | **Mean pattern I** | **Mean Sjögren´s syndrome** | **p value** |
| --- | --- | --- | --- | --- |
| GYFEDRRPSANCDPF  PEDDGEYWCVAENQY  RYNWIYIDNIPELTF  KVETMRYEDHDWTLY  NTVIIPTGWWDVGYY  ALLDVWNLEFMEHIY  GPIIGAVLAGGLYEY  TLDNDGDGECDFQEF  RVILYNRTRLDCPFF  LIFGSRQYQEGYYYN  SLVNTNAWVQRDMYF  QMPYQDITAFVEHDF  AGCRLEEEEYEDDAY  MVWALDLDDFQGSFC  TFDVSILTIDDGIFE  PYIPMDDDFQLRSFD  PLVRRIISEWFEEVY  HNEPETENHLFYDFV  SGTIGYGFRYISEEC  PWGRLCRDWDQMFAF  QYQNENITWDYTPEY  SALGSQTTFGPVFED  SPITDYVVQFEEDQF  TCTAAAYYPRNPVEF  LEMLAPYIPMDDDFQ  IIMIVISIDYSMNDF  ATVQGQNLKYQEFFW  AGPQGWHMSEFLWYY  HLHTWIQDNGGWDAF  FVNPIIQHDPNYDII  FPPFSAVYTYDASTY  TDWSYGPMIWRDWFY  PTCNIRVTVCSFDDG  QLAGAMVWALDLDDF  PELPYNNDLEVHYPY  WKHNFGPGTDFVVEY  RRLAGQFLEELRNPF  FQVMQAFPISYEQDY  NQQTFLNRAVSYQEF  QRFSDGYDNGVRHDF  FLRVPCWKITLFVIV  AYYPRNPVEFVWFED  VLFTSDEHQVDMEWF  SFDDGVDLPPWFPPM  TSDTYPNGVIVWPEF  AFLERADSVISWDIQ  WEWYTYSKNEWEIFL  HLVPGPWGRLCRDWD  ARHTNSWEWLKYEYD  SLFARRSGNERIWYD  MVATCLQVVGFVTSF  SDTVQGQLREYRAYY  AVLAGGLYEYVFCPD  QIPSVSFEDEGTYEC  KLKPGLEKDFLPLYF  QTTIGYGFRYISEEC  LRITNVSEEDSGEYF  PAVIMGNWENHWIYW  TSHIVARDWGTFEYY  TVELRQYDPVAALFF  WNTWPYYERFEHQFN  YNNSTFVMNQFVDFW  YKITPGARGAFSEEY  LQQFQKEDAALTIYE  RLEDEQVEQDYFAGW  PKEEDGSFDYSDEDN  GESYEDPPQEEYQEY  NPVEFVWFEDDHQVF  QAGDDFSRRYRRDFA  AGDAQAFGENRFYYT  RPGNQEPFVRWRDYW  CLQVVGFVTSFVGWI  HPHRVMQSVFDREWF  KLIETYFSKNYQDYE  GFYTTGAVRQIFGDY  EWTIFLYPNQEQPEW  GQSALNPYNYRVNNY  SGSGMSSFSYEPYYS  RLWNTAYSGHEVEYR  YGGCGGNRNNFDTEE  ARTGRPQQEPVWNYD  IEGMKFDRGYISPYF  PGLSYRWLLNEFPNF  DLERKIESLEEEIRF  NVRFSDEGGFTCFFR  GNWENHWIYWVGPII  QAHWENTRNEEQHWD  VWIWERDIYHPGDPR  LEETLRNLQARYEEE  YHCKPLVDILILPGY  YQARTALWEIVHHDW  RFSDEGGFTCFFRDH  SYMQTPRHDEYFAGF  SDVWWGGADTDYADG  TSYRDDEMSRPQWEW  PEPESVIGYSGEDYP  QLQNNTLEGEQYTEY  KLTHDVELNLDYERY  LVTPPSVVGGLGVTM  VPPEFKPDHFRDHEY  WMMPSVNDYDDNANY  VVQRSDWAPQPQYNE  LDDTNHERERLEQLF  LVDSKSQHMWPADVF  TILSMDATTEGTYIW  FGPAVIMGNWENHWI  IILDVRAIPIYIDEV  GGCVILCCAGDAQAF  PSIELRDVRYYLDEF  LLFLLEEYKNYLDAA  TDDSGTSHYDVMDYE  QAIRETVELRQYDPV  YVVQFEEDQFQPGVW  LSAEPAPDFSDYSEM  RVEDPYQWTSASDAF  GVVWYLVAVAHGDLL  GKVCGSNLLSICKTA  PARSFGPAVIMGNWE  DGMYDIPSISEYYTY  GNTPVIQDYPLIYEI  IGSALGFKYPVGNNQ  DFEVVTFLKDVLPEF  ETGPCRAMISRWYFD  GLDRTGKGERNVLIF  GTYIWRVVNTKTKNV  YLVAVAHGDLLELDP  VCYFMVFLQTHIFAE  GPPPGGQPDPELLQF  SSPEPNSPSEYCFYV  GDANNSPITDYVVQF  YNPASPDFEEFDSAV  GSTMDIDHEWERVQF  AAPANAPNFPEGEYH  QVSGYVDWGLRWFEM  AGNILYEHEMPPEPF  LEQLFEMADQYQYQV  HFLRVPCWKITLFVI  EAGTLAYYEICDFLR  GPRGQGDGGRRKKGG  AETAQVLGLTPWMDY  IRILVLDDTNHERER  RLGVRIAHYYEVEPF  GGRPEEYEGEYQCFA  AVTHGMNYGRMPFDF  DHMAWEEQEGELDFQ  CIGCKGTHGGTGAGA  ATCLQVVGFVTSFVG  SFEDEGTYECEAENS  IRNVRFSDEGGFTCF  SLGSTINWGGTEKPL  SAVITHNFSNHWIFW  LDNLPNRWDASDYDD  IEYQPMVDAEEPDWQ  AGAGGGAGGAGGAGA  SLSPLSAASFKEHEY  HYREDAALDQEQSMV  SDEELVTTERKTPRV  QPGNTISAGQEDFPS  DRRRHIVEIVTWERY  HIDTWEWNDVTLYGM  PDSWSPMDASDDIFI  FGSAVITHNFSNHWI  AGGAGGAGAGGAGGA  AETYALVGQQVTLEC  PAAGAPLMDFGNDFV  GAGGGAGGAGAGGAG  DPHLCDFIETHYLNE  GAGGAGGAGAGGAGG  CLVLICEPIPHPSNI  YQDYEYLINVIHAFQ  NQTAVQDNVKVSLAF  RNLQARYEEEVLSRE  LFAINYTGASMNPAR  APKQSGNTPVIQDYP  RSPGWETFMNTGFIH  SDEGGFTCFFRDHSY  DDVVQWMLSIERPNF  GGAGGAGAGGGAGGA  TYVSMVYYIQNPIYT  GSPNKHDWGDDRPDN  AEGDDGDDGDEGGDG  WARLPSAPPTAFQER  RYEEEVLSREDAEGR  EEPEAAYRLIQGPQY  GAGGAGAGGAGGAGA  TFPIPSTVISWNDAA  LFSYVSAVRIPQQKQ  RRSTSDNNTTHLSSM  TSGSDSDEELVTTER  WNGDHENHMRFNNVD  PAWEPHRYVARLFEL  EEEEYEDDAYGHYEA  MGNWENHWIYWVGPI  KTFMVYSAHSGNGKY  LLSLGSTINWGGTEK  FPGKVCGSNLLSICK  AGAGGGAGGAGGAGG  RGRGGSGGRGRGGSG  VIMGNWENHWIYWVG  MREQTLYVMIREPYR  KPLPVDMVLISLCFG  GAGAGGGAGGAGAGG  ADIPTDAAEKWQYPE  RVTVCSFDDGVDLPP  GAGAGGAGAGGAGGA  AGGAGAGGGAGGAGG  PQPGPLRESIVCYFM  CKPLVDILILPGYVQ  AGGAGGAGGAGAGGA  HSVRWRHDWLHSSNF  GGAGAGGGAGAGGAG  ADGVNSGQGLGIEII  HMQSSEHAQRRGDYP  GAGAGGGAGGAGGAG  VERTTDEGTWVAGVF  SDMYLVPAAMFRDPF  SPGGMVNQVHHGDFA  GAGGGAGGAGGAGAG  GAGGAGAGGGAGGAG  NISTIATVEETNQTD  QVTFTVWQIYEIDYS  GYEPEELLGRSIYEY  GVNNAYFNWNSSDQF  NRNGSYFQDNNGLNY  RWVSQSVNIPTAYEV  CFIIENISTIATVEE  SDTDENLMREWPSNE  HTPCVVQVHTLTGAF  DGEPDVPPGAIEQGP  GAGAGGAGGAGAGGA  AGAGGGAGGAGAGGG  GGGAGAGGAGGAGGA  AGGAGGAGAGGAGAG  GAGGGAGGAGAGGGA  EVEEEEADDDEDDED  LEDVQPHDLGKVGEV  LASVQVDASQNWDEN  LNETVTEVPEETKMV  SRHSLDMKFSYCDER  GHLFAINYTGASMNP  IDFRSGGRPEEYEGE  LGHLLAIDYTGCGIN  GRTELLKDAIGEGKV  VAVAHGDLLELDPPA  GGAGGAGAGGGAGAG  VYPELQITNVVEANQ  AFFNVRKNNQDQKMQ  TGAGAGAGGAGAGGA  KDEFDHIGMEGMDAT  PYDKLNEIPLIWRPD  GAGGAGGAGGAGAGG  GAGGAGGAGAGGGAG  KDAIGEGKVTLRIRN  AGAGGGAGAGGGAGG  GAGGGAGAGGGAGGA | Glutaminsynthetase  Myelin.MAG  Mimotop136  Mimotop41  Mimotop13  Mimotop5  AQP4  S-100b  Neurofascin186  Mimotop118  Mimotop34  Mimotop32  AN-2  CHI3L1  HSP70  HIF-1alpha  Mimotop16  Mimotop135  Kir4.1  CYP27B1  Mimotop119  Contactin-2  Neurofascin186  Viral.HSV-GlycoproteinC  HIF-1alpha  Mimotop134  Viral.CMV-pp65  Mimotop121  bcl-2  Mimotop158  Viral.VZV-GlycoproteinC  Mimotop149  Viral.EBV-EBNA1  CHI3L1  Mimotop44  Neurofascin186  Myelin.MOG  Mimotop153  Mimotop120  Mimotop133  Quinta.Pos.IgG.MOG.196-215  Viral.HSV-GlycoproteinC  Mimotop130  Viral.EBV-EBNA1  Mimotop51  Viral.CMV-EnvelopeGlycoproteinC  Mimotop46  CYP27B1  Mimotop43  Mimotop151  Quinta.Pos.IgG.OSP.1-20  Neurofascin186  AQP4  Contactin-2  Myelin.CNP  Kir4.1  Neurofascin186  AQP4.198-239  Mimotop11  Viral.CMV-pp65  Mimotop58  Mimotop50  Myelin.CNP  Type1interferon  Mimotop48  Neurofascin186  Beta-Synuclein  Viral.HSV-GlycoproteinC  bcl-2  Myelin.OSP  Mimotop10  Quinta.Pos.IgG.OSP.1-20  Mimotop155  Myelin.PLP  Myelin.PLP  Mimotop122  Mimotop145  Neurofilament68kD  Mimotop38  AmyloidBeta  Mimotop57  HSP60  Contactin-2  GFAP  Myelin.MOG.88-134  AQP4.198-239  Mimotop127  Mimotop8  Neurofilament68kD  Quinta.Pos.IgG.OSP.61-80  Mimotop125  Myelin.MOG.88-134  Mimotop68  AmyloidBeta  Mimotop148  Neurofascin186  Mimotop14  sTFR  AQP4.129-163  Mimotop6  Mimotop4  Mimotop114  Myelin.CNP  Mimotop72  Viral.VZV-GlycoproteinC  AQP4.198-239  Mimotop147  Quinta.Pos.IgG.OSP.166-185  Mimotop15  BDNF  Mimotop56  Viral.CMV-pp65  Neurofascin186  NOGO-A  Mimotop75  Kir4.1.83-120  Quinta.Pos.IgG.PLP.215-232  AQP4.198-239  Mimotop154  IFNAR1  AQP1.29-56  Viral.Peptid Adenovirus type 12, ORF  AmyloidBeta  HSP70  Viral.VZV-GlycoproteinC  Kir4.1.83-120  Viral.EBV-EBNA1  AN-2  HIF-1alpha  Neurofascin186  Mimotop132  Mimotop49  Mimotop62  Mimotop54  AN-2  Myelin.CNP  Quinta.Pos.IgG.MOG.196-215  CHI3L1  Viral.EBV-EBNA1  Contactin-2  Myelin.CNP  Mimotop117  Neurofascin186  Mimotop152  Mimotop146  Viral.EBV-EBNA1  Quinta.Pos.IgG.OSP.1-20  Contactin-2  Myelin.MOG.88-134  AQP4.46-78  AQP1.193-215  Mimotop156  Mimotop128  Viral.EBV-EBNA1  NOGO-A  Mimotop126  Viral.CMV-pp65  NOGO-A  Mimotop45  CHI3L1  Mimotop65  AQP1.193-215  Viral.EBV-EBNA1  Contactin-2  NOGO-A  Viral.EBV-EBNA1  FerritinHeavyChain  Viral.EBV-EBNA1  HIF-1alpha  Myelin.PLP  AQP1.29-56  Neurofilament68kD  AQP4.198-239  IFNAR1  Mimotop91  Myelin.MOG.88-134  Mimotop137  Viral.EBV-EBNA1  Mimotop67  Mimotop42  Viral.EBV-EBNA1  Mimotop113  Neurofilament68kD  AN-2  Viral.EBV-EBNA1  Mimotop39  Viral.VZV-GlycoproteinC  Viral.CMV-EnvelopeGlycoproteinC  Viral.CMV-pp65  Mimotop141  Mimotop76  AN-2  AQP4.198-239  Mimotop85  AQP4.46-78  Quinta.Pos.IgG.PLP.215-232  Viral.EBV-EBNA1  Viral.EBV-EBNA1  AQP4.198-239  Mimotop71  AQP4.46-78  Viral.EBV-EBNA1  Mimotop21  Viral.EBV-EBNA1  Viral.EBV-EBNA1  Viral.EBV-EBNA1  Viral.EBV-EBNA1  Quinta.Pos.IgG.OSP.61-80  Viral.EBV-EBNA1  Mimotop139  Viral.EBV-EBNA1  AQP1.108-144  Mimotop104  Viral.EBV-EBNA1  Viral.EBV-EBNA1  Glutaminsynthetase  Mimotop103  Viral.EBV-EBNA1  Viral.EBV-EBNA1  IFNAR1  Mimotop12  HIF-1alpha  Mimotop60  Mimotop7  Mimotop138  IFNAR1  Mimotop77  Kir4.1.83-120  Viral.EBV-EBNA1  Viral.EBV-EBNA1  Viral.EBV-EBNA1  Viral.EBV-EBNA1  Viral.EBV-EBNA1  Viral.EBV-EBNA1  AmyloidBeta  HSP60  Mimotop131  EAAT2  HIF-1alpha  AQP4.198-239  Neurofascin186  AQP1.176-192  Myelin.MOG.88-134  Kir4.1.83-120  Viral.EBV-EBNA1  AmyloidBeta  Mimotop89  Viral.EBV-EBNA1  Mimotop2  Mimotop63  Viral.EBV-EBNA1  Viral.EBV-EBNA1  Myelin.MOG.88-134  Viral.EBV-EBNA1  Viral.EBV-EBNA1 | 10.1658  10.2389  10.1335  10.2625  10.3915  10.2437  10.2609  10.3022  10.2247  10.2284  10.2749  10.1343  10.3388  10.3228  10.1690  10.3241  10.3566  10.0486  10.2658  10.3804  10.1936  10.1964  10.2273  10.0942  10.2136  10.2092  10.3075  10.4593  10.2715  10.0681  10.1149  10.6091  10.1591  10.3761  10.2039  10.1414  10.1749  10.2293  10.1755  10.1715  10.0085  10.3291  10.3657  10.3065  10.2864  10.1575  10.3461  10.4036  10.2975  10.3264  10.0727  10.2515  10.2429  10.3029  10.2718  10.2520  10.2129  10.3754  10.2643  10.1256  10.1149  10.2855  10.2945  10.0616  10.2802  10.1290  10.2266  10.1750  10.1695  10.0760  10.4343  10.1734  10.3848  10.1993  10.2232  10.2366  10.0035  10.2864  10.2680  10.1251  10.1822  10.3069  10.1853  10.2846  10.2792  10.2285  10.1829  10.2744  10.2313  10.1679  10.0857  10.1416  10.2586  10.2448  10.4100  10.2578  10.1553  10.3020  10.2363  10.1051  10.2171  10.0941  10.1721  10.1650  10.2233  10.2091  10.2179  10.1316  10.3900  10.1545  10.2095  10.0919  10.2926  10.2978  10.1413  10.0506  10.0588  10.3606  10.2454  10.1211  10.0753  10.2035  10.4116  10.1568  9.9720  10.0908  10.0495  10.1869  10.1583  10.0793  10.1695  10.2247  10.0962  10.5315  10.1876  10.1319  10.0181  10.3297  10.3611  10.4085  10.1709  10.2673  10.2603  10.3595  10.2434  10.1813  10.0283  10.1656  10.2819  10.2309  10.1358  10.2894  10.3029  10.4062  10.1307  10.1391  10.1015  10.1018  10.3665  10.2092  10.3176  9.9515  10.5159  10.1438  10.2673  10.5358  10.1019  10.4684  9.9807  10.0327  9.9580  10.2011  10.2110  10.0522  10.1169  10.1588  10.1541  10.7639  10.0544  10.1528  10.5101  10.1283  10.3010  10.1138  10.3304  10.0899  9.9949  10.0476  10.2185  10.0298  10.2855  10.2451  10.2707  10.1384  10.2471  10.1661  10.4512  10.3473  10.3625  10.2779  10.2544  10.3967  10.2006  10.0875  10.5326  10.6471  10.3361  10.1117  10.4073  10.0746  10.8267  10.1777  10.1619  10.4053  10.2085  10.3786  9.9969  10.3736  10.6515  10.3519  10.2785  10.4550  10.1206  10.1290  10.1101  10.4166  10.1844  9.9283  10.6545  10.4478  10.4456  10.4745  10.5176  10.5788  10.3238  10.6209  10.0672  10.2016  10.2522  9.9908  10.3674  10.1446  10.1983  10.0550  10.7941  10.0228  10.0926  10.4430  10.2325  10.3051  10.3293  10.8234  10.1240  10.9735  11.1397 | 10.5810  10.5749  10.5262  10.5301  10.8349  10.5983  10.6028  10.7174  10.6558  10.6476  10.6023  10.4897  10.6101  10.5978  10.5748  10.6596  10.7405  10.5644  10.4677  10.7274  10.5921  10.5168  10.6182  10.3606  10.4468  10.5482  10.6683  10.8756  10.6018  10.3759  10.3909  11.0524  10.3578  10.7865  10.5183  10.4419  10.4027  10.6530  10.4822  10.5267  10.1893  10.7305  10.7017  10.5070  10.5950  10.3368  10.6326  10.7386  10.5964  10.6661  10.2224  10.5700  10.5258  10.8151  10.4893  10.4890  10.5643  10.6621  10.6304  10.4364  10.4578  10.6392  10.5541  10.2459  10.5354  10.3712  10.5183  10.6329  10.3629  10.2789  10.7160  10.3568  10.6637  10.4845  10.5088  10.4846  10.2113  10.5709  10.4310  10.2965  10.4218  10.5264  10.4906  10.4624  10.4431  10.3874  10.4108  10.4618  10.3787  10.3512  10.3880  10.3482  10.5105  10.3963  10.7021  10.5424  10.3808  10.5635  10.0937  10.4487  10.4955  10.2882  10.3403  10.3666  10.4917  10.4093  10.5200  10.2753  10.8231  10.2915  10.4505  10.2541  10.4925  10.6134  10.3739  10.1545  9.9607  10.5014  10.6630  10.4786  9.9854  10.4389  10.6651  10.2992  9.8587  10.2408  10.1501  10.4971  10.3853  10.2427  10.7682  10.3841  10.2447  10.6908  10.3725  10.3030  10.1302  10.6214  10.2010  10.5938  10.3161  10.6139  10.5006  10.5545  10.3888  10.0726  10.1352  10.3809  10.5145  10.1215  10.3455  10.6552  10.5000  11.2444  10.2607  10.0131  10.9457  10.2133  10.5146  10.3237  10.5624  10.0835  11.2894  10.2558  10.4954  11.4766  10.3158  11.3046  10.2361  10.2378  10.0379  10.3049  10.1430  10.1584  10.2023  10.5482  10.2925  11.9664  10.1715  10.2811  11.6224  10.2914  10.3791  10.3734  11.0746  10.1714  9.9214  9.9620  10.8901  10.2312  10.5082  10.4952  10.4152  10.0615  10.1728  10.0973  11.0928  10.2785  10.4826  10.3809  10.3582  10.9828  10.3539  10.2403  11.2850  11.4988  10.4656  10.2453  10.9779  10.2157  11.7151  10.3048  10.2450  10.9459  10.3358  10.5095  10.1243  11.0549  11.4926  11.4655  10.6176  10.7069  10.3764  10.3071  10.6139  11.4033  10.3049  10.0207  12.1061  11.0843  11.0417  11.0003  11.2662  11.3531  10.4716  10.1777  10.2294  10.9192  10.3369  9.9084  10.5444  10.2078  10.1169  10.1399  11.8397  10.2410  10.0190  10.9536  10.1660  10.4181  10.8111  11.7281  10.0589  12.0655  12.2886 | 0.0001  0.0001  0.0001  0.0001  0.0001  0.0001  0.0001  0.0001  0.0001  0.0001  0.0001  0.0001  0.0001  0.0001  0.0001  0.0001  0.0001  0.0001  0.0001  0.0001  0.0001  0.0001  0.0001  0.0001  0.0001  0.0001  0.0001  0.0001  0.0001  0.0001  0.0001  0.0001  0.0001  0.0001  0.0001  0.0001  0.0001  0.0001  0.0001  0.0001  0.0001  0.0001  0.0001  0.0001  0.0001  0.0001  0.0001  0.0001  0.0001  0.0001  0.0001  0.0001  0.0001  0.0001  0.0001  0.0001  0.0001  0.0001  0.0001  0.0001  0.0001  0.0001  0.0001  0.0001  0.0001  0.0001  0.0001  0.0001  0.0001  0.0001  0.0001  0.0001  0.0002  0.0002  0.0002  0.0002  0.0002  0.0002  0.0002  0.0002  0.0002  0.0002  0.0002  0.0002  0.0002  0.0002  0.0002  0.0002  0.0002  0.0002  0.0002  0.0002  0.0003  0.0003  0.0003  0.0004  0.0004  0.0004  0.0005  0.0006  0.0006  0.0006  0.0006  0.0006  0.0006  0.0007  0.0007  0.0007  0.0007  0.0007  0.0008  0.0008  0.0009  0.0010  0.0010  0.0010  0.0012  0.0013  0.0014  0.0016  0.0018  0.0019  0.0021  0.0021  0.0021  0.0022  0.0024  0.0024  0.0026  0.0027  0.0027  0.0028  0.0029  0.0033  0.0034  0.0034  0.0035  0.0036  0.0037  0.0038  0.0038  0.0040  0.0054  0.0058  0.0060  0.0061  0.0061  0.0061  0.0062  0.0072  0.0073  0.0074  0.0078  0.0078  0.0083  0.0083  0.0086  0.0087  0.0091  0.0104  0.0106  0.0107  0.0109  0.0109  0.0109  0.0122  0.0124  0.0124  0.0125  0.0127  0.0132  0.0132  0.0133  0.0138  0.0138  0.0138  0.0138  0.0139  0.0146  0.0147  0.0152  0.0152  0.0152  0.0152  0.0152  0.0153  0.0161  0.0164  0.0164  0.0170  0.0171  0.0178  0.0181  0.0182  0.0187  0.0188  0.0188  0.0188  0.0188  0.0188  0.0192  0.0192  0.0192  0.0206  0.0211  0.0213  0.0213  0.0219  0.0226  0.0229  0.0232  0.0232  0.0237  0.0237  0.0238  0.0243  0.0248  0.0248  0.0254  0.0263  0.0268  0.0276  0.0285  0.0294  0.0298  0.0310  0.0322  0.0334  0.0347  0.0347  0.0357  0.0358  0.0358  0.0365  0.0365  0.0378  0.0385  0.0393  0.0398  0.0402  0.0402  0.0402  0.0402  0.0414  0.0428  0.0439  0.0439  0.0471  0.0479  0.0480  0.0490  0.0495  0.0495  0.0495  0.0499 |
| **Peptide** | **Annotation Protein** | **Mean pattern II** | **Mean Sjögren´s syndrome** | **P value** |
| GYFEDRRPSANCDPF  ALLDVWNLEFMEHIY  NTVIIPTGWWDVGYY  KVETMRYEDHDWTLY  PWGRLCRDWDQMFAF  RYNWIYIDNIPELTF  TLDNDGDGECDFQEF  GPIIGAVLAGGLYEY  SGTIGYGFRYISEEC  TFDVSILTIDDGIFE  ATVQGQNLKYQEFFW  PELPYNNDLEVHYPY  FVNPIIQHDPNYDII  MVWALDLDDFQGSFC  PYIPMDDDFQLRSFD  PEDDGEYWCVAENQY  PLVRRIISEWFEEVY  AVLAGGLYEYVFCPD  QMPYQDITAFVEHDF  AGPQGWHMSEFLWYY  SALGSQTTFGPVFED  FPPFSAVYTYDASTY  CLQVVGFVTSFVGWI  RVILYNRTRLDCPFF  AGCRLEEEEYEDDAY  WEWYTYSKNEWEIFL  QYQNENITWDYTPEY  VLFTSDEHQVDMEWF  SPITDYVVQFEEDQF  TSDTYPNGVIVWPEF  NQQTFLNRAVSYQEF  LIFGSRQYQEGYYYN  QIPSVSFEDEGTYEC  ARHTNSWEWLKYEYD  LEMLAPYIPMDDDFQ  HNEPETENHLFYDFV  GNWENHWIYWVGPII  TDWSYGPMIWRDWFY  QLAGAMVWALDLDDF  QRFSDGYDNGVRHDF  FQVMQAFPISYEQDY  QTTIGYGFRYISEEC  IIMIVISIDYSMNDF  HLVPGPWGRLCRDWD  HLHTWIQDNGGWDAF  SLVNTNAWVQRDMYF  RPGNQEPFVRWRDYW  QAHWENTRNEEQHWD  MVATCLQVVGFVTSF  AFLERADSVISWDIQ  VWIWERDIYHPGDPR  YNNSTFVMNQFVDFW  YHCKPLVDILILPGY  SDTVQGQLREYRAYY  TSHIVARDWGTFEYY  YKITPGARGAFSEEY  RLEDEQVEQDYFAGW  WKHNFGPGTDFVVEY  PAVIMGNWENHWIYW  YQARTALWEIVHHDW  TILSMDATTEGTYIW  GFYTTGAVRQIFGDY  FLRVPCWKITLFVIV  PKEEDGSFDYSDEDN  SLFARRSGNERIWYD  YGGCGGNRNNFDTEE  IILDVRAIPIYIDEV  TSYRDDEMSRPQWEW  TDDSGTSHYDVMDYE  AYYPRNPVEFVWFED  SFDDGVDLPPWFPPM  FGPAVIMGNWENHWI  AGDAQAFGENRFYYT  KLIETYFSKNYQDYE  PTCNIRVTVCSFDDG  GTYIWRVVNTKTKNV  IEGMKFDRGYISPYF  LRITNVSEEDSGEYF  LDDTNHERERLEQLF  LEETLRNLQARYEEE  WNTWPYYERFEHQFN  AETAQVLGLTPWMDY  RLWNTAYSGHEVEYR  KLTHDVELNLDYERY  VIMGNWENHWIYWVG  TVELRQYDPVAALFF  LLFLLEEYKNYLDAA  DLERKIESLEEEIRF  YVVQFEEDQFQPGVW  RRLAGQFLEELRNPF  HPHRVMQSVFDREWF  QVSGYVDWGLRWFEM  QAGDDFSRRYRRDFA  ETGPCRAMISRWYFD  KLKPGLEKDFLPLYF  NVRFSDEGGFTCFFR  RFSDEGGFTCFFRDH  TCTAAAYYPRNPVEF  ARTGRPQQEPVWNYD  GYEPEELLGRSIYEY  HFLRVPCWKITLFVI  EWTIFLYPNQEQPEW  VVQRSDWAPQPQYNE  PARSFGPAVIMGNWE  DFEVVTFLKDVLPEF  LQQFQKEDAALTIYE  SGSGMSSFSYEPYYS  AAPANAPNFPEGEYH  RLGVRIAHYYEVEPF  PEPESVIGYSGEDYP  GESYEDPPQEEYQEY  SDVWWGGADTDYADG  WMMPSVNDYDDNANY  NPVEFVWFEDDHQVF  DRRRHIVEIVTWERY  GSTMDIDHEWERVQF  PGLSYRWLLNEFPNF  AETYALVGQQVTLEC  SLSPLSAASFKEHEY  VPPEFKPDHFRDHEY  LVDSKSQHMWPADVF  SSPEPNSPSEYCFYV  AGNILYEHEMPPEPF  DPHLCDFIETHYLNE  QLQNNTLEGEQYTEY  FPGKVCGSNLLSICK  SYMQTPRHDEYFAGF  LEQLFEMADQYQYQV  AVTHGMNYGRMPFDF  LSAEPAPDFSDYSEM  GPPPGGQPDPELLQF  EAGTLAYYEICDFLR  ADGVNSGQGLGIEII  VCYFMVFLQTHIFAE  YNPASPDFEEFDSAV  GVVWYLVAVAHGDLL  GDANNSPITDYVVQF  HIDTWEWNDVTLYGM  GNTPVIQDYPLIYEI  HMQSSEHAQRRGDYP  GQSALNPYNYRVNNY  RVEDPYQWTSASDAF  PSIELRDVRYYLDEF  SAVITHNFSNHWIFW  DHMAWEEQEGELDFQ  KPLPVDMVLISLCFG  GGCVILCCAGDAQAF  DGMYDIPSISEYYTY  TFPIPSTVISWNDAA  GSPNKHDWGDDRPDN  QVTFTVWQIYEIDYS  APKQSGNTPVIQDYP  LGNYSCLATSHMDFS  FGSAVITHNFSNHWI  GLDRTGKGERNVLIF  DRHDEGAAQGDDDVW  GRTELLKDAIGEGKV  CLVLICEPIPHPSNI  IRNVRFSDEGGFTCF  TSGSDSDEELVTTER  PAWEPHRYVARLFEL  GKVCGSNLLSICKTA  SRHSLDMKFSYCDER  SFEDEGTYECEAENS  YLVAVAHGDLLELDP  SGGRGRGGSGGRGRG  PYDKLNEIPLIWRPD  TWYWMYYGASLSSEW  VEKSRAWISDGNNRF  LVTPPSVVGGLGVTM  KRPRSPSSQSSSSGS  MREQTLYVMIREPYR  GHLFAINYTGASMNP  AATRKPDPAVAPTSA  QPGNTISAGQEDFPS  RSFGPAVIMGNWENH  QAIRETVELRQYDPV  GGKPNHPSEVDIYRE  HMQIERWYNFGEEDR  AFFNVRKNNQDQKMQ  PQPGPLRESIVCYFM  SDEELVTTERKTPRV  RSPGWETFMNTGFIH  KTFMVYSAHSGNGKY  NRNGSYFQDNNGLNY  ARENNHPVWGIDVSL  RRSTSDNNTTHLSSM  IRILVLDDTNHERER  ALAIGFSVAIGHLFA  LMRWSANEHDATLDQ  LKAESTVAPEEDTDE  LARSHVERTTDEGTW  EEEEYEDDAYGHYEA  MGNWENHWIYWVGPI  SDEGGFTCFFRDHSY  WNGDHENHMRFNNVD  VLKDAIKDLVMTKPA  PDSWSPMDASDDIFI  NETIYNTTLKYGDVV  TSAASRKPDPAVAPT  GPRGQGDGGRRKKGG  RVTVCSFDDGVDLPP  DDVVQWMLSIERPNF  ASTNTHGEHWRSFND  AYVLLSEKKISSIQS  TYVSMVYYIQNPIYT  HPDYHLDHNYMLVER  IDFRSGGRPEEYEGE  YQDYEYLINVIHAFQ  DSDMVNEFKLELVEK  ATCLQVVGFVTSFVG  LFSYVSAVRIPQQKQ  SRKPDPAVAPTSAAS  RNLQARYEEEVLSRE  TRKPDPAVAPTSAAS  VYGGSKTSLYNLRRG  CKPLVDILILPGYVQ  EVEEEEADDDEDDED  DEAPGMFPENDAWYT  IEYQPMVDAEEPDWQ  VERTTDEGTWVAGVF  DDFPIDFDKPGGHYR  SPGGMVNQVHHGDFA  ATRKPDPAVAPTSAA  VWYLVAVAHGDLLEL  RWVSQSVNIPTAYEV  SLGSTINWGGTEKPL  ASRKPDPAVAPTSAA  SAASRKPDPAVAPTS  ERQDEHGFISREFHR  CFIIENISTIATVEE  GLYHCKPLVDILILP  LGVTMVHGNLTAGHG  TPLSRLPFGMAPGPG  LNETVTEVPEETKMV  LESLEHDAMMEYEQF  DLLELDPPANHTPCV  NISTIATVEETNQTD  GGRPEEYEGEYQCFA  LIFVLLSLGSTINWG  SELVEDSSPDSEPVD  AEGDDGDDGDEGGDG  PTSAASRKPDPAVAP  GGLGVTMVHGNLTAG  AGIKMHRGTSEHDYP  PTSAATRKPDPAVAP  GRGRGRGGGRPGAPG  RYEEEVLSREDAEGR  AAFKQQQKTYEQYSL  RGRGGSGGRGRGGSG  LLSLGSTINWGGTEK  IETYDGVQDFNYLTW  AGAGGGAGGAGGAGA  GFSVAIGHLFAINYT  LQVVGFVTSFVGWIG  SQPSLILVSQYTPDS  ADIPTDAAEKWQYPE  CLVGGCVILCCAGDA  NAEHNHSEQSAPQMG  QDQGGWDGLLSYFGT  RKKGGWFGKHRGQGG  GLRALLARSHVERTT  AIGHLFAINYTGASM  LASVQVDASQNWDEN  SDMYLVPAAMFRDPF  RGGGRPGAPGGSGSG  PAAGAPLMDFGNDFV  WKRWGQDEDDWGYHD  ENIAEGLRALLARSH  MTRGRLKAESTVAPE  PSSQSSSSGSPPRRP  AVPGQGPPPGGQPDP  GDDGDEGGDGDEGEE  IRALVGDEVELPCRI  HGRGRGRGRGRGGGR  AGAGGGAGGAGGAGG  HYREDAALDQEQSMV  VITHNFSNHWIFWVG  MNPARSFGPAVIMGN  HSVRWRHDWLHSSNF  VAHGDLLELDPPANH  RKPDPAVAPTSAASR  KDEFDHIGMEGMDAT  RQSPWPFYTRRVDFP  YPVGNNQTAVQDNVK  TFIMPCYMRLTTGQQ  AGQFRVIGPRHPIRA  VYPELQITNVVEANQ  GAAQGDDDVWTSGSD  QQDMNGILLGYEIRY  VAVAHGDLLELDPPA  WARLPSAPPTAFQER  KDAIGEGKVTLRIRN  ASMNPARSFGPAVIM  AGGAGGAGAGGAGGA  GNALIRMPDLPVMLY  PFHSPSRLFDQFFGE  IGSALGFKYPVGNNQ  GAGAGGGAGGAGGAG  VLLSEKKISSIQSIV  GAGGAGGAGAGGAGG | Glutaminsynthetase  Mimotop5  Mimotop13  Mimotop41  CYP27B1  Mimotop136  S-100b  AQP4  Kir4.1  HSP70  Viral.CMV-pp65  Mimotop44  Mimotop158  CHI3L1  HIF-1alpha  Myelin.MAG  Mimotop16  AQP4  Mimotop32  Mimotop121  Contactin-2  Viral.VZV-GlycoproteinC  Quinta.Pos.IgG.OSP.1-20  Neurofascin186  AN-2  Mimotop46  Mimotop119  Mimotop130  Neurofascin186  Mimotop51  Mimotop120  Mimotop118  Contactin-2  Mimotop43  HIF-1alpha  Mimotop135  AQP4.198-239  Mimotop149  CHI3L1  Mimotop133  Mimotop153  Kir4.1  Mimotop134  CYP27B1  bcl-2  Mimotop34  Mimotop10  Mimotop127  Quinta.Pos.IgG.OSP.1-20  Viral.CMV-EnvelopeGlycoproteinC  Mimotop8  Mimotop50  Quinta.Pos.IgG.OSP.61-80  Neurofascin186  Mimotop11  Myelin.CNP  Mimotop48  Neurofascin186  AQP4.198-239  Mimotop125  Viral.VZV-GlycoproteinC  Myelin.PLP  Quinta.Pos.IgG.MOG.196-215  Neurofascin186  Mimotop151  AmyloidBeta  Mimotop147  Mimotop148  Mimotop56  Viral.HSV-GlycoproteinC  Viral.EBV-EBNA1  AQP4.198-239  Myelin.OSP  Myelin.PLP  Viral.EBV-EBNA1  Viral.VZV-GlycoproteinC  HSP60  Neurofascin186  Myelin.CNP  Neurofilament68kD  Mimotop58  Contactin-2  Mimotop38  sTFR  AQP4.198-239  Viral.CMV-pp65  BDNF  GFAP  Neurofascin186  Myelin.MOG  Mimotop155  Mimotop54  bcl-2  AmyloidBeta  Myelin.CNP  Myelin.MOG.88-134  Myelin.MOG.88-134  Viral.HSV-GlycoproteinC  Mimotop57  HIF-1alpha  Quinta.Pos.IgG.MOG.196-215  Mimotop122  Mimotop114  AQP4.198-239  Viral.Peptid Adenovirus type 12, ORF  Type1interferon  Neurofilament68kD  Mimotop62  Mimotop117  Neurofascin186  Beta-Synuclein  AmyloidBeta  Mimotop4  Viral.HSV-GlycoproteinC  Mimotop45  Mimotop49  Contactin-2  Contactin-2  NOGO-A  Mimotop6  Mimotop72  HIF-1alpha  AN-2  FerritinHeavyChain  Mimotop14  Quinta.Pos.IgG.PLP.215-232  Mimotop68  Myelin.CNP  Mimotop152  NOGO-A  AN-2  CHI3L1  AQP1.108-144  Viral.EBV-EBNA1  Mimotop132  Kir4.1.83-120  Neurofascin186  CHI3L1  IFNAR1  Mimotop104  Mimotop145  Mimotop75  Mimotop15  AQP1.193-215  Mimotop146  AQP4.46-78  Quinta.Pos.IgG.OSP.166-185  Mimotop154  Mimotop39  Mimotop42  Mimotop12  IFNAR1  Contactin-2  AQP1.193-215  HSP70  Viral.CMV-pp65  Myelin.MOG.88-134  HIF-1alpha  Myelin.MOG.88-134  Viral.CMV-pp65  Mimotop76  Quinta.Pos.IgG.PLP.215-232  HIF-1alpha  Contactin-2  Kir4.1.83-120  Viral.EBV-EBNA1  Mimotop63  Mimotop59  Mimotop80  AQP4.129-163  Viral.EBV-EBNA1  Mimotop71  AQP4.198-239  Viral.VZV-GlycoproteinC  NOGO-A  AQP4.198-239  Viral.CMV-pp65  Mimotop61  Mimotop52  Mimotop89  Viral.EBV-EBNA1  Viral.CMV-pp65  Mimotop91  Mimotop85  Mimotop7  Mimotop81  Viral.CMV-EnvelopeGlycoproteinC  Myelin.CNP  AQP4.198-239  Mimotop1  Viral.CMV-pp65  Viral.EBV-EBNA1  AN-2  AQP4.198-239  Myelin.MOG.88-134  Mimotop141  Viral.EBV-EBNA1  Mimotop65  Viral.CMV-EnvelopeGlycoproteinC  Viral.VZV-GlycoproteinC  Viral.EBV-EBNA1  Viral.EBV-EBNA1  Mimotop137  Mimotop78  Quinta.Pos.IgG.HSP60.240-259  Mimotop67  Mimotop66  Neurofascin186  Myelin.PLP  HIF-1alpha  Quinta.Pos.IgG.OSP.1-20  Viral.VZV-GlycoproteinC  Viral.VZV-GlycoproteinC  Neurofilament68kD  Viral.VZV-GlycoproteinC  Viral.EBV-EBNA1  Quinta.Pos.IgG.OSP.61-80  AmyloidBeta  Mimotop55  Mimotop128  Viral.EBV-EBNA1  Contactin-2  Mimotop103  Viral.VZV-GlycoproteinC  Kir4.1.83-120  Mimotop138  AQP4.46-78  Viral.VZV-GlycoproteinC  Viral.VZV-GlycoproteinC  aBCrystallin  IFNAR1  Quinta.Pos.IgG.OSP.61-80  AQP4.129-163  Viral.EBV-EBNA1  EAAT2  Mimotop40  Kir4.1.83-120  IFNAR1  Neurofascin186  AQP4.46-78  NOGO-A  Viral.EBV-EBNA1  Viral.VZV-GlycoproteinC  AQP4.129-163  Mimotop111  Viral.VZV-GlycoproteinC  Viral.EBV-EBNA1  Neurofilament68kD  Viral.VZV-GlycoproteinC  Viral.EBV-EBNA1  AQP4.46-78  Mimotop47  Viral.EBV-EBNA1  AQP4.198-239  Quinta.Pos.IgG.OSP.1-20  Viral.CMV-pp65  Mimotop21  Quinta.Pos.IgG.OSP.166-185  Mimotop105  bax  Viral.EBV-EBNA1  Viral.EBV-EBNA1  AQP4.198-239  Mimotop131  Glutaminsynthetase  Viral.EBV-EBNA1  NOGO-A  Mimotop150  Viral.EBV-EBNA1  Viral.CMV-pp65  Viral.EBV-EBNA1  AN-2  Viral.EBV-EBNA1  Myelin.MOG.27-57  Viral.EBV-EBNA1  Viral.EBV-EBNA1  Mimotop126  AQP1.193-215  AQP4.198-239  Mimotop139  Kir4.1.83-120  Viral.VZV-GlycoproteinC  Mimotop2  Mimotop108  AQP1.29-56  Viral.VZV-GlycoproteinC  Myelin.MOG.27-57  AmyloidBeta  Viral.CMV-pp65  Contactin-2  Kir4.1.83-120  Mimotop113  Myelin.MOG.88-134  AQP4.198-239  Viral.EBV-EBNA1  Viral.VZV-GlycoproteinC  aBCrystallin  AQP1.29-56  Viral.EBV-EBNA1  Quinta.Pos.IgG.HSP60.240-259  Viral.EBV-EBNA1 | 10.2054  10.2422  10.4074  10.2763  10.3734  10.1685  10.3272  10.2874  10.2658  10.1762  10.2879  10.1780  10.0525  10.3333  10.3357  10.2934  10.3699  10.2143  10.1546  10.4520  10.1986  10.1087  10.1431  10.2707  10.3609  10.3322  10.2082  10.3548  10.2444  10.2767  10.1701  10.2763  10.2802  10.2878  10.2246  10.0943  10.2042  10.6205  10.3826  10.1721  10.2338  10.2445  10.2260  10.4020  10.2879  10.3204  10.4142  10.1542  10.0747  10.1625  10.2559  10.2909  10.1520  10.2677  10.2746  10.3025  10.2862  10.1696  10.3887  10.0674  10.1848  10.2205  10.0250  10.1385  10.3552  10.1238  10.1783  10.3928  10.1733  10.3716  10.3278  10.1857  10.0847  10.2062  10.1888  9.9954  10.3126  10.2493  10.1594  10.2320  10.1511  10.3616  10.2765  10.2988  10.3003  10.1630  10.1454  10.2936  10.2768  10.2163  10.4082  10.5003  10.1924  10.3789  10.3076  10.2913  10.1550  10.1602  10.2052  10.3197  9.9992  10.2644  10.0971  10.3536  10.1844  10.0939  10.3241  10.0800  10.2182  10.2806  10.2792  10.2588  10.2288  10.2602  10.3329  10.2124  10.2359  10.1211  10.1102  10.1415  10.1848  10.1535  10.1815  10.0603  10.1903  10.1845  10.3038  10.1281  10.3465  10.3285  10.1961  10.3275  10.1420  10.0534  10.1854  10.0620  10.0851  10.1963  10.1521  10.1405  10.0566  10.1732  10.4615  10.1286  10.2418  10.2368  10.1580  10.3020  10.0818  10.1423  10.2149  10.0473  10.1191  9.9522  10.1808  10.2299  10.2166  9.9794  10.2997  10.1871  10.2743  10.0378  10.2343  10.1853  10.1197  10.3650  10.2801  10.3667  10.2200  10.1937  10.1553  10.2752  10.0032  10.9621  10.1135  10.1137  10.1391  10.2017  10.3179  10.1029  10.3361  10.2074  10.1228  10.1361  10.1201  10.1567  10.0436  10.2013  9.9795  10.0822  10.3240  10.1119  10.2585  10.2788  10.1984  10.0461  10.1689  10.3539  10.5749  10.9509  10.3206  10.0990  10.1726  10.0873  10.0380  10.0700  10.2054  10.3628  10.0646  10.2021  10.0528  9.9858  10.9623  10.2172  11.2002  10.2372  10.1229  10.3242  10.2653  10.3471  10.2197  10.1743  10.0075  10.9863  10.1424  10.1436  10.2042  11.0691  10.9985  10.1535  10.4835  10.0143  9.9416  10.2344  10.2378  10.2988  10.0075  10.4880  10.3325  10.2076  10.1352  10.7408  11.1333  10.0480  10.0516  11.2371  10.2875  10.3183  10.0674  10.3337  10.2322  10.3378  10.6595  9.9755  10.1663  10.0476  10.2331  10.1645  10.0451  10.2513  10.1737  10.0379  10.1368  10.0899  10.4058  10.3961  10.3364  10.2116  10.0813  10.3157  10.2319  10.1181  10.9986  10.3040  10.3445  10.6180  10.0954  10.1456  10.1373  10.1090  9.9385  11.0531  10.2223  10.1876  9.9238  10.1910  10.0912  10.0615  10.2332  10.3652  10.0721  10.1824  10.1121  10.2502  10.7999  10.3310  10.2372  10.0315  10.5667  10.1186  10.7811 | 10.5810  10.5983  10.8349  10.5301  10.7274  10.5262  10.7174  10.6028  10.4677  10.5748  10.6683  10.5183  10.3759  10.5978  10.6596  10.5749  10.7405  10.5258  10.4897  10.8756  10.5168  10.3909  10.3568  10.6558  10.6101  10.6326  10.5921  10.7017  10.6182  10.5950  10.4822  10.6476  10.8151  10.5964  10.4468  10.5644  10.3874  11.0524  10.7865  10.5267  10.6530  10.4890  10.5482  10.7386  10.6018  10.6023  10.7160  10.4108  10.2224  10.3368  10.4618  10.6392  10.3512  10.5700  10.6304  10.5541  10.5354  10.4419  10.6621  10.3880  10.4917  10.5088  10.1893  10.3712  10.6661  10.2965  10.5200  10.7021  10.4505  10.7305  10.5070  10.4093  10.2789  10.4845  10.3578  9.8587  10.5264  10.5643  10.3403  10.3787  10.4578  10.5938  10.4310  10.5635  10.4826  10.4364  10.2915  10.4624  10.4925  10.4027  10.6637  10.6908  10.3629  10.6651  10.4893  10.4431  10.3482  10.3606  10.4218  10.7069  10.1302  10.4846  10.2882  10.5014  10.4389  10.2459  10.5709  10.2447  10.6139  10.5424  10.5183  10.3963  10.4955  10.6329  10.5146  10.3841  10.4906  10.2558  10.2607  10.4487  10.3666  10.3853  10.3725  10.3158  10.3808  10.0973  10.5105  10.3030  10.5545  10.6134  10.4971  10.6214  10.3048  10.1501  10.7682  10.1545  10.2427  10.3237  10.4786  10.2450  10.2113  10.3739  10.8231  10.3455  10.3888  10.3582  10.2753  10.6630  10.1714  10.2811  10.6176  10.1584  10.2158  10.0835  10.2992  10.5914  10.1169  10.2361  10.5145  10.8901  10.5082  9.9607  10.3369  10.3809  10.2408  10.2916  10.4181  10.5707  10.3520  10.0937  10.0365  10.3809  9.9084  12.2442  10.2133  10.2174  10.2541  10.3293  10.4525  10.0190  10.4656  10.9457  10.2023  10.0615  10.3071  10.2381  9.9620  10.3161  10.0863  10.1583  11.6046  10.2078  10.4952  10.4152  10.5482  10.2312  10.0719  10.5624  12.2796  12.1162  10.2010  10.2403  10.2925  10.2469  9.9415  10.1715  10.4199  10.5444  10.2378  10.1173  10.1352  9.9214  12.0616  10.3049  12.3539  10.1690  10.2453  10.4716  10.3957  10.5000  10.3358  10.2948  10.1243  12.1301  10.2592  10.6139  10.1215  12.1381  12.0572  10.2778  11.4033  10.1382  9.8722  10.1698  10.9192  10.4563  10.1049  11.4655  10.5006  10.2747  10.2723  11.6224  12.2079  9.9905  10.1692  12.3196  10.1307  10.3791  10.1272  10.2785  10.1728  10.6327  11.2444  10.0377  10.2367  10.0957  10.3539  10.2391  9.9380  10.3191  10.1144  9.9696  10.0820  10.2294  10.5095  10.2541  10.4954  10.5871  10.1352  11.0229  10.1733  10.2738  12.0386  10.6483  10.2855  11.0928  10.0131  10.2108  10.0580  10.2157  10.0456  11.9001  10.1660  10.3357  9.8543  10.1317  10.0441  10.2410  10.4692  10.5976  10.1399  10.2914  10.0589  10.1707  11.2894  10.4312  10.3713  9.9854  10.9459  10.0265  11.3046 | 0.0001  0.0001  0.0001  0.0001  0.0001  0.0001  0.0001  0.0001  0.0001  0.0001  0.0001  0.0001  0.0001  0.0001  0.0001  0.0001  0.0001  0.0001  0.0001  0.0001  0.0001  0.0001  0.0001  0.0001  0.0001  0.0001  0.0001  0.0001  0.0001  0.0001  0.0001  0.0001  0.0001  0.0001  0.0001  0.0001  0.0001  0.0001  0.0001  0.0001  0.0001  0.0001  0.0001  0.0001  0.0001  0.0001  0.0001  0.0001  0.0001  0.0001  0.0001  0.0001  0.0001  0.0001  0.0001  0.0001  0.0001  0.0001  0.0001  0.0001  0.0001  0.0001  0.0001  0.0001  0.0001  0.0001  0.0001  0.0001  0.0001  0.0001  0.0001  0.0001  0.0001  0.0001  0.0001  0.0001  0.0001  0.0001  0.0001  0.0001  0.0001  0.0001  0.0001  0.0001  0.0001  0.0001  0.0001  0.0001  0.0001  0.0001  0.0001  0.0001  0.0001  0.0001  0.0001  0.0001  0.0001  0.0001  0.0001  0.0001  0.0001  0.0001  0.0001  0.0001  0.0001  0.0001  0.0001  0.0001  0.0001  0.0001  0.0001  0.0001  0.0001  0.0001  0.0001  0.0001  0.0001  0.0001  0.0001  0.0001  0.0001  0.0001  0.0002  0.0002  0.0002  0.0002  0.0002  0.0002  0.0002  0.0002  0.0002  0.0002  0.0002  0.0003  0.0003  0.0003  0.0003  0.0003  0.0003  0.0003  0.0004  0.0004  0.0004  0.0004  0.0004  0.0005  0.0005  0.0005  0.0007  0.0008  0.0009  0.0011  0.0011  0.0012  0.0013  0.0014  0.0014  0.0014  0.0014  0.0014  0.0015  0.0015  0.0015  0.0016  0.0019  0.0020  0.0020  0.0020  0.0022  0.0022  0.0023  0.0023  0.0026  0.0029  0.0029  0.0031  0.0031  0.0032  0.0032  0.0035  0.0036  0.0036  0.0036  0.0037  0.0037  0.0037  0.0037  0.0041  0.0043  0.0043  0.0044  0.0044  0.0044  0.0046  0.0050  0.0055  0.0056  0.0057  0.0057  0.0066  0.0067  0.0069  0.0069  0.0071  0.0072  0.0073  0.0073  0.0075  0.0075  0.0076  0.0076  0.0076  0.0077  0.0077  0.0077  0.0077  0.0077  0.0078  0.0085  0.0090  0.0091  0.0091  0.0092  0.0096  0.0096  0.0100  0.0100  0.0103  0.0103  0.0103  0.0105  0.0112  0.0123  0.0127  0.0127  0.0133  0.0134  0.0137  0.0141  0.0145  0.0147  0.0148  0.0149  0.0151  0.0161  0.0166  0.0166  0.0169  0.0171  0.0171  0.0172  0.0181  0.0192  0.0195  0.0195  0.0196  0.0200  0.0206  0.0213  0.0219  0.0221  0.0238  0.0241  0.0241  0.0247  0.0249  0.0250  0.0256  0.0257  0.0257  0.0260  0.0260  0.0279  0.0282  0.0282  0.0284  0.0299  0.0299  0.0299  0.0299  0.0308  0.0335  0.0337  0.0346  0.0357  0.0357  0.0357  0.0358  0.0358  0.0360  0.0399  0.0417  0.0422  0.0427  0.0427  0.0429  0.0456  0.0474  0.0474  0.0482  0.0494 |
| **Peptide** | **Annotation Protein** | **Mean pattern III** | **Mean Sjögren´s syndrome** | **P value** |
| PEDDGEYWCVAENQY  ALLDVWNLEFMEHIY  QMPYQDITAFVEHDF  GYFEDRRPSANCDPF  AGCRLEEEEYEDDAY  KVETMRYEDHDWTLY  PYIPMDDDFQLRSFD  MVWALDLDDFQGSFC  GPIIGAVLAGGLYEY  NTVIIPTGWWDVGYY  PWGRLCRDWDQMFAF  RYNWIYIDNIPELTF  PLVRRIISEWFEEVY  SPITDYVVQFEEDQF  QIPSVSFEDEGTYEC  SALGSQTTFGPVFED  FQVMQAFPISYEQDY  TLDNDGDGECDFQEF  PELPYNNDLEVHYPY  HNEPETENHLFYDFV  TFDVSILTIDDGIFE  AGPQGWHMSEFLWYY  SGTIGYGFRYISEEC  GFYTTGAVRQIFGDY  ATVQGQNLKYQEFFW  RRLAGQFLEELRNPF  PKEEDGSFDYSDEDN  QRFSDGYDNGVRHDF  HLHTWIQDNGGWDAF  LEETLRNLQARYEEE  RVILYNRTRLDCPFF  AVLAGGLYEYVFCPD  LIFGSRQYQEGYYYN  FVNPIIQHDPNYDII  LEMLAPYIPMDDDFQ  QYQNENITWDYTPEY  LDDTNHERERLEQLF  TDWSYGPMIWRDWFY  VLFTSDEHQVDMEWF  SLFARRSGNERIWYD  CLQVVGFVTSFVGWI  VWIWERDIYHPGDPR  MVATCLQVVGFVTSF  SLVNTNAWVQRDMYF  TCTAAAYYPRNPVEF  WKHNFGPGTDFVVEY  QLAGAMVWALDLDDF  TSDTYPNGVIVWPEF  FLRVPCWKITLFVIV  YHCKPLVDILILPGY  IEGMKFDRGYISPYF  SFDDGVDLPPWFPPM  IIMIVISIDYSMNDF  RLEDEQVEQDYFAGW  KLTHDVELNLDYERY  IILDVRAIPIYIDEV  HPHRVMQSVFDREWF  AGDAQAFGENRFYYT  YGGCGGNRNNFDTEE  SDTVQGQLREYRAYY  HLVPGPWGRLCRDWD  FPPFSAVYTYDASTY  GTYIWRVVNTKTKNV  SYMQTPRHDEYFAGF  YQARTALWEIVHHDW  KLKPGLEKDFLPLYF  GESYEDPPQEEYQEY  LRITNVSEEDSGEYF  DLERKIESLEEEIRF  RPGNQEPFVRWRDYW  WNTWPYYERFEHQFN  PTCNIRVTVCSFDDG  TSHIVARDWGTFEYY  TDDSGTSHYDVMDYE  AYYPRNPVEFVWFED  RLWNTAYSGHEVEYR  YKITPGARGAFSEEY  WEWYTYSKNEWEIFL  VCYFMVFLQTHIFAE  QAHWENTRNEEQHWD  YNNSTFVMNQFVDFW  VVQRSDWAPQPQYNE  WMMPSVNDYDDNANY  LQQFQKEDAALTIYE  NQQTFLNRAVSYQEF  QTTIGYGFRYISEEC  PAVIMGNWENHWIYW  LLFLLEEYKNYLDAA  LEQLFEMADQYQYQV  GYEPEELLGRSIYEY  RLGVRIAHYYEVEPF  ARHTNSWEWLKYEYD  PSIELRDVRYYLDEF  NVRFSDEGGFTCFFR  TSYRDDEMSRPQWEW  PGLSYRWLLNEFPNF  SGSGMSSFSYEPYYS  AFLERADSVISWDIQ  RFSDEGGFTCFFRDH  FGPAVIMGNWENHWI  GNWENHWIYWVGPII  KRPRSPSSQSSSSGS  PEPESVIGYSGEDYP  NPVEFVWFEDDHQVF  AVTHGMNYGRMPFDF  KLIETYFSKNYQDYE  QAGDDFSRRYRRDFA  QVSGYVDWGLRWFEM  YPVGNNQTAVQDNVK  TILSMDATTEGTYIW  TFPIPSTVISWNDAA  AYVLLSEKKISSIQS  EWTIFLYPNQEQPEW  SLSPLSAASFKEHEY  ARTGRPQQEPVWNYD  IGSALGFKYPVGNNQ  PARSFGPAVIMGNWE  AETYALVGQQVTLEC  LVDSKSQHMWPADVF  DHMAWEEQEGELDFQ  VPPEFKPDHFRDHEY  TVELRQYDPVAALFF  GVVWYLVAVAHGDLL  DPHLCDFIETHYLNE  AAPANAPNFPEGEYH  YVVQFEEDQFQPGVW  VIMGNWENHWIYWVG  LSAEPAPDFSDYSEM  GQSALNPYNYRVNNY  RRSTSDNNTTHLSSM  EVEEEEADDDEDDED  DGMYDIPSISEYYTY  QAIRETVELRQYDPV  PSSQSSSSGSPPRRP  QLQNNTLEGEQYTEY  RNLQARYEEEVLSRE  AEGDDGDDGDEGGDG  AGNILYEHEMPPEPF  AETAQVLGLTPWMDY  KTFMVYSAHSGNGKY  HIDTWEWNDVTLYGM  ETGPCRAMISRWYFD  SFEDEGTYECEAENS  GPPPGGQPDPELLQF  HFLRVPCWKITLFVI  GNTPVIQDYPLIYEI  GLDRTGKGERNVLIF  IRILVLDDTNHERER  GSTMDIDHEWERVQF  DFEVVTFLKDVLPEF  GDANNSPITDYVVQF  GKVCGSNLLSICKTA  HMQIERWYNFGEEDR  PDSWSPMDASDDIFI  SLGSTINWGGTEKPL  YNPASPDFEEFDSAV  DRHDEGAAQGDDDVW  IDFRSGGRPEEYEGE  NAEHNHSEQSAPQMG  NISTIATVEETNQTD  YLVAVAHGDLLELDP  WFGKHRGQGGSNPKF  GPRGQGDGGRRKKGG  AGGAGGAGAGGAGGA  QPGNTISAGQEDFPS  DRRRHIVEIVTWERY  LFSYVSAVRIPQQKQ  EEEEYEDDAYGHYEA  CLVLICEPIPHPSNI  SDVWWGGADTDYADG  IETYDGVQDFNYLTW  LVTPPSVVGGLGVTM  PAAGAPLMDFGNDFV  TWYWMYYGASLSSEW  VERTTDEGTWVAGVF  CFIIENISTIATVEE  EAGTLAYYEICDFLR  PAWEPHRYVARLFEL  QVTFTVWQIYEIDYS  NNQTAVQDNVKVSLA  GGCVILCCAGDAQAF  MREQTLYVMIREPYR  SSPEPNSPSEYCFYV  RGRGGSGGRGRGGSG  WARLPSAPPTAFQER  RWVSQSVNIPTAYEV  TPLSRLPFGMAPGPG  LLSLGSTINWGGTEK  GGLGVTMVHGNLTAG  GSPNKHDWGDDRPDN  GAGGAGGAGAGGAGG  LKAESTVAPEEDTDE  SDMYLVPAAMFRDPF  VVENVNDENDFLSWF  RVEDPYQWTSASDAF  LDNLPNRWDASDYDD  TSGSDSDEELVTTER  LGFKYPVGNNQTAVQ  GRTELLKDAIGEGKV  RSPGWETFMNTGFIH  DDVVQWMLSIERPNF  ADGVNSGQGLGIEII  WNGDHENHMRFNNVD  HMQSSEHAQRRGDYP  YQDYEYLINVIHAFQ  FPGKVCGSNLLSICK  CIGCKGTHGGTGAGA  AGAGGGAGGAGGAGA  GAGGGAGGAGAGGAG  SDEELVTTERKTPRV  GHLFAINYTGASMNP  GAGAGGAGAGGAGGA  TYVSMVYYIQNPIYT  SELVEDSSPDSEPVD  AGAGGGAGGAGGAGG  KPLPVDMVLISLCFG  VYGGSKTSLYNLRRG  VRDHVGSILQNNQSN  RYEEEVLSREDAEGR  AFFNVRKNNQDQKMQ  AGGAGGAGGAGAGGA  SDEGGFTCFFRDHSY  DNNTTHLSSMESVHN  LMRWSANEHDATLDQ  GKVTLRIRNVRFSDE  AAAAAPLGGGGGGSG  LGVTMVHGNLTAGHG  VVGGLGVTMVHGNLT  GAGGAGAGGAGGAGA  SGGRGRGGSGGRGRG  FGSAVITHNFSNHWI  AAFKQQQKTYEQYSL  GAGAGGGAGGAGGAG  VEKSRAWISDGNNRF  HGRGRGRGRGRGGGR  SAVITHNFSNHWIFW  SRHSLDMKFSYCDER  GGGAGAGGAGGAGGA  RGGSGGRGRGGSGGR  RKKGGWFGKHRGQGG  SANATTMKPPPRRPP  GGAGGAGAGGGAGGA  VYPELQITNVVEANQ  LGNYSCLATSHMDFS  SALGFKYPVGNNQTA  HQPPPRDPTERQVIE  IEYQPMVDAEEPDWQ  GGKPNHPSEVDIYRE  IRNVRFSDEGGFTCF  GSGGRRGRGRERARG  VLKDAIKDLVMTKPA  AGGGGRGRGGSGGRG  GKNATGMEVGWYRPP  PQPGPLRESIVCYFM  DSDMVNEFKLELVEK  GAGAGGGAGGAGAGG  AGGAGAGGGAGGAGG  PSVVGGLGVTMVHGN  EEPEAAYRLIQGPQY  GAGAGGAGGAGAGGA  GGAGAGGGAGAGGAG  RTDVTGSIALAIGFS  DLAQESVDTVGDPID  DEGEEGQEGSGSGSG  DDFPIDFDKPGGHYR  GAGGGAGGAGGAGAG  AIGHLFAINYTGASM  HPDYHLDHNYMLVER  TFDPHFLRVPCWKIT  HYREDAALDQEQSMV  FVLLSLGSTINWGGT  GLRALLARSHVERTT  MTRGRLKAESTVAPE  ATAILSGITSSLTGN  GAGGAGGAGAGGGAG  APKQSGNTPVIQDYP  KDAIGEGKVTLRIRN  ADIPTDAAEKWQYPE | Myelin.MAG  Mimotop5  Mimotop32  Glutaminsynthetase  AN-2  Mimotop41  HIF-1alpha  CHI3L1  AQP4  Mimotop13  CYP27B1  Mimotop136  Mimotop16  Neurofascin186  Contactin-2  Contactin-2  Mimotop153  S-100b  Mimotop44  Mimotop135  HSP70  Mimotop121  Kir4.1  Myelin.PLP  Viral.CMV-pp65  Myelin.MOG  Neurofascin186  Mimotop133  bcl-2  Neurofilament68kD  Neurofascin186  AQP4  Mimotop118  Mimotop158  HIF-1alpha  Mimotop119  Myelin.CNP  Mimotop149  Mimotop130  Mimotop151  Quinta.Pos.IgG.OSP.1-20  Mimotop8  Quinta.Pos.IgG.OSP.1-20  Mimotop34  Viral.HSV-GlycoproteinC  Neurofascin186  CHI3L1  Mimotop51  Quinta.Pos.IgG.MOG.196-215  Quinta.Pos.IgG.OSP.61-80  HSP60  Viral.EBV-EBNA1  Mimotop134  Mimotop48  sTFR  Mimotop147  Mimotop155  Myelin.OSP  AmyloidBeta  Neurofascin186  CYP27B1  Viral.VZV-GlycoproteinC  Viral.VZV-GlycoproteinC  Mimotop68  Mimotop125  Myelin.CNP  Beta-Synuclein  Neurofascin186  GFAP  Mimotop10  Mimotop58  Viral.EBV-EBNA1  Mimotop11  Mimotop56  Viral.HSV-GlycoproteinC  Mimotop38  Myelin.CNP  Mimotop46  Viral.EBV-EBNA1  Mimotop127  Mimotop50  Mimotop114  Mimotop4  Type1interferon  Mimotop120  Kir4.1  AQP4.198-239  BDNF  Myelin.CNP  HIF-1alpha  Mimotop117  Mimotop43  Mimotop15  Myelin.MOG.88-134  Mimotop148  Contactin-2  Neurofilament68kD  Viral.CMV-EnvelopeGlycoproteinC  Myelin.MOG.88-134  AQP4.198-239  AQP4.198-239  Viral.EBV-EBNA1  Neurofascin186  Viral.HSV-GlycoproteinC  Mimotop152  Myelin.PLP  bcl-2  Mimotop54  AQP1.29-56  Viral.VZV-GlycoproteinC  Mimotop39  Quinta.Pos.IgG.HSP60.240-259  Mimotop122  NOGO-A  Mimotop57  AQP1.29-56  AQP4.198-239  Contactin-2  Mimotop72  Mimotop146  Mimotop6  Viral.CMV-pp65  Kir4.1.83-120  FerritinHeavyChain  Mimotop62  Neurofascin186  AQP4.198-239  NOGO-A  Mimotop145  Viral.CMV-EnvelopeGlycoproteinC  AmyloidBeta  Mimotop154  Viral.CMV-pp65  Viral.EBV-EBNA1  Mimotop14  Neurofilament68kD  Viral.EBV-EBNA1  AN-2  Contactin-2  Mimotop85  CHI3L1  AmyloidBeta  Contactin-2  AN-2  Quinta.Pos.IgG.MOG.196-215  IFNAR1  HSP70  Myelin.CNP  Mimotop49  Viral.Peptid Adenovirus type 12, ORF  Neurofascin186  Quinta.Pos.IgG.PLP.215-232  Mimotop52  Mimotop65  AQP4.46-78  Mimotop132  Viral.CMV-pp65  Neurofascin186  Mimotop105  IFNAR1  Kir4.1.83-120  Viral.EBV-EBNA1  Viral.EBV-EBNA1  Viral.EBV-EBNA1  NOGO-A  Mimotop45  Viral.VZV-GlycoproteinC  AN-2  HIF-1alpha  AmyloidBeta  Mimotop47  AQP4.129-163  NOGO-A  Mimotop59  Viral.EBV-EBNA1  IFNAR1  CHI3L1  Mimotop76  Mimotop12  AQP1.29-56  Quinta.Pos.IgG.OSP.166-185  Mimotop71  HIF-1alpha  Viral.EBV-EBNA1  Mimotop113  Mimotop138  Viral.EBV-EBNA1  AQP4.46-78  AQP4.129-163  Mimotop42  Viral.EBV-EBNA1  Viral.CMV-pp65  Glutaminsynthetase  Mimotop53  Mimotop75  Mimotop156  Viral.CMV-pp65  AQP1.29-56  Myelin.MOG.88-134  Mimotop91  Mimotop137  AQP1.108-144  Mimotop141  Mimotop104  Myelin.PLP  Quinta.Pos.IgG.PLP.215-232  Viral.EBV-EBNA1  Viral.EBV-EBNA1  Viral.EBV-EBNA1  Viral.CMV-pp65  AQP4.198-239  Viral.EBV-EBNA1  Mimotop67  NOGO-A  Viral.EBV-EBNA1  AQP4.46-78  Viral.EBV-EBNA1  Mimotop83  Neurofilament68kD  Mimotop89  Viral.EBV-EBNA1  Myelin.MOG.88-134  Viral.CMV-EnvelopeGlycoproteinC  Mimotop1  Myelin.MOG.88-134  Olig2  AQP4.129-163  AQP4.129-163  Viral.EBV-EBNA1  Viral.EBV-EBNA1  AQP1.193-215  Viral.VZV-GlycoproteinC  Viral.EBV-EBNA1  Mimotop80  Viral.EBV-EBNA1  AQP1.193-215  HIF-1alpha  Viral.EBV-EBNA1  Viral.EBV-EBNA1  Viral.EBV-EBNA1  Contactin-2  Viral.EBV-EBNA1  AmyloidBeta  Contactin-2  AQP1.29-56  Viral.HSV-GlycoproteinC  Mimotop128  Mimotop61  Myelin.MOG.88-134  Viral.EBV-EBNA1  Viral.EBV-EBNA1  Viral.EBV-EBNA1  Myelin.MOG  Viral.EBV-EBNA1  HIF-1alpha  Viral.EBV-EBNA1  Viral.EBV-EBNA1  AQP4.129-163  AN-2  Viral.EBV-EBNA1  Viral.EBV-EBNA1  AQP4.198-239  Mimotop74  Viral.EBV-EBNA1  Contactin-2  Viral.EBV-EBNA1  AQP4.198-239  Mimotop66  Quinta.Pos.IgG.MOG.196-215  Mimotop126  AQP4.46-78  Viral.EBV-EBNA1  Viral.CMV-pp65  AQP1.108-144  Viral.EBV-EBNA1  IFNAR1  Myelin.MOG.88-134  Mimotop21 | 10.2832  10.2729  10.1474  10.2571  10.3523  10.2938  10.3469  10.3472  10.3118  10.4586  10.4067  10.2059  10.3942  10.2573  10.2997  10.2255  10.2441  10.3788  10.2190  10.1180  10.2291  10.4974  10.2950  10.2087  10.3437  10.1912  10.1303  10.1964  10.3078  10.2205  10.3154  10.2581  10.3115  10.1024  10.2453  10.2510  10.1471  10.6580  10.3929  10.3549  10.1752  10.2678  10.0862  10.3468  10.1385  10.1788  10.4296  10.3228  10.0307  10.1656  10.3113  10.3330  10.2703  10.3024  10.2942  10.1928  10.3997  10.0920  10.1330  10.2946  10.4523  10.1662  9.9918  10.2621  10.0927  10.3016  10.2573  10.2616  10.2964  10.4644  10.1637  10.1987  10.3186  10.1982  10.4022  10.2824  10.3335  10.4007  10.0371  10.2016  10.3452  10.0959  10.2202  10.0913  10.2437  10.2987  10.4312  10.1572  10.1116  10.3362  10.2215  10.3651  10.4069  10.3017  10.4422  10.2307  10.3314  10.2012  10.1687  10.2219  10.2528  10.1891  10.2937  10.2795  10.3399  10.2578  10.2137  10.5267  9.9787  10.2557  10.0717  10.0760  10.2908  10.1149  10.2342  10.0709  10.3718  10.1281  10.1945  10.2360  10.1606  10.2169  10.0630  10.0736  10.1027  10.3221  10.3408  10.3450  10.0595  10.0603  10.2784  10.3036  10.1212  10.2626  10.2086  10.1928  10.3962  10.2024  10.4212  10.1482  10.2052  10.4486  10.1757  10.2291  10.0299  10.1835  10.1799  10.1877  10.2483  10.2459  10.1053  10.0376  10.3083  10.3258  10.2212  10.2759  10.2436  10.3358  10.0803  10.2506  10.1215  10.2149  10.3343  10.5644  10.1123  10.3801  9.9938  10.2483  9.9978  10.2981  10.2674  10.1879  10.2918  10.3798  10.2075  10.3671  10.3936  10.2985  10.2743  10.0523  10.1789  10.2828  10.2156  10.3436  10.1441  10.1065  10.2416  10.2414  10.0556  10.1671  10.5855  10.3762  10.3843  10.5031  10.2206  10.3629  10.2931  9.9267  10.1999  10.1302  10.1765  10.1890  10.0576  10.1694  10.0702  10.1567  10.1530  10.6086  10.7239  10.2984  9.9886  10.6299  10.0756  10.1307  10.5488  10.2699  10.2336  10.0082  10.3153  10.0910  10.4817  10.2360  9.9339  10.0918  10.1083  10.3497  9.9401  10.0359  10.4831  10.3493  9.9850  10.0667  10.4911  10.2486  10.3497  10.1979  10.2600  10.5326  10.4406  10.1756  10.1836  11.0560  10.0407  10.1459  9.9553  10.0968  10.3637  10.2289  10.3591  10.3191  10.1525  10.3286  10.3567  10.3642  10.1897  10.5325  10.8346  10.0704  10.1845  10.5560  11.0298  10.0677  10.1554  10.4289  10.1930  10.5275  10.1351  10.2450  10.1603  10.0956  10.1994  10.0349  10.3417  10.0604  10.9601  10.0866  10.1134  10.2451 | 10.5749  10.5983  10.4897  10.5810  10.6101  10.5301  10.6596  10.5978  10.6028  10.8349  10.7274  10.5262  10.7405  10.6182  10.8151  10.5168  10.6530  10.7174  10.5183  10.5644  10.5748  10.8756  10.4677  10.5088  10.6683  10.4027  10.3712  10.5267  10.6018  10.3787  10.6558  10.5258  10.6476  10.3759  10.4468  10.5921  10.3403  11.0524  10.7017  10.6661  10.3568  10.4618  10.2224  10.6023  10.3606  10.4419  10.7865  10.5950  10.1893  10.3512  10.5264  10.5070  10.5482  10.5354  10.5635  10.5200  10.6637  10.2789  10.2965  10.5700  10.7386  10.3909  9.8587  10.5105  10.3880  10.4893  10.5183  10.5643  10.4624  10.7160  10.4578  10.3578  10.6304  10.4505  10.7305  10.4310  10.5541  10.6326  10.1501  10.4108  10.6392  10.2882  10.4955  10.2459  10.4822  10.4890  10.6621  10.2915  10.3030  10.7069  10.6139  10.5964  10.8231  10.4431  10.7021  10.4906  10.5709  10.3368  10.3482  10.4093  10.3874  10.0365  10.5424  10.6329  10.5545  10.4845  10.3629  10.6908  9.8543  10.4917  10.1714  9.9415  10.4846  10.2607  10.4218  9.9854  10.5014  10.2558  10.3666  10.3888  10.4487  10.4364  10.1545  10.3158  10.2447  10.4925  10.4826  10.6134  10.2113  9.9620  10.4716  10.6630  10.2541  10.1733  10.3808  10.3049  11.6224  10.3725  10.5938  10.0615  10.3237  10.6651  10.3809  10.4971  10.1302  10.4786  10.2992  10.3161  10.3841  10.4389  10.2427  9.9607  10.4525  10.5624  10.1215  10.7682  10.5914  10.5444  9.9380  11.4655  10.2408  10.1079  10.2010  11.2894  10.2133  10.5146  9.9214  10.4952  10.2361  10.3963  10.6327  10.0937  10.4954  10.5707  10.3358  11.4033  10.6214  10.5082  10.6176  9.9444  10.2753  10.3809  10.3853  10.2785  10.2914  10.6139  10.1698  10.1728  9.9905  10.2811  11.3046  11.6046  10.5095  11.2344  10.3739  10.6552  10.8901  9.8205  10.1169  10.2023  10.2925  10.3048  10.2312  10.2450  10.2378  10.0973  10.0726  11.2444  11.4766  10.9457  9.9084  11.2850  10.1715  10.2723  11.0928  10.3582  10.1690  9.8845  10.3791  10.0190  10.9779  10.5482  9.8502  10.1583  10.1841  10.2510  9.8722  9.9701  11.0746  10.2916  10.0835  10.1272  10.9459  10.3520  10.2855  10.3455  10.3369  11.0003  10.3518  10.1144  10.0268  11.9664  10.2410  10.2158  9.8824  10.5886  10.5000  10.3293  10.5145  10.2254  10.0719  10.2761  10.2358  10.4656  10.1173  10.9828  11.4988  9.9921  10.3734  11.0843  11.7151  10.1583  10.4413  11.0644  10.2948  11.0549  10.0820  10.4199  10.0974  10.0131  10.1430  9.9696  11.0229  9.9935  11.7281  10.1584  10.0589  10.3539 | 0.0001  0.0001  0.0001  0.0001  0.0001  0.0001  0.0001  0.0001  0.0001  0.0001  0.0001  0.0001  0.0001  0.0001  0.0001  0.0001  0.0001  0.0001  0.0001  0.0001  0.0001  0.0001  0.0001  0.0001  0.0001  0.0001  0.0001  0.0001  0.0001  0.0001  0.0001  0.0001  0.0001  0.0001  0.0001  0.0001  0.0001  0.0001  0.0001  0.0001  0.0001  0.0001  0.0001  0.0001  0.0001  0.0001  0.0001  0.0001  0.0001  0.0001  0.0001  0.0001  0.0001  0.0001  0.0001  0.0001  0.0001  0.0001  0.0001  0.0001  0.0001  0.0001  0.0001  0.0001  0.0001  0.0001  0.0001  0.0001  0.0001  0.0001  0.0001  0.0001  0.0001  0.0001  0.0001  0.0001  0.0001  0.0001  0.0001  0.0001  0.0001  0.0001  0.0001  0.0001  0.0001  0.0001  0.0001  0.0001  0.0001  0.0001  0.0001  0.0001  0.0002  0.0002  0.0002  0.0002  0.0002  0.0002  0.0002  0.0002  0.0002  0.0002  0.0002  0.0003  0.0003  0.0003  0.0003  0.0003  0.0003  0.0004  0.0004  0.0004  0.0004  0.0004  0.0004  0.0004  0.0005  0.0005  0.0005  0.0005  0.0006  0.0006  0.0006  0.0006  0.0007  0.0007  0.0008  0.0009  0.0009  0.0009  0.0009  0.0011  0.0012  0.0012  0.0012  0.0012  0.0012  0.0013  0.0013  0.0013  0.0013  0.0017  0.0017  0.0018  0.0018  0.0019  0.0021  0.0023  0.0025  0.0025  0.0026  0.0026  0.0027  0.0028  0.0032  0.0034  0.0035  0.0035  0.0036  0.0036  0.0038  0.0038  0.0040  0.0040  0.0043  0.0044  0.0044  0.0049  0.0050  0.0054  0.0054  0.0064  0.0064  0.0064  0.0064  0.0065  0.0069  0.0072  0.0073  0.0073  0.0073  0.0074  0.0076  0.0078  0.0086  0.0088  0.0088  0.0092  0.0092  0.0093  0.0098  0.0098  0.0101  0.0103  0.0105  0.0105  0.0106  0.0111  0.0121  0.0129  0.0138  0.0138  0.0141  0.0141  0.0149  0.0157  0.0160  0.0164  0.0164  0.0164  0.0167  0.0167  0.0167  0.0177  0.0177  0.0177  0.0177  0.0178  0.0178  0.0178  0.0178  0.0181  0.0182  0.0186  0.0186  0.0194  0.0210  0.0211  0.0213  0.0221  0.0227  0.0230  0.0235  0.0239  0.0239  0.0244  0.0251  0.0252  0.0256  0.0257  0.0260  0.0260  0.0263  0.0265  0.0265  0.0269  0.0285  0.0286  0.0300  0.0301  0.0302  0.0305  0.0305  0.0319  0.0319  0.0322  0.0322  0.0325  0.0354  0.0360  0.0374  0.0378  0.0380  0.0384  0.0386  0.0390  0.0390  0.0393  0.0393  0.0393  0.0393  0.0407  0.0427  0.0441  0.0459  0.0477  0.0494  0.0495 |
